# Rapid and Universal Synthesis of 2D Transition Metal (Ti, Zr, Hf, V, Nb, Ta, Cr, Mo, and W) Sulfides through Oxide Sulfurization in CS_2_ Vapor

**DOI:** 10.1021/acs.inorgchem.4c00475

**Published:** 2024-04-24

**Authors:** Vladislav Buravets, Frantisek Hosek, Vasilii Burtsev, Elena Miliutina, Jaroslav Maixner, Ladislav Lapcak, Lucia Bajtosova, Miroslav Cieslar, Michal Procházka, Jan Minar, Zdenka Kolska, Vaclav Svorcik, Oleksiy Lyutakov

**Affiliations:** † Department of Solid State Engineering, University of Chemistry and Technology, Prague 166 28, Czech Republic; ‡ Central Laboratories, University of Chemistry and Technology, Prague 166 28, Czech Republic; § Faculty of Mathematics and Physics, Charles University, Prague 12116, Czech Republic; ∥ New Technologies−Research Centre, University of West Bohemia, Univerzitní 8, Plzeň 30614, Czech Republic; ⊥ CENAB, Faculty of Science, J. E. Purkyne University, Usti nad Labem 40096, Czech Republic

## Abstract

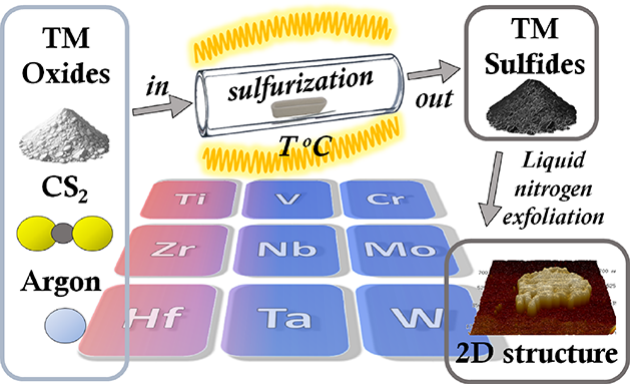

Transition metal (TM) sulfides belong to the class of 2D materials with a wide application range. Various methods, including solvothermal, hydrothermal, chemical vapor deposition, and quartz ampoule-based approaches, have been employed for the synthesis of TM sulfides. Some of them face limitations due to the low stability of TM sulfides and their susceptibility to oxidation, and others require more sophisticated equipment or complex and rare precursors or are not scalable. In this work, we propose an alternative approach for the synthesis of 2D TM sulfides by sulfurization of corresponding metal oxides in the vapor of CS_2_ at elevated temperature. Subsequent treatment in liquid nitrogen allows exfoliation of created sulfides to a 2D structure. A proposed approach was successfully applied to nine transition metals: Ti, Zr, Hf, V, Nb, Ta, Cr, Mo, and W. The resulting materials were extensively characterized using various analytical techniques with a focus on their crystalline structure and 2D nature. Our approach offers several advantages including the use of simple precursors (CS_2_ and metal oxides), universality (in all cases, the sulfides were obtained), equipment simplicity (tube furnace and quartz reactor), short preparation time (3 h), and the ability of morphology and phase tuning (in particular cases) of the created materials by adjusting the temperature. In addition, gram-scale bulk materials can be obtained in the entry-level laboratories using the proposed approach.

## Abstract 2

A universal approach for the synthesis of transition metal sulfides using sulfurization of metal oxides in CS_2_ vapor is proposed. Subsequent exfoliation in liquid nitrogen allows to reach a 2D structure of created materials. The universality of our methods was demonstrated on the following TMs: Ti, Zr, Hf, V, Nb, Ta, Cr, Mo, and W.

## Introduction

The 21st century started with increasing interest toward 2D nanomaterials exhibiting unique properties and a wide potential application range in electronics, energy, biomedicine, sensing, and optics.^1−3^ With their ultrathin profile, 2D materials have not only huge surface-to-volume ratio but also quantum confinement, which can drastically alter their physical, chemical, optical, thermal, electronic and mechanical properties.^4,5^ To date, there are many known classes of 2D materials, such as graphene, MXene, black phosphorus, and silicone just to name a few. Among them, transition metal chalcogenides (TMCs) constitute a wide class with a general formula of MX_2_ where M stands for transition metals (groups 4–10) and X for chalcogenide. The typical arrangement of atoms in TMCs comprises a monolayer of transition metal (TM) atoms sandwiched between two layers of chalcogen atoms, often referred to as a flake, with van der Waals forces formed between flakes.^6−8^ The diversity of chemical composition of TMCs as well as their crystalline structure provides extensive variability in their properties and potential applications.^3,9−12^ In particular, TMCs were found to be metallic,^6^ semimetallic,^13,14^ semiconductors,^15,16^ and insulators.^17^ Due to the outstanding properties, TMCs have found application in multiple disciplines including catalysis,^18−21^ energy storage,^22−24^ optoelectronic devices,^25,26^ and various other fields.^27−29^

Despite the growing interest toward TMCs, especially in 2D form, their synthesis still represents a challenge for the academia and even more so for the industry where scalability is a crucial factor.^2,30^ In general, there are two approaches to obtain 2D materials—top-down, consisting of bulk material exfoliation by physical or chemical means, or bottom-up approach, employing a solvothermal method or vapor-phase deposition with consequent chalcogenation.^31,32^ Most of TMs have high affinity to oxygen, and high energies are required to overcome the TM–oxygen bond, or the presence of oxygen has to be controlled.^33^ Thus, the synthesis of bulk TMCs is commonly performed in an ampoule sealed under vacuum containing the powdered metal of interest and chalcogenide, which are further heat-treated for days or even weeks to obtain TMC.^30^ On the other hand, the bottom-up approach is complicated by the choice of the correct precursor as many of the transition metal salts are hard to evaporate and are also prone for further oxidation in case oxygen is present in the system.^2^ Thus, further progress can significantly be boosted with the development of simple, reliable, and scalable synthesis methods, which can be further extended on various TMs, especially those with high oxygen affinity.

In this work, we expand our previously reported^34^ simple and scalable sulfurization method for a wide range of transition metals. Using CS_2_ as a sulfurizing agent, conversion of the metal oxides can be achieved due to its strong reducing and sulfurizing abilities. A simple setup allowed high yield and scalable synthesis of transition metal sulfides of 9 different elements (Ti, Zr, Hf, V, Nb, Ta, Cr, Mo, and W) with possible phase tuning by mere temperature variation.

## Results and Discussion

### Synthesis Overview

**Figure 1:**
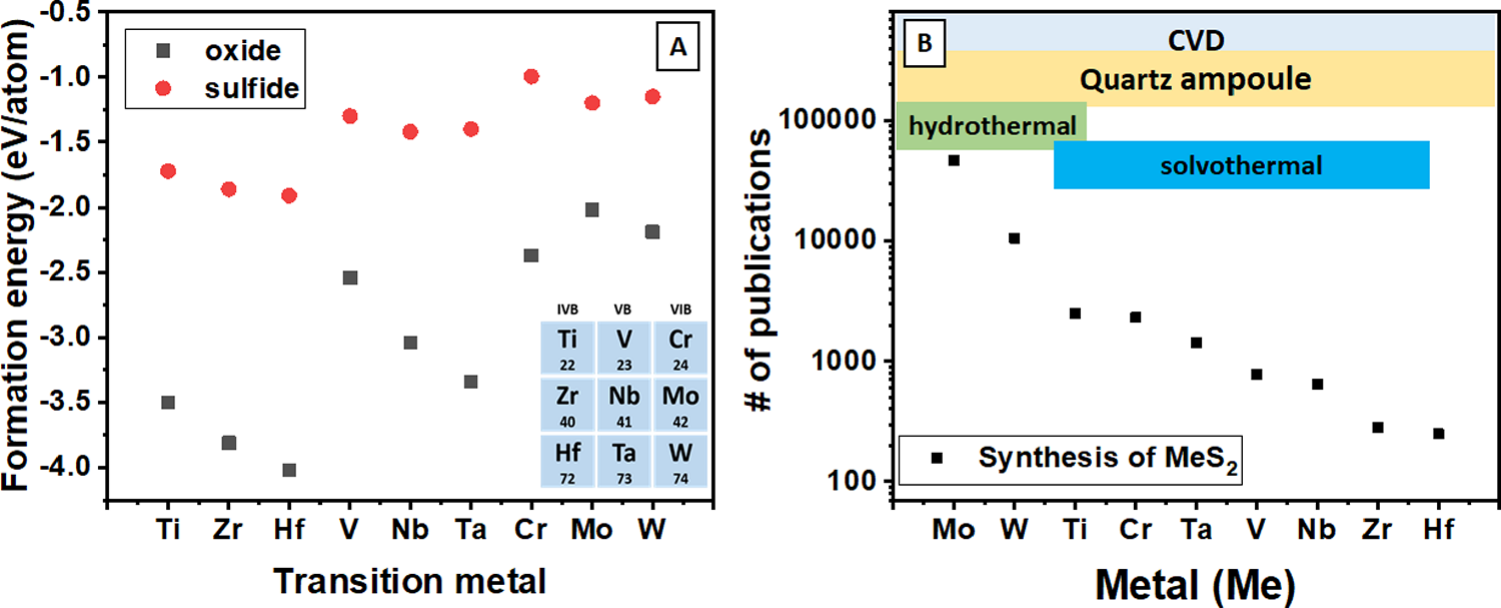
(A) Thermodynamic parameters of TM oxides and sulfides confined; (B) number of published papers (up to the end of 2023) on common TM sulfides, including preparation methods.

The synthesis of different transition metal sulfides was performed with utilization of oxides as a metal precursor and CS_2_ as a sulfur precursor. As was mentioned above, the tendency of TMDCs for oxidation can limit the applicability of some synthesis methods. An overview of literature data is presented in Figure 1. In particular, Figure 1A shows the comparison between the chemical structures of transition metal sulfides and oxides (most stable) and their formation energy. As is evident, some oxides are highly stable with a large sulfide–oxide formation energy gap, which significantly restricts the available preparation techniques and requires more sophisticated and demanding procedures. In particular, thermodynamic parameters in the case of Mo and W (and “lower” stability of corresponding oxides) allow their preparation using a “simple” hydrothermal approach. A similar gap and oxide stability in the case of Cr also allow utilization of this approach.^35^ A limited number of papers were found for Ti,^36,37^ but some are in contradiction, while others postulate the unsuitability of this simple approach in the case of Ti sulfide.^33^ The higher stability of the oxides (compared to sulfides) and related higher energy gap forces lead to the use of the solvothermal approach(es) for preparation of transition metal sulfides. This tendency is reflected in the decreasing number of papers on sulfide preparation in comparison to Mo (47,463 papers), W (10,519 papers), Ti (2486 papers), Ta (1431 papers), V (1600 papers), Nb (643 papers), Zr (281 papers), and Hf (250 papers). The alternative CVD or quartz ampoule-based approaches, insensitive toward oxygen, can be considered as universal ones, but they require significantly more sophisticated equipment and are less scalable (for industrial means). In particular, CVD can be used for the scalable preparation of TMDCs in a thin film form but not in the powder form.^38,39^

### Synthetic Approach

**Figure 2:**
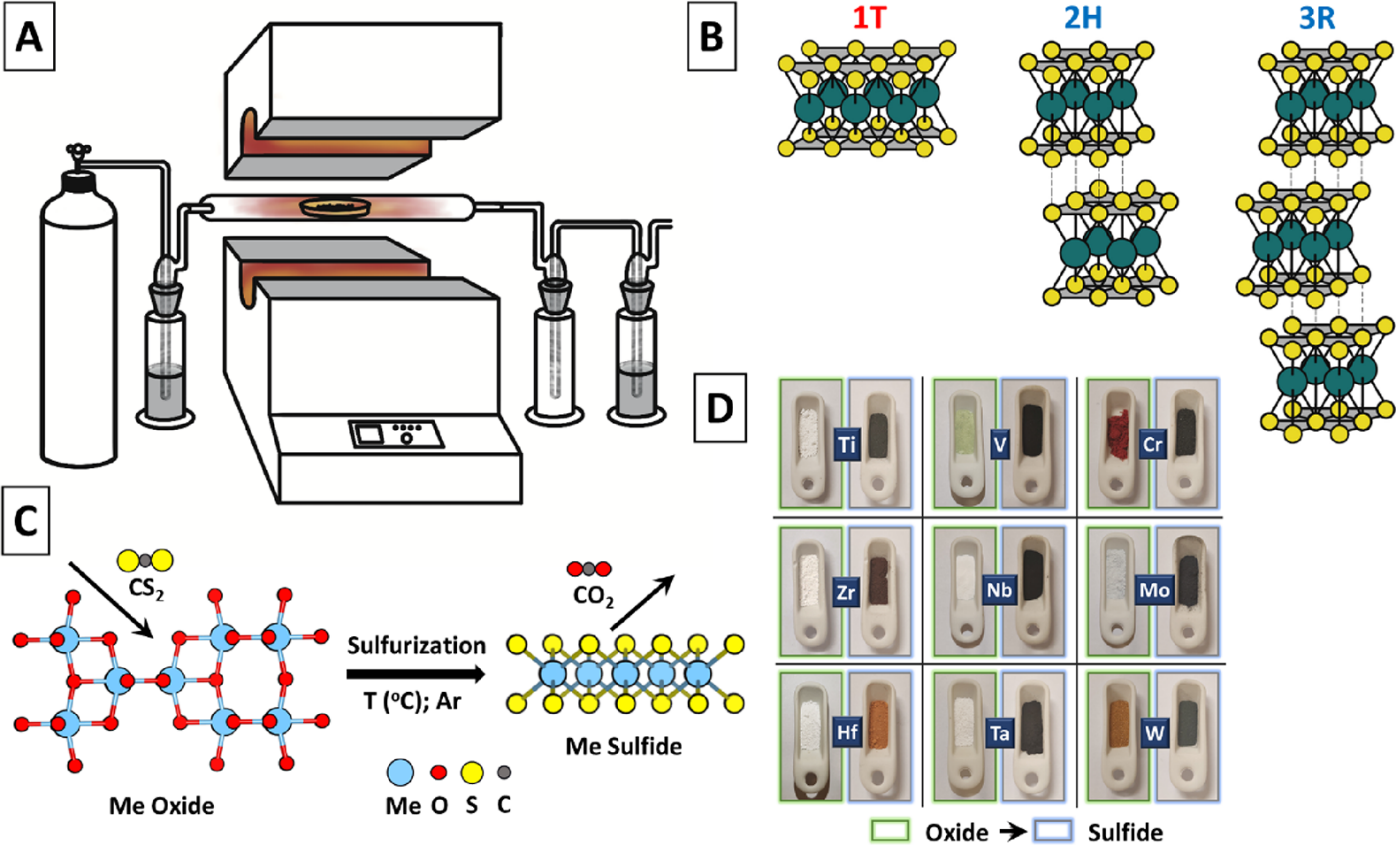
(A) Schematic representation of the experimental setup used for production of TM sulfides in CS_2_ flow under increased temperature; (B) schematic description of different TM sulfide crystalline structures, which can be produced using the proposed approach; (C) schematic and atomic structure of typical metal oxide sulfurization; (D) photos of the powders of metal oxide precursors and resulted sulfides.

In this work, our previous research was expanded to 9 transition metals of groups IV (Ti, Zr, and Hf), V (V, Nb, and Ta), and VI (Cr, Mo, and W). Briefly, synthesis was performed thermally in the tube furnace. Schematic representation of our experimental setup is given in Figure 2A. The quartz tube terminated with a nozzle from one side and a cap with a nozzle from the other side, which was used as a reactor. One nozzle was connected to the gas washing bottle filled with CS_2_, which played the role of a reductive and sulfurizing agent. A gas washing bottle with CS_2_ was itself connected to Ar, serving as a carrier gas. The other side, equipped with the cap with a nozzle, was connected to two gas washing bottles, first empty, as a trap for the backpressure flow of “cleaning” and the next one filled with a NaOH solution to remove residuals of unreacted CS_2_ (“cleaning” solution). A quartz boat loaded with the metal oxide, as a metal precursor, was immersed in the center of the reactor. The heating start was preceded by 30 min of flushing the system with argon. After the synthesis, the visible change that occurred in the sample color from initial white to black (Figure 2) gives the first manifestation of sulfide formation. Subsequently, the created samples were analyzed directly or subjected to liquid nitrogen-assisted exfoliation with the aim to produce 2D material.^40^ From the point of view of material analysis, the particular attention was focused on both production of TM sulfide and its crystalline structure. In these regards, it should be noted that the properties of TM sulfides are not determined by the elemental composition alone but also by their crystal structure. There are two ways by which chalcogenide atoms forming a slab can be arranged—by forming either a trigonal prismatic structure or octahedral structure, as is schematically shown in Figure 2B.^41^ An octahedral structure is commonly referred to as the 1T phase, while for a trigonal prismatic structure, the relative position of the layers can vary, resulting in various phases, with 2H and 3R as the most common representatives.^30,41,42^ Since the properties (conductivity, band gap, redox activity, etc.) of TM sulfides are strictly determined by their crystalline phase, the possibility to control the structure of the resulting material by a proper choice of the preparation route is of great importance.^21^

In Figure 2C, a schematic representation of sulfurization is presented. We estimate that CS_2_ takes the advantages of the crystal structure of the oxide and penetrates within the structure for further conversion.^43,44^ As a result, CO_2_ is estimated to be produced and leave the reactor; however, if the extent of CS_2_ is present, then products such as CO can be also estimated. Decomposition of CS_2_ to result in elemental sulfur and carbon can also take place during the process and can be observed on the cold side of the reactor.

### TM Sulfide Characterization

The quality of the prepared materials was investigated by X-ray diffraction (XRD), Raman spectroscopy, scanning electron microscopy (SEM), atomic force microscopy (AFM), and transmission electron microscopy (TEM), and the surface area was determined by Brunauer–Emmett–Teller (BET) analysis. To preserve the reasonable article length, the detailed results and corresponding discussion for each particular material are summarized in the Supporting Information (Figures S1–S40). The results are presented as a function of temperature used for sulfurization and analysis of the “final” material without metal oxide residues. The most significant results of XRD (crystalline structure of TM sulfides) and AFM (2D nature of resulted flakes) analyses are presented in Figures 3 and 4, respectively.

**Figure 3:**
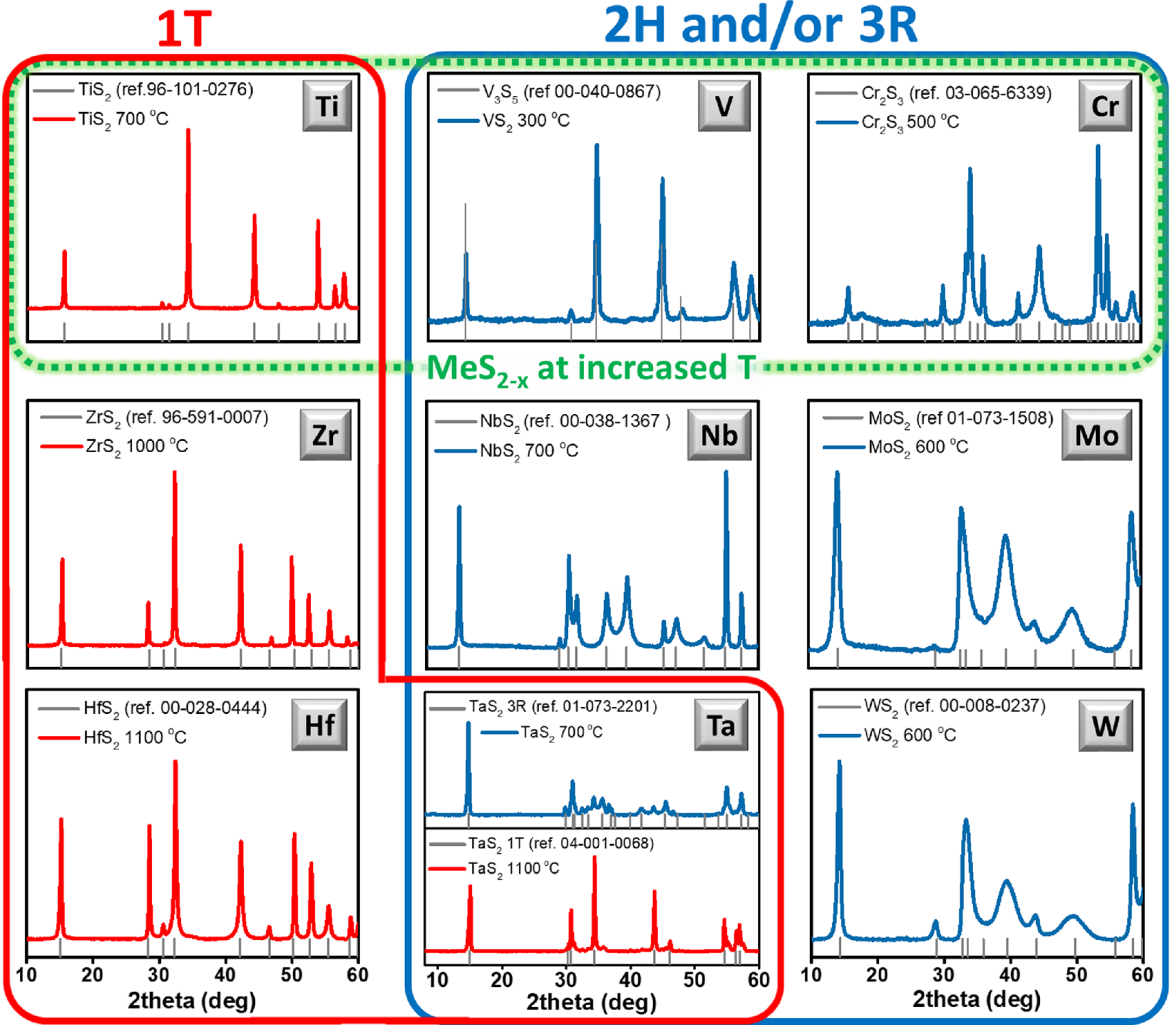
XRD patterns of pristine metal oxides and created sulfides confirmed the success and universality of the present approach.

**Figure 4:**
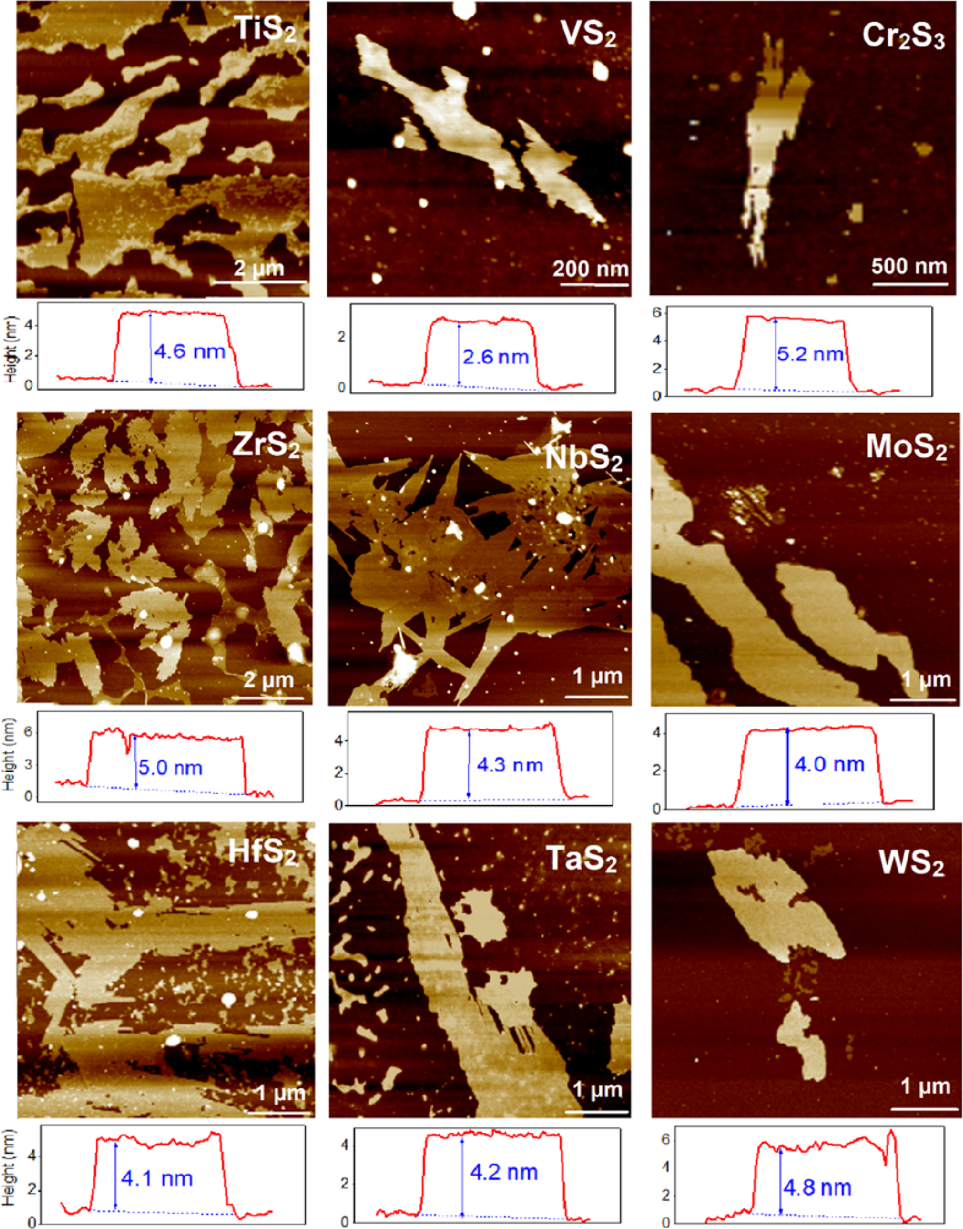
AFM images indicating the 2D nature of created TM sulfides with utilization of annealing in CS_2_ vapor and liquid nitrogen-assisted exfoliation.

Analysis of XRD spectra with regard to the transition metal position in the periodic table (as well as additional information from the Supporting Information) allows us to make the following conclusions: for Ti, Zr, and Hf, just one phase 1T is observed. Transition from Ti to Hf requires higher temperature for sulfurization, which is in agreement with initial oxide stability (Figure 1A). For V, Nb, and Ta, the 2H/3R phases are dominant and sulfurization proceeds at 300 and 700 °C. In the case of V, the deviation from MeS_2_ stoichiometry is observed—V_3_S_5_ is created at lower temperatures and subsequently transformed into V_3_S_4_ at higher temperature. For sulfurization of Ta oxide, a higher temperature is required (in agreement with Figure 1A) and, by further temperature increase, a shift from 3R to 1T phases can be achieved. Sulfurization of Cr, Mo, and W oxides proceeds at “moderate” temperature, with the dominant formation of the 2H phase of resulting TM sulfide. In addition, for the first row of elements in Figure 3 (Ti, V, and Cr), metal reduction with “loss” of sulfur atoms is observed (see the Supporting Information) as the synthesis temperature further increased, leading to the formation of MeS_2–*x*_ compounds. Noteworthily, XRD results are supported by Raman, XPS, and HRTEM/SAED results, presented and discussed in the Supporting Information.

The AFM scans of the exfoliated flakes of TM sulfides are presented in Figure 4 (the corresponding results of SEM are given in the Supporting Information). As is evident, 2D flakes were achieved for all synthesized TM sulfides. The flakes, however, differ in the lateral size significantly; tuning of the lateral size by external parameters (heating rate and sulfurization time) is out of the scope of this study and might be the topic of further investigation. All created flakes have similar thickness within the 2.6–5.2 nm range. Such a thickness indicates formation of few layers of TM sulfides. Taking into account the results of XRD and AFM (as well as additional characterizations methods, presented in the Supporting Information), we can conclude that the proposed method is suitable and highly universal for the synthesis of 2D transition metal sulfides. Additionally, Raman measurements indicate some changes in characteristic peak position and relative intensity after exfoliation (see Figure S41). Finally, all important results are summarized in Table 1, along with the “optimal” conditions required for the synthesis of materials and the corresponding phase. In addition, we also estimated the values of created material band gaps (using Tauc approximation), which were found to be in the 0.8–1.6 eV range (Figure S42).

**Table 1:** Summary of the Optimal Conditions of TM Sulfide Preparation Using Sulfurization in CS_2_ Vapor at Increased Temperature

**1T-TiS**_**2**_ = 600–900 °C	**2H-VS**_**2**_ = 250–300 °C	**2H-Cr**_**2**_**S**_**3**_ = 500–900 °C
**TiS**_**(2–*x*)**_ > 900 °C	**VS**_**(2–*x*)**_ > 300 °C	**Cr**_**3**_**S**_**4**_ > 900 °C
**1T-ZrS**_**2**_ ≥ 1000 °C	**3R-NbS**_**2**_ > 600 °C	**2H-MoS**_**2**_ ≥ 500 °C
**1T-HfS**_**2**_ ≥ 1100 °C	**3R-TaS**_**2**_ = 600–900 °C	**2H-WS**_**2**_ ≥ 500 °C
	**1T-TaS**_**2**_ > 900 °C	

It should be noted that the proposed method surpasses the more common solvothermal/hydrothermal approaches and is comparable with CVD or quartz ampoule approaches from the point of view of universality. However, our method is much more scalable than CVD and significantly faster than quartz ampoule methods, also requiring less complicated equipment, precursors, and preparation. Aside from the proof that there is room for optimization, e.g., by reactor engineering, we performed the synthesis of HfS_2_ (the most difficult material in our series to convert from oxide) for only 30 min and complete sulfurization was achieved if the powder mass was reduced to 100 mg and the powder was spread as a thin layer in the crucible; see the figure below with the XRD analysis of the obtained material (Figure S43).

## Conclusions

In this work, we propose an alternative approach for the synthesis of transition metal sulfides with utilization of metal oxides and CS_2_ as a sulfurization agent. The sulfide production was demonstrated on Ti, Zr, Hf, V, Nb, Ta, Cr, Mo, and W oxides. In all cases, the synthesis was successful after an optimization of experimental conditions. The main advantages of the proposed approach are simplicity, applicability for most (potentially all) transition metals, ability of subsequent creation of 2D flakes, availability of initial precursors, and simplicity of experimental equipment. Moreover, in some particular cases, we demonstrated the possibility to create materials with the different crystalline structures (1T, 2H, or 1R). We believe that the proposed approach may complement existing ones and overcome difficulties associated with the preparation of less stable sulfides, since our method can allow to obtain TMDCs despite their stability and thus boost the related research field. In such a way, it can also expand the research area of less popular sulfides (i.e., Nb, Zr, Hf, or Cr), where the existing barriers could be related to the complexity of their preparation.

## Experimental Part

Detailed description of the used materials and characterization methods is given in the Supporting Information. Briefly, the sulfurization of metal oxides was performed under the continuous flow of Ar, saturated by CS_2_ vapor in the tube furnace. Ar was used as a carrier gas. The samples were heated with 600 °C/h rate under Ar, then CS_2_ was added, and samples were kept at elevated temperature for 180 min. Subsequently, samples were cooled down in the spontaneous regime under the continuous CS_2_/Ar flow. The temperature range between 250 and 1100 °C was checked for most of materials.

## Supporting Information Available

Materials and equipment used for synthesis including corresponding conditions; detailed information on synthesis and exfoliation; SEM, XRD, Raman, XPS (survey and high-resolution details of selected peaks), TEM, and corresponding analysis of the obtained data for the sulfides of Ti, Zr, Hf, V, Nb, Ta, Cr, Mo, and W; BET data and Raman spectra for bulk and after exfoliation for selected phases (PDF)
