# Supplementary material for: Rapid and Universal Synthesis of 2D Transition Metal (Ti, Zr, Hf, V, Nb, Ta, Cr, Mo, and W) Sulfides through Oxide Sulfurization in CS2 Vapor
Source: Inorg Chem. 2024 Apr 24;63(18):8215–21. doi: 10.1021/acs.inorgchem.4c00475 (PMC11080058; doi:10.1021/acs.inorgchem.4c00475)
Supplement: Supplementary file 1 — ic4c00475_si_001.pdf [file ic4c00475_si_001.pdf]

Supporting Information For:

Rapid and Universal Synthesis of 2D Transition Metal (Ti, Zr, Hf, V, Nb, Ta, Cr, Mo, W) Sulfides through Oxide Sulfurization in CS2 Vapor

Vladislav Buravets[a], Frantisek Hosek[a], Vasiliy Burtsev[a], Elena Miliutina[a], Jaroslav Maixner[b], Ladislav Lapcak[b], Lucia Bajtosova[c], Miroslav Cieslar[c], Michal Procházka[d], Jan Minar[d], Zdenka Kolska[e], Vaclav Svorcik[a], Oleksiy Lyutakov[a,*]

[a] *Department of Solid State Engineering, University of Chemistry and Technology, 166 28 Prague, Czech Republic*

[b] *Central Laboratories, University of Chemistry and Technology, 166 28 Prague, Czech Republic*

[c] *Faculty of Mathematics and Physics, Charles University, 12116 Prague, Czech Republic*

[d] *New Technologies – Research Centre, University of West Bohemia, Univerzitní 8, Plzeň 30614, Czech Republic*

[e] *CENAB, Faculty of Science, J. E. Purkyne University, 40096 Usti nad Labem, Czech Republic*

**Correspondibng author*: oleksiy.lyutakov@vscht.cz

Experimental part

Materials

Titanium(IV) oxide, anatase, 99.8 % trace metals basis; zirconium(IV) oxide 99 % trace metal basis; hafnium(IV) oxide 99 %; vanadium(V) oxide (V_2O_5) > 98 %; niobium(V) oxide 99.99 % trace metal basis; tanatalum(V) oxide (Ta_2O_5) 99 % trace metal basis; chromium(VI) oxide 99.9 % trace metals basis; molybdenum(VI) oxide 99.97 % trace metals basis; tungsten(VI) oxide 99.9 % - were all in powder form, purchased from Sigma-Aldrich. Carbon Disulfide (CS_2, 99.9 %) were purchased from Sigma-Aldrich (Please note: CS_2 is flammable and toxic). Ar (6.0 grade) was provided by SIAD.

Characterization

X-ray diffraction (XRD) analysis was performed using PanAnalytical X´pert Pro, with Cu Kα 1.54 Å wavelength excitation. HighScore Plus software was used for crystallographic analysis. Materials were analysed using PDF-4 database.

Scanning electron microscopy (SEM) was performed using LYRA3 GMU by Tescan. Morphological analysis was performed by atomic force microscope (AFM) using Bruker Icon Dimension AFM. Transmission electron microscopy (TEM) including high resolution imaging (HRTEM) was performed on (TEM, 2200FS, Jeol Ltd, Japan) equipped with a FEG cathode operated at 200 kV of acceleration voltage and a Centurio Large Angle SDD-EDX detector.

For the X-ray photoelectron spectroscopy (XPS) samples were measured in ultra-high vacuum (UHV) chamber with base pressure ≤ 3×10-8 Pa using a hemispherical analyser Phoibos 150 (SPECS Surface Nano Analysis GmbH) with multichannel CMOS detector. The non-monochromatic X-ray source XR 50 was operated with Mg Kα line (1253.6 eV). The survey spectra were obtained by 3 scans with energetic step size of 0.5 eV, pass energy 50 eV and dwell time 0.096 ms. High resolution spectra of core levels were measured by 20-60 scans with energetic step size of 0.05 or 0.1 eV, pass energy 30 eV and dwell time 0.198 ms. Samples were dropcasted on Si substrate and mounted on Mo or Ti sample holder using conducting silver paste. For analysing the measured spectra KolXPD software was used. The peaks were fitted using Shirley or linear background and Voigt and Doniach-Sunjic functions. Investigation by Raman spectroscopy was performed with Thermo Scientific DXR Raman Microscope with a 532 nm laser excitation.

Synthesis and exfoliation

Synthesis setup was earlier tested by our group for the synthesis of TaS_2[1]. In this work our research was expanded to 9 transition metals of group IV (Ti, Zr, Hf), V (V, Nb, Ta) and VI (Cr, Mo, W). Temperature range was also extended and varied from 250 °C (when applicable) up to 1100 °C. Briefly, synthesis was performed thermally in the tube furnace. Quartz tube terminated with a nozzle from one side and cap with a nozzle from the other side, was used as a reactor. One nozzle was connected to the gas washing bottle filled with CS_2, which played the role of reductive and sulfurizing agent. Gas washing bottle with CS_2 was itself connected to Ar, which was used as a carrier gas. Other side, equipped with the cap with nozzle was connected to two gas washing bottles, one empty, as a trap for the backpressure flow of the "cleaning" and next one filled with NaOH solution to remove residuals of unreacted CS_2 ("cleaning" solution). Quartz boat loaded with the metal oxide, as a metal precursor, was immersed in the centre of the reactor, followed by 30 min of flushing the system by argon, prior to start heating. Heating rate was set to 600 °C/hour. Flow rate of argon was controlled by flow meter and set to 100 sccm. Once temperature of choice was reached, it was held for 3 hours, followed by the normal cooling to room temperature.

Exfoliation of the material was performed in liquid nitrogen. The technique was shown to work earlier for MoS_2[2–4]. For this procedure 50 mg of materials was sent into 50 ml centrifuge tube. Then 10 ml of liquid nitrogen was added to the tube and left to evaporate. Such cycles were repeated for 10 times, after which 30 ml of isopropanol was added and ultrasonicated for 1 hour.

Titanium sulfide

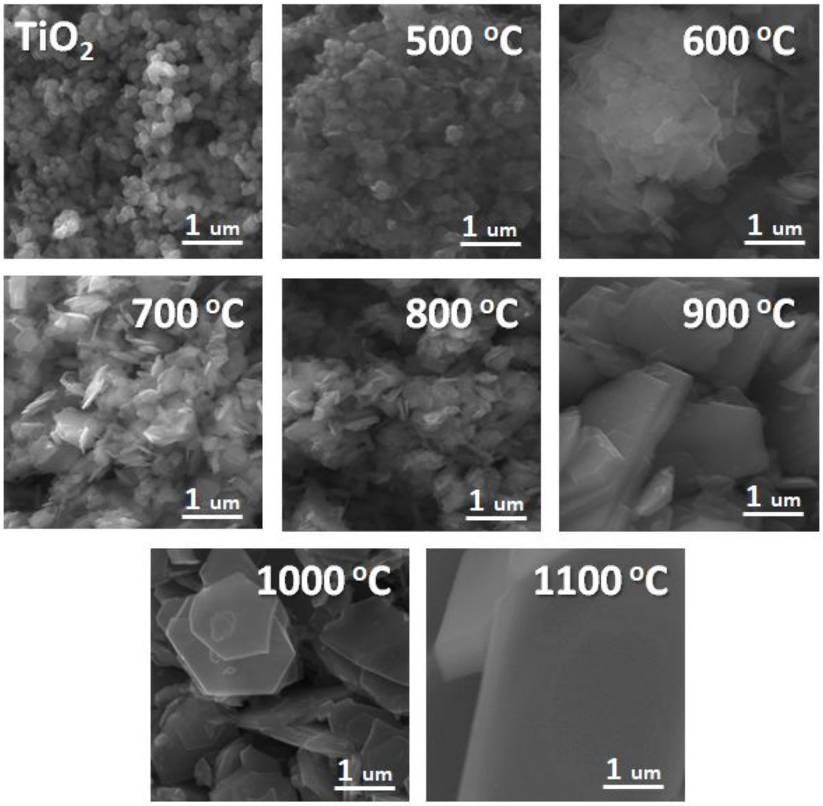

Figure S1. SEM images of Ti sulfide(s) prepared at different temperatures.

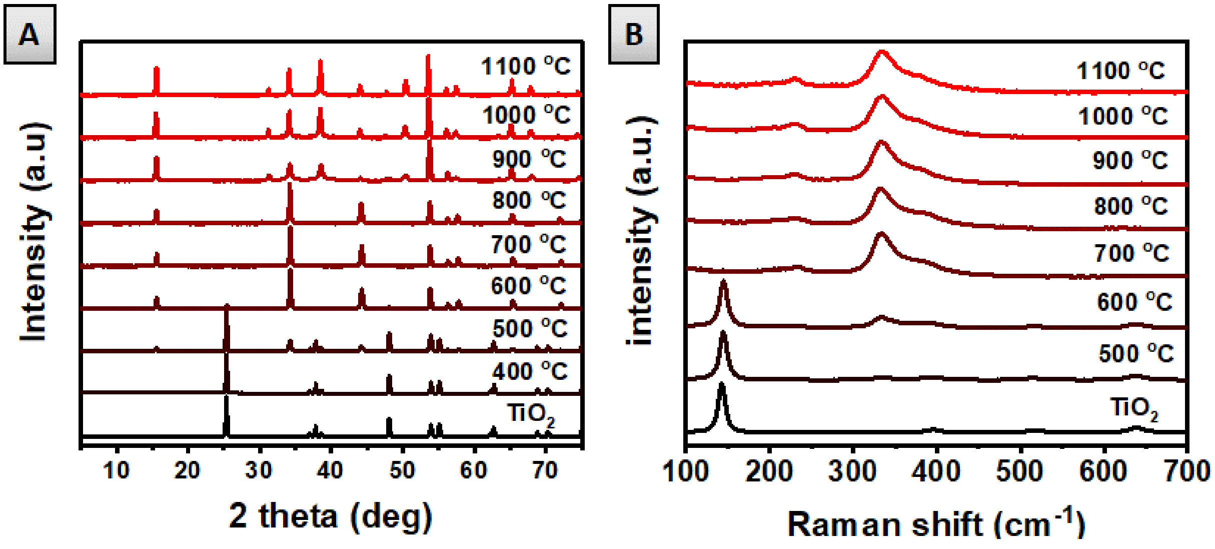

Figure S2. XRD patterns (A) and Raman spectra (B) of Ti sulfide(s) prepared at different temperatures.

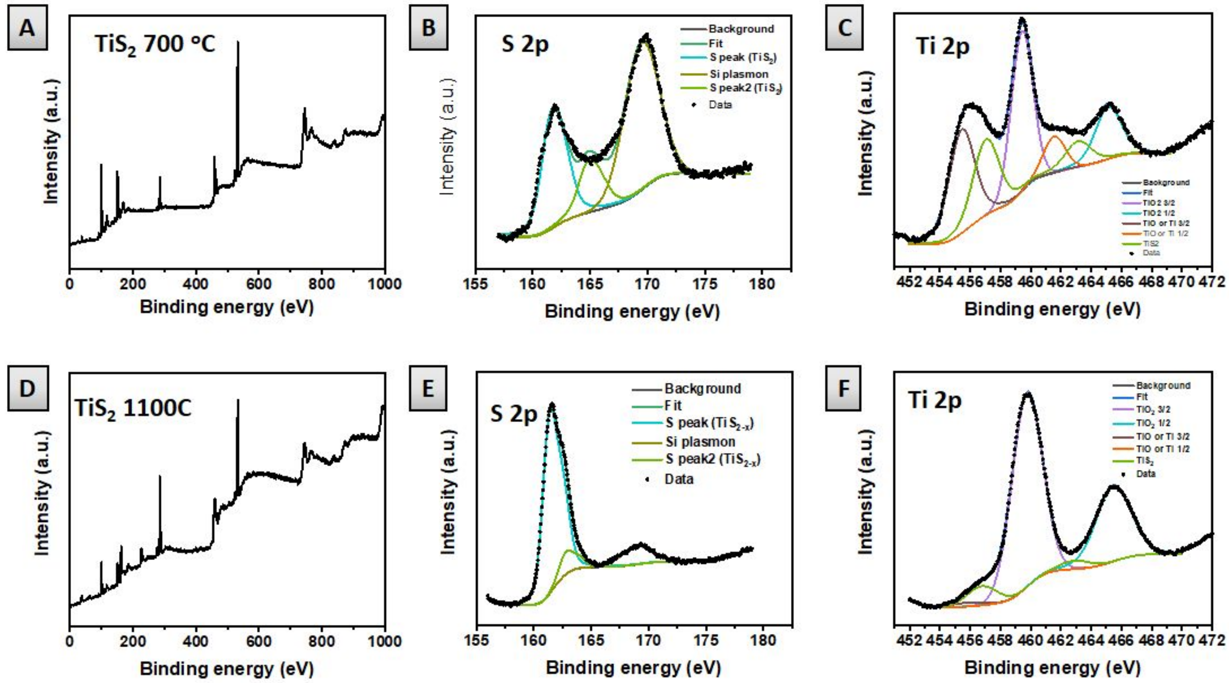

Figure S3. Survey XPS (A, D) spectra of Ti sulfide with details of deconvolution of characteristic S (B, E) and Ti (C, F) peaks, for TiS_2 synthesized at 700 °C (top) and 1100 °C (bottom).

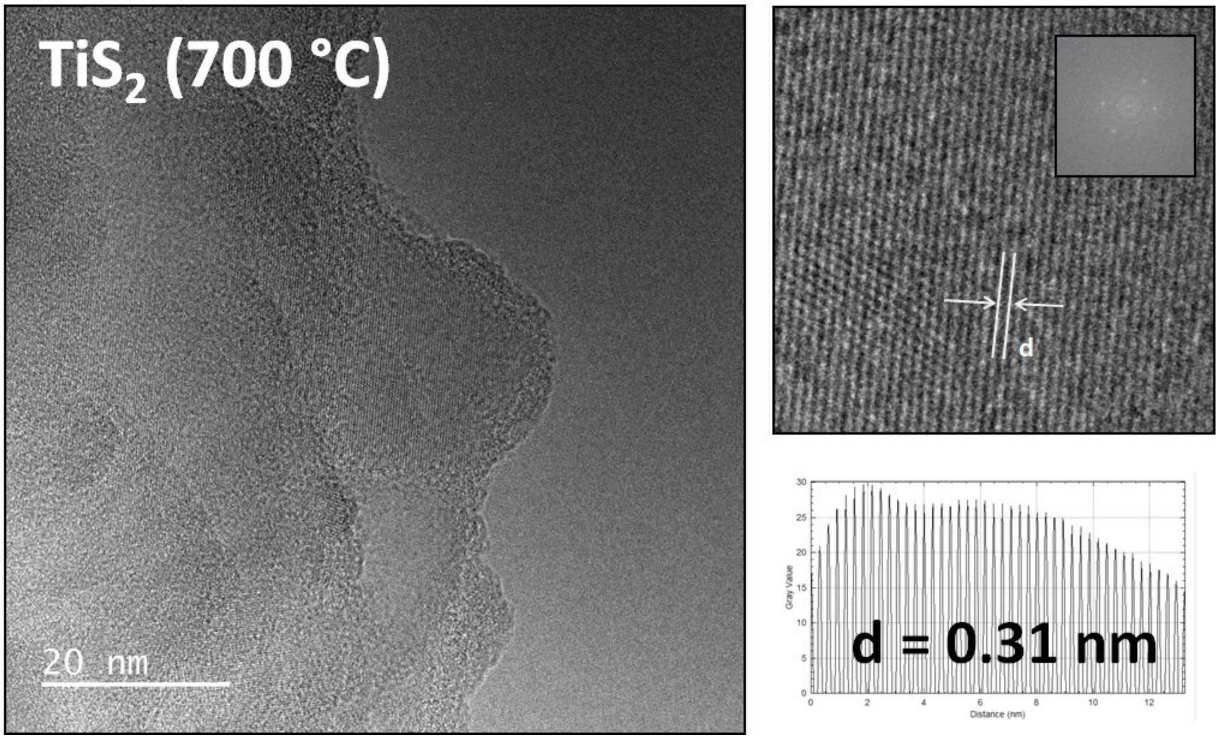

Figure S4. High resolution TEM image of Ti sulfide (prepared at 700 °C, measured after exfoliation).

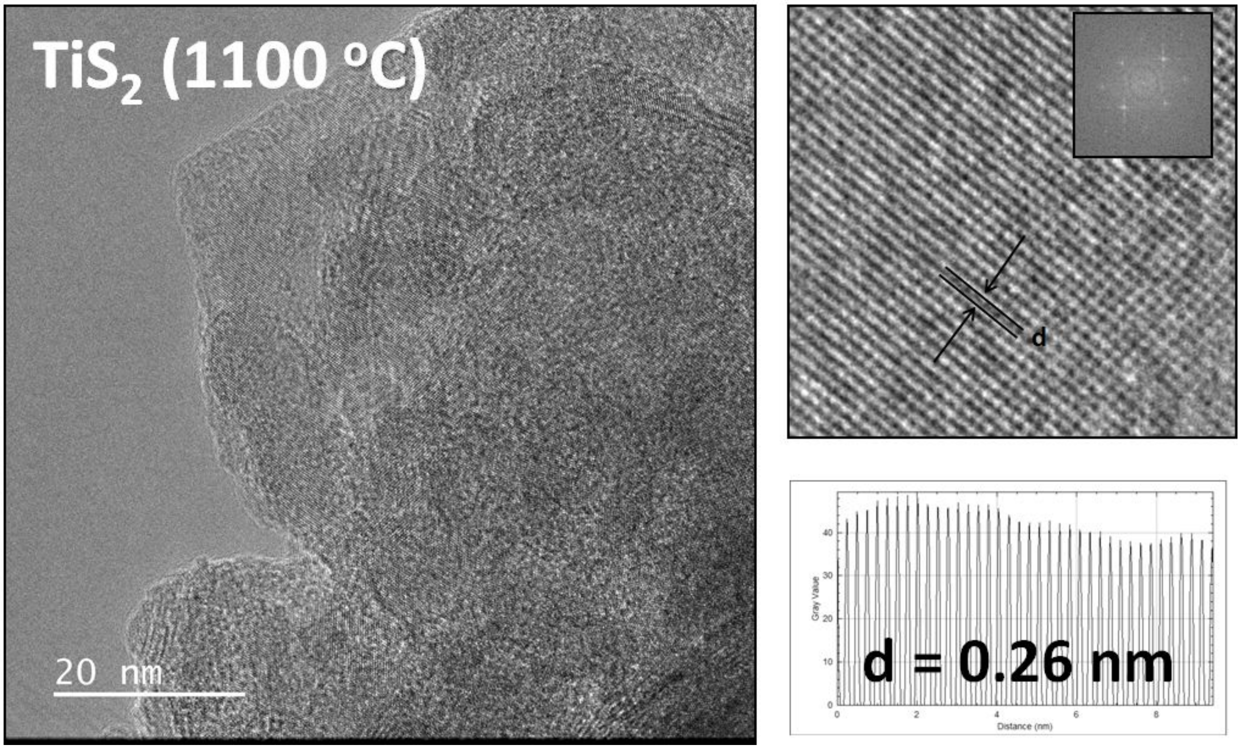

Figure S5. High resolution TEM image of Ti sulfide (prepared at 1 100 ^{o}C, measured after exfoliation).

Figures S1-S5 – related discussion

XRD. The obtained XRD data shows that the sulfurization of TiO_2 begins at ~600 ^{o}C, but 3 hours exposure is not enough for the entire conversion and oxide peaks are still prevailing. According to obtained XRD data entire sulfurization can be achieved at around 700 ^{o}C, with the phase corresponding to 1T-TiS_2 (ref. # 96-101-0276). When the sulfurization temperature overcomes 900 ^{o}C, the XRD patterns correspond to the $Ti_{2.45}S_4$ stoichiometry (ref. # 96-152-7889). This might be related to the reductive potential of CS_2 which causes the reduction of titanium from IV to lower oxidation state (in agreement with previous studies [5,6]).

***Raman* spectra** show that oxide peaks disappear around 700 ^{o}C and characteristic spectra for TiS_2 can are observed. Peaks at ~205, ~230, ~334 and ~382 cm^{-1} are in good agreement with previously observed ones, including shoulder peak (Sh) characteristic for TiS_2 [7–10]. In contrast to above XRD data, there is no significant change in the Raman spectra with increased temperature.

XPS. The high resolution XPS spectra of S 2p and Ti 2p are shown in Fig. S3. XPS analysis of obtained TiS_2 corresponds well with spectra observed in the literature[11–14]. Pairs of peaks observed for Ti 2p 3/2-1/2 correspond to TiO_2, TiO and TiS_2[14] (partial oxidation of titanium sulfide proceeds

immediately after its contact with air). Presence of the oxide peaks is unavoidable after even few hours of storing the material at the ambient conditions[12,15]. Observed shift of the Ti 2p peak toward lower binding energy as the temperature is increased suggests that Ti is in the more reduced state[11].

HRTEM. High resolution TEM image of TiS_2 is presented in Fig. S5. Analyzed interatomic distance was determined to be 0.31 nm and 0.26 nm for material synthesized at 700 and 1100 ^{o}C respectively and it is in well agreement with literature[7,16].

Zirconium sulfide

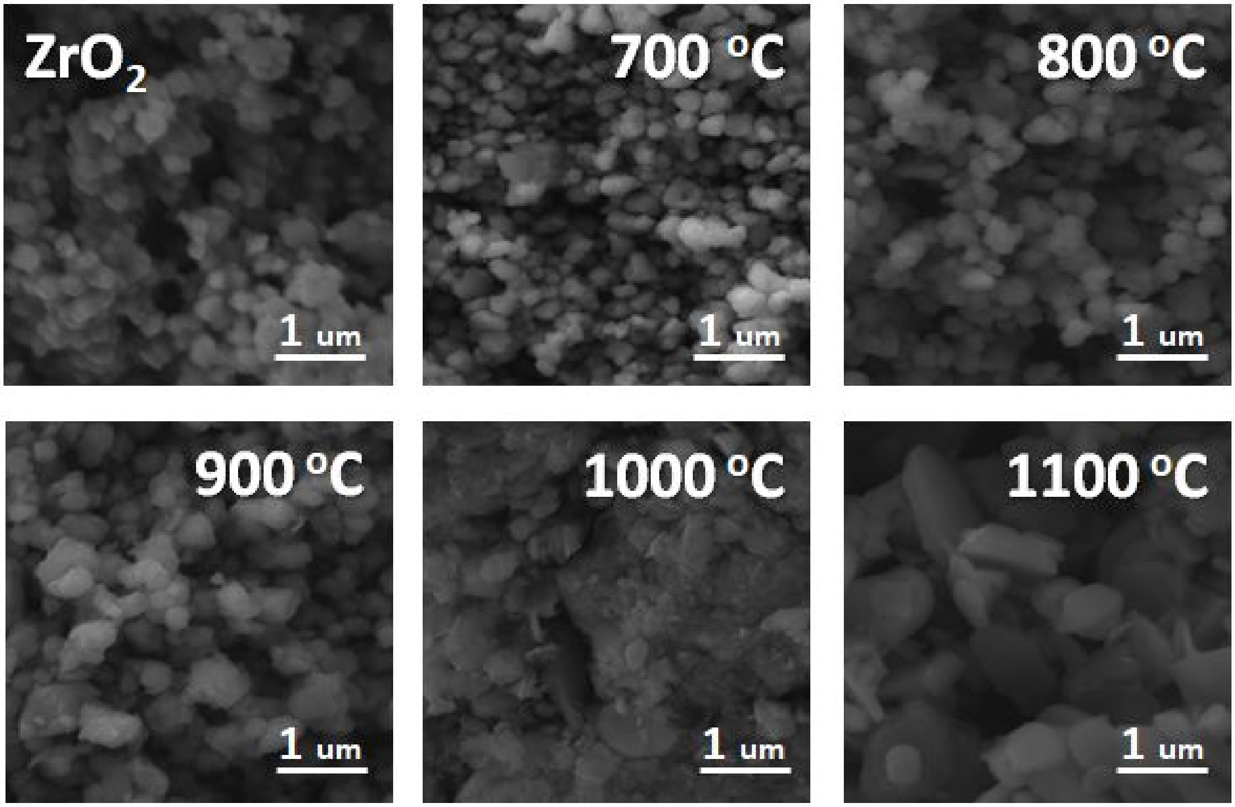

Figure S6. SEM images of Zr sulfide(s) prepared at different temperatures.

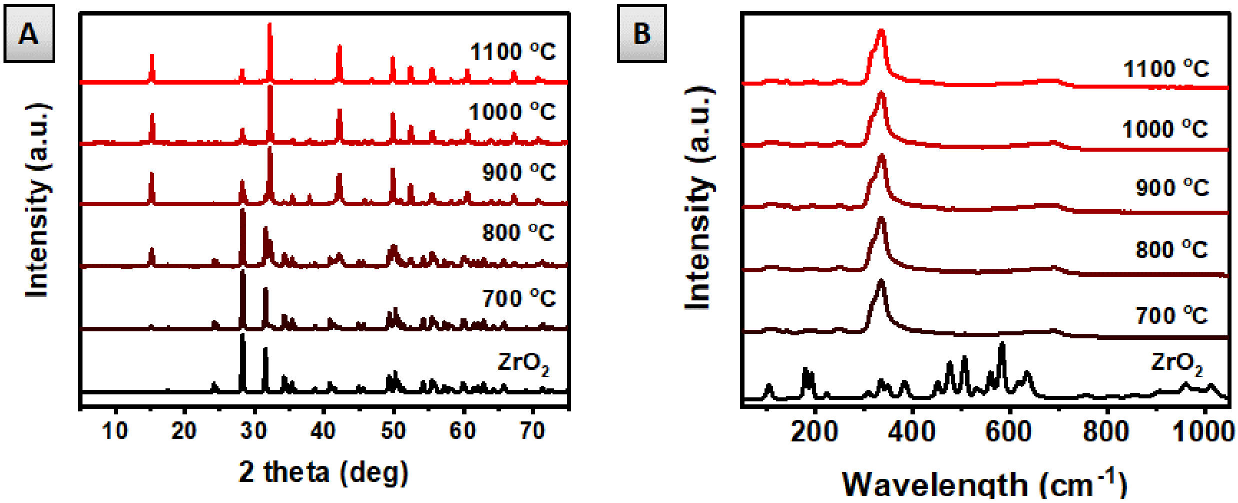

Figure S7. XRD patterns (A) and Raman spectra (B) of Zr sulfide(s) prepared at different temperatures.

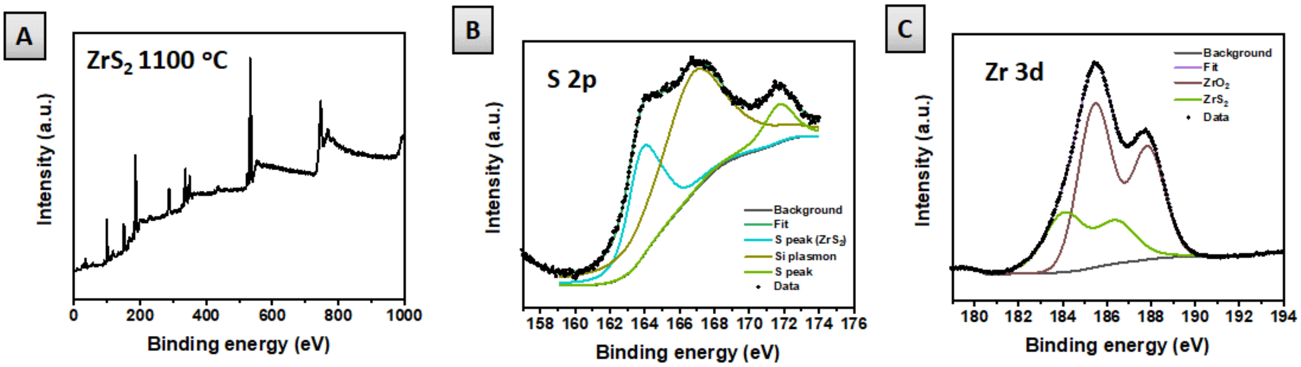

Figure S8. Survey XPS spectra of Zr sulfide (A) and deconvolution of characteristic S (B) and Zr (C) peaks details.

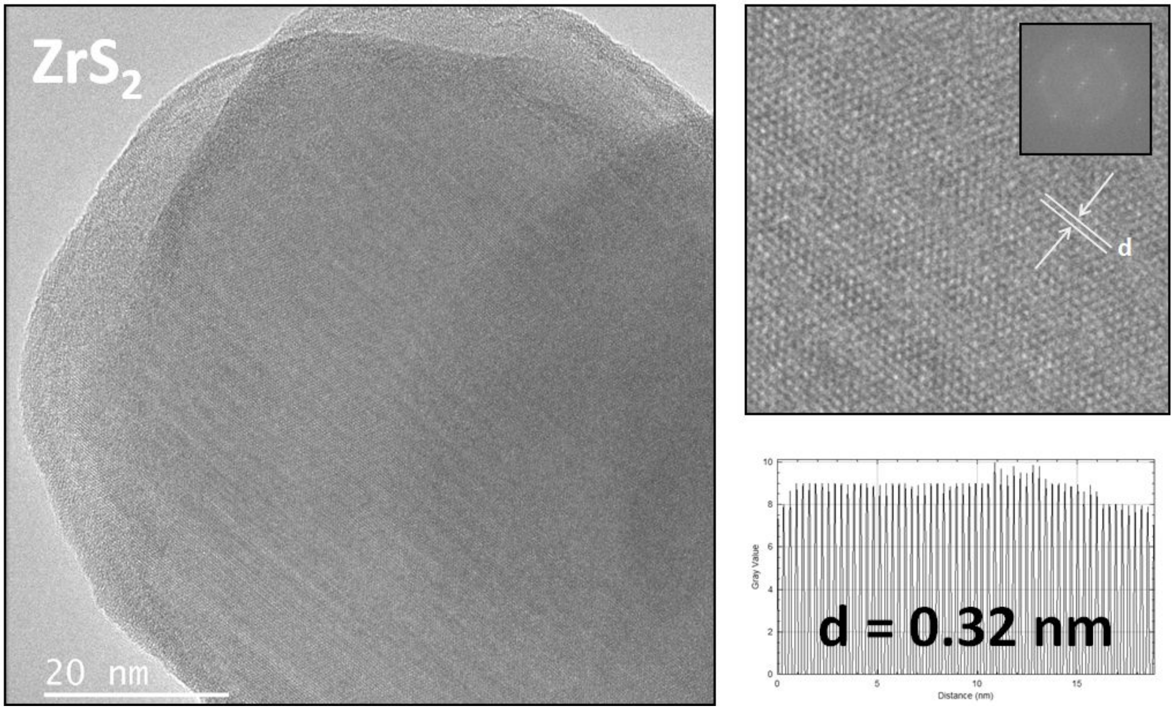

Figure S9. High resolution TEM image of Zr sulfide (after exfoliation).

Figures S6-S9 – related discussion

XRD. First changes in the XRD spectra of ZrO_2 appear at temperature around 700 ^oC (Fig. S7). Nevertheless, only at temperatures above 1000 ^oC, the oxide peaks completely disappear. Observed spectra of sulfurized zirconium include peaks at 15.21, 28.11, 32.11, and 42.11 corresponding to the diffraction of the (001), (100), (101), and (102) planes, respectively, corresponding to 1T phase of ZrS_2 (ref. # 96-591-0007) [17].

Raman spectroscopy reveals ZrS_2 characteristic peaks at ~250 and ~334 cm^{-1} (E_g and A_g modes, respectively)[18–20] for all synthesis temperatures above 700 ^{o}C. Noteworthy, Raman shift signals are apparently much stronger for ZrS_2 than for the oxide, as according to XRD data only small amount of ZrO_2 is converted to sulfide at 700 ^{o}C. Broad A_g mode and presence of extra peak around 316 cm^{-1} (intensity of which is increased with temperature) was ascribed in more details elsewhere [19,21,22].

XPS. High resolution XPS spectra of Zr 3d and S 2p are shown in Fig. S8. The obtained results correlate well with the data reported previously in literature[14,23–25]. Zr $3d_{5/2}$ and Zr $3d_{3/2}$ peaks related to ZrS_2 and ZrO_2 are observed and can overlap. This is supported by the presence of 2 components of S 2p peaks, attributed to intrinsic sulfur and sulfate ions[23,25]. Presence of oxide-related peaks can be attributed to a post-preparative oxidation, during the samples contact with air.

HRTEM. High resolution TEM image of ZrS_2 is presented in Fig. S9. Interatomic distance was determined to be 0.32 nm.

Hafnium sulfide

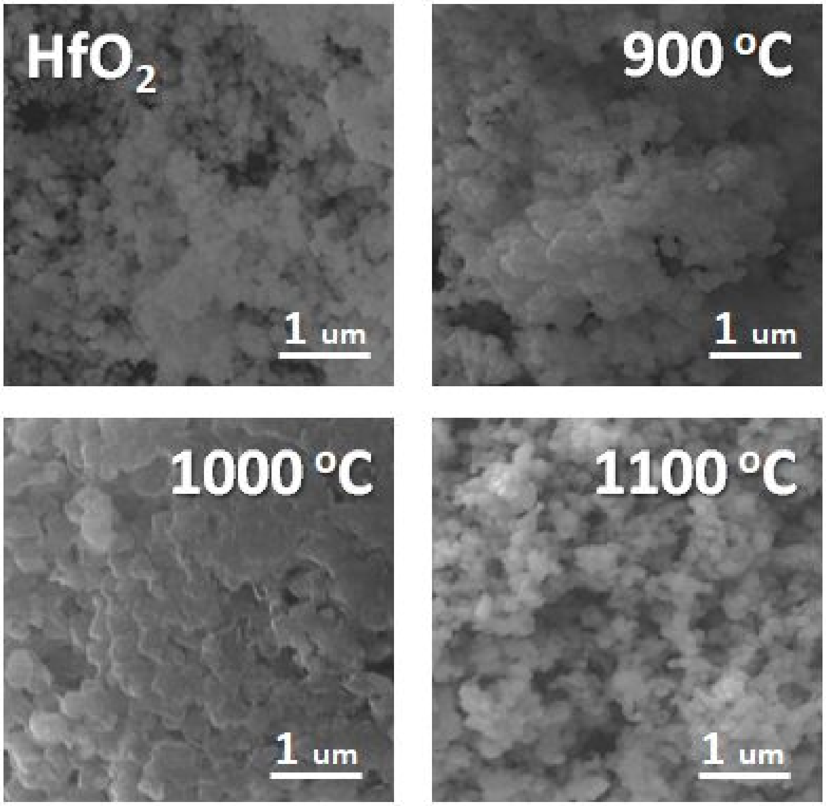

Figure S10. SEM images of Hf sulfide prepared at different temperatures.

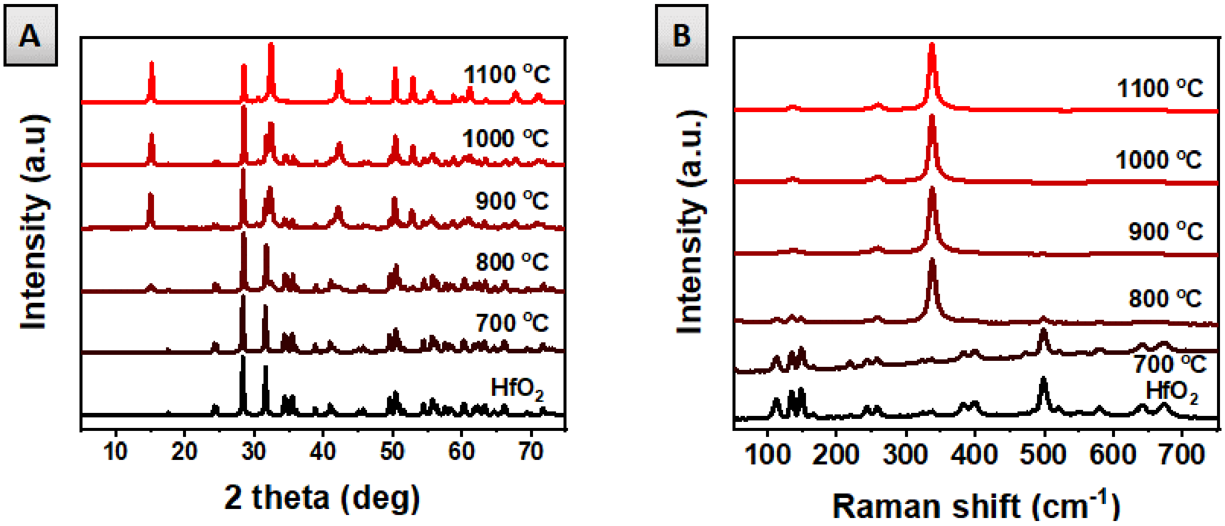

Figure S11. XRD patterns (A) and Raman spectra (B) of Hf sulfide(s) prepared at different temperatures

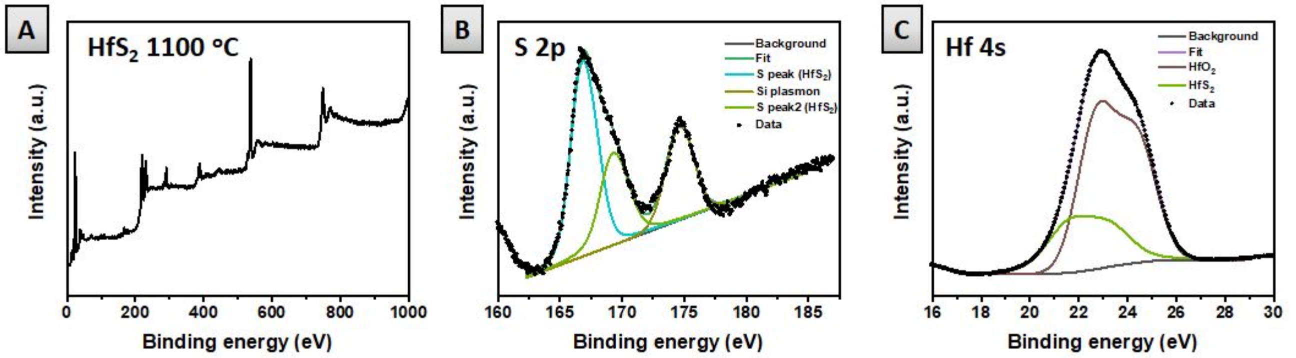

Figure S12. Survey XPS spectra (A) of Hf sulfide and deconvolution of characteristic S (B) and Hf (C) peaks details.

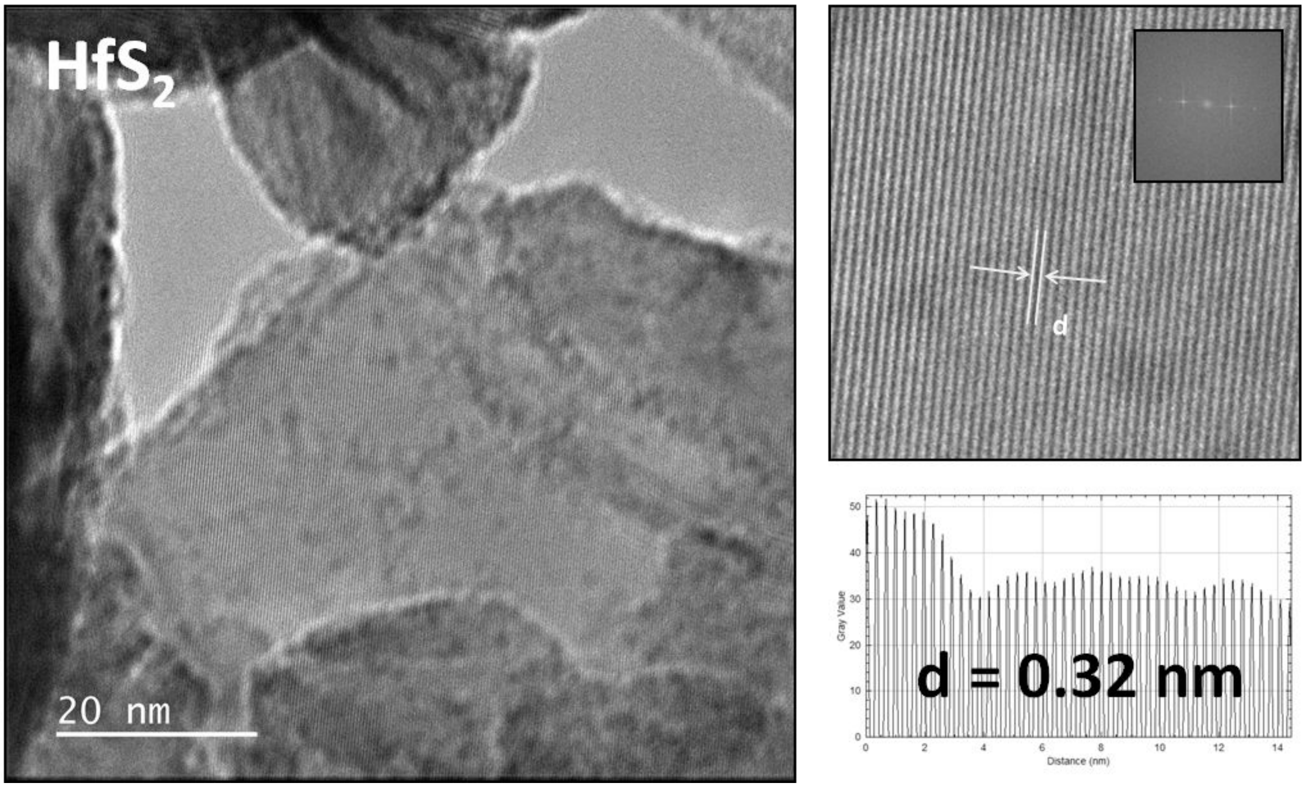

Figure S13. High resolution TEM image of Hf sulfide (after exfoliation, sulfurization was performed at 1100 ^{o}C).

Figures S10-S13 – related discussion

XRD. Sulfurization of HfO_2 does not occur below the temperature 800 ^{o}C, but only trace amounts of HfS_2 can be observed. Entire sulfurization is obtained only at the temperature about 1100 ^{o}C, with no residues of oxide peaks, corresponding to 1T-HfS_2 (ref. # 00-028-0444).

Raman spectroscopy (Fig. S11) shows 2 characteristic peaks around 343 and 257 cm^{-1} corresponding to A_{1g} and E_g modes [26,27]. Additional peak at 137 cm^{-1} was detected which is known for HfS_2 however it's nature is still debated [28]. Similarly to ZrS_2 patterns, those corresponding to HfS_2 have stronger intensity than that of oxide, thus, obscuring presence of oxide at temperatures below 1100 ^{o}C (otherwise detected by XRD).

XPS. High resolution XPS spectra of Hf 4f and S 2p are shown in Fig. S12. The obtained results correlate well with the data reported previously in literature [14,26,29]. HfS_2 as well as other sulfides are prone to oxidation and it was shown that in few hours at ambient conditions surface oxides are formed resulting in peek pairs observed in Hf 4f spectra [14,26,29]. These results are supported by presence of 2 peaks of S 2p in the high resolution spectra [14].

HRTEM. High resolution TEM image of HfS_2 is presented in Fig. S13. Analysed interatomic distance was determined to be 0.32 nm and is in good agreement with the literature[30]

Vanadium sulfide

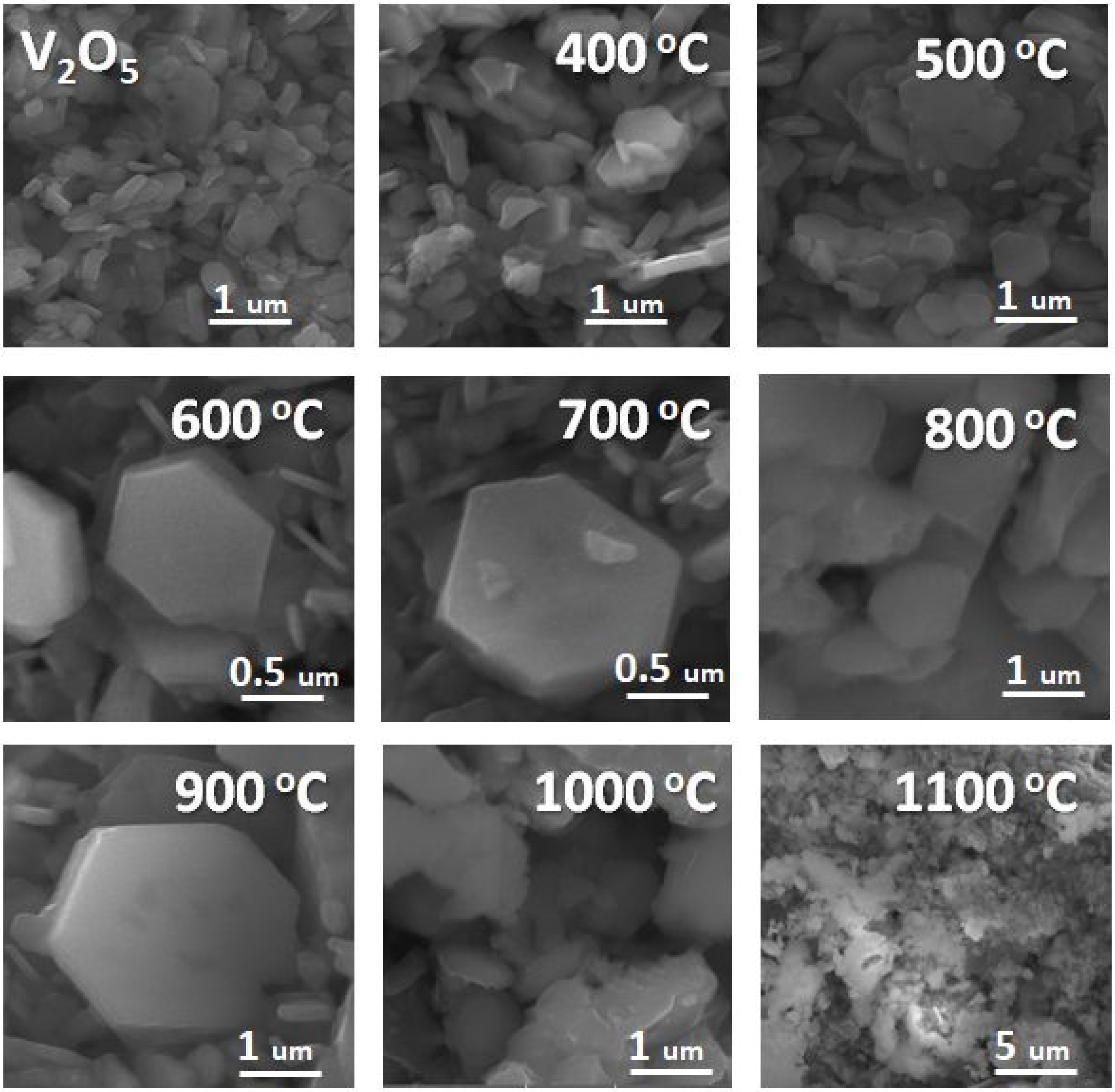

Figure S14. SEM images of V sulfide(s) prepared at different temperatures.

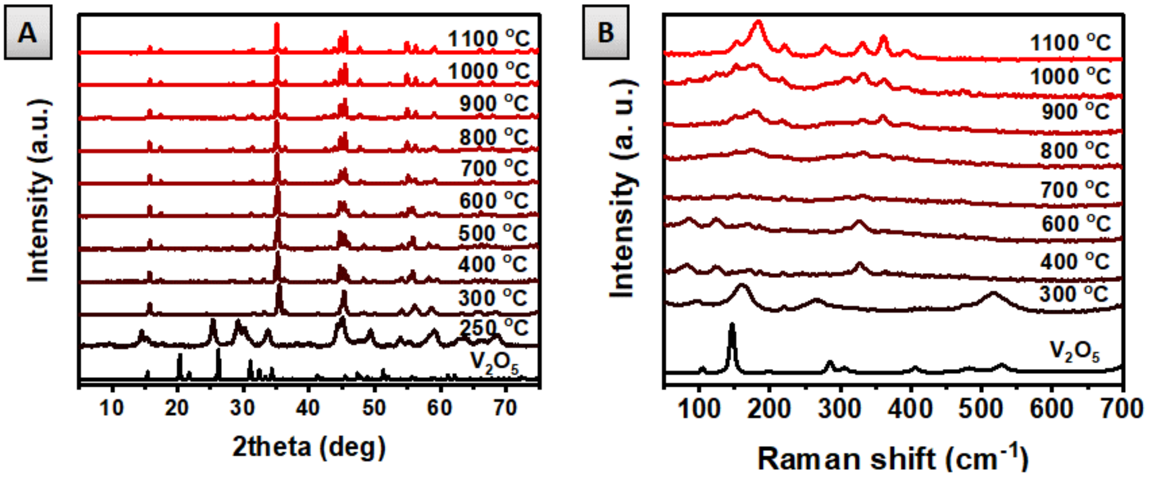

Figure S15. XRD patterns (A) and Raman spectra (B) of V sulfide(s) prepared at different temperatures.

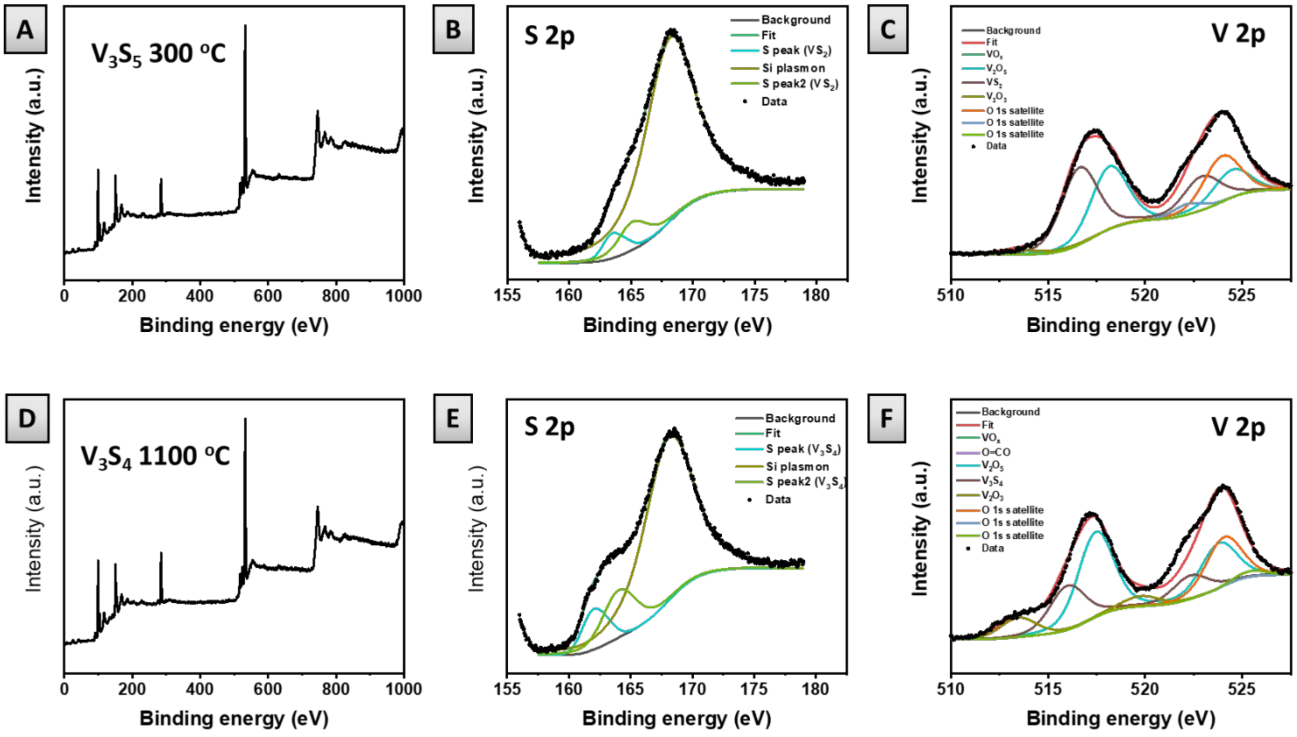

Figure S16. Survey XPS spectra of V sulfides (A, D) and deconvolution of characteristic S (B, E) and V (C, F) peaks details of materials prepared at 300 °C (top) and 1100 °C (bottom).

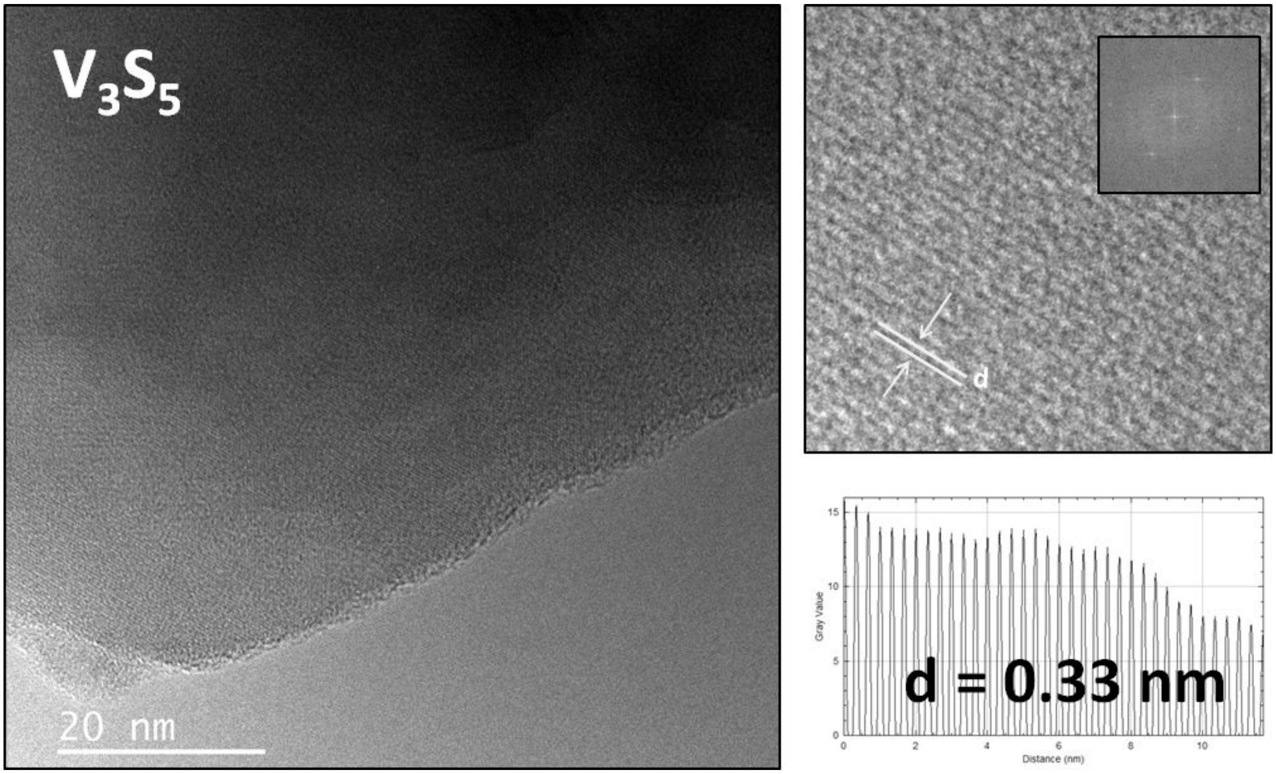

Figure S17. High resolution TEM image of V sulfide (after exfoliation, prepared at 300 ^{o}C).

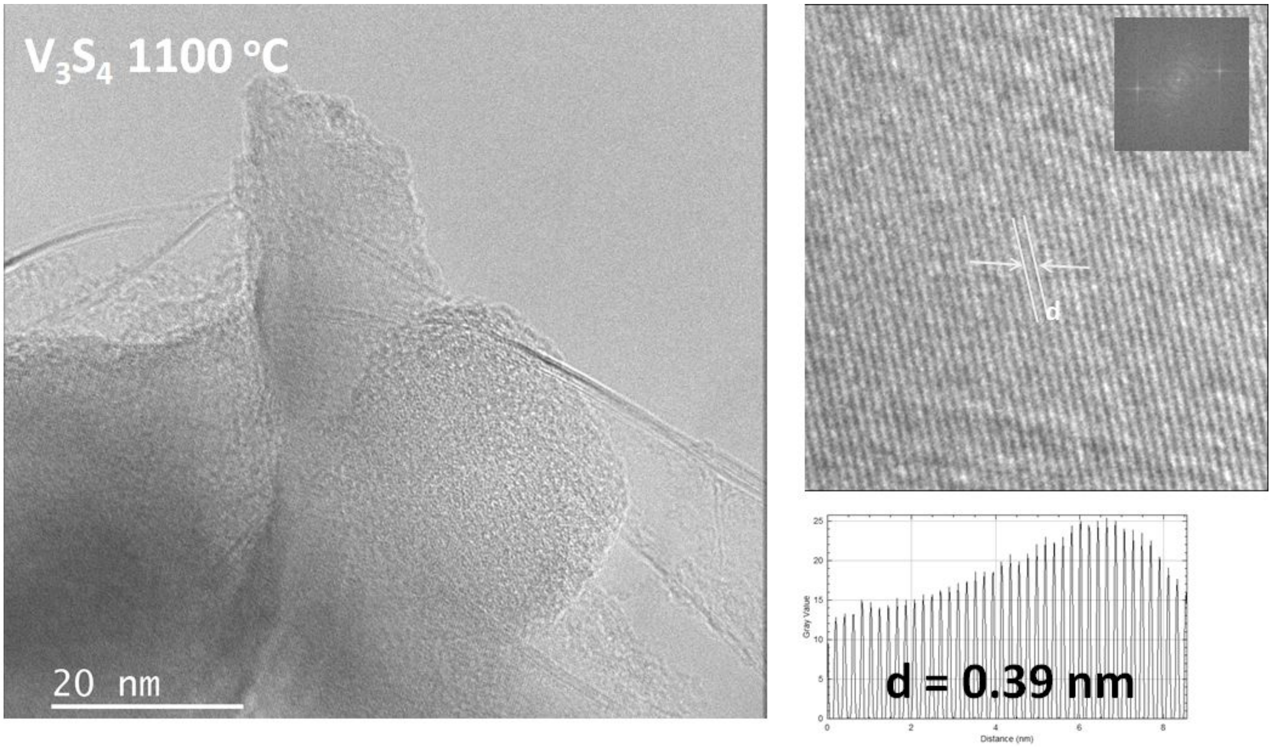

Figure S18. High resolution TEM image of V sulfide (after exfoliation, prepared at 1100 ^{o}C).

Figures S14-S18 – related discussion

XRD. Vanadium sulfide has a non-trivial composition of VS_{2-x} with multiple variation of x, known for decades[31]. Reaction of V_2O_5 with CS_2 at elevated temperatures results in reduction to VO_2 at around 250 °C and V_2O_3 peaks (ref. # 96-901-6536) are present at 250 °C. However, sulfide peaks are prevailing at slightly increased temperature (300 °C), corresponding to V_3S_5 (ref. # 00-040-0867). Further temperature increase results in better crystallinity, but it also leads to more reduced vanadium state with lower ratio of sulfur to vanadium (ref. # 96-153-7895). This is supported by the appearance of the peak at 17.5 degrees and its further increase with temperature. This peak is characteristic for V_3S_4 and should not be present for VS_2. Finally, sulfurization of vanadium results in the pattern, corresponding well with Me_3S_4 structure, with Me standing for other transition metals.

Raman. Spectrum of Vanadium sulfide, which is prone for oxidation, can be rather controversial. The oxide spectra can be easily misinterpreted as those corresponding to VS_{2-x}. Thus, a comparison with the initial oxide spectra is crucial as reference. Also, the low power is suggested, to avoid appearance of the oxide peaks at 145, 198, 283, 407, 526, 701 and 995 cm^{-1} (Fig. S15), commonly presented in the literature.

Raman spectra of the sample synthesized below 400 °C are significantly different from that of V_2O_5. The oxide peaks disappear, while new peaks appear at 160, 218, 265, 515 and 701 cm^{-1}. Raman spectra of VS_{2-x} synthesized in the temperature range from 400- 600 °C correspond well to the spectra reported for VS_{2-x} [32–34], with the characteristic peaks at 325, 185, 168, 123 and 85 cm^{-1}. As the temperature is increased above 700 °C, phase transition takes place and the peaks of VS_{2-x} disappear and new peaks become prominent at 151, 183, 221, 278, 330, 359 and 392 cm^{-1}. This is in good agreement with the reported synthesis of VS_{2-x} in the H phase, which take place at temperatures above 700 °C [35,36].

XPS. High resolution XPS spectra of V 2p and S 2p are shown in Fig. S16. XPS spectra reveals presence of both V and S in the created material. Observed peaks of V and S correspond well with vanadium sulfide reported in the literature[37,38]. Sulfur peaks (S 2p) at ~162.1 and 164.1 are attributed to the oxidation state of sulfur - S^{2-} [38]. Shift of the V 2p peak towards lower binding energy with temperature increase suggests the more reduced state of vanadium in the obtained material. This is rather characteristic for V_3S_4 since vanadium is in lower oxidation state [39–41]. Presence of oxide-related peaks can be attributed to a post-preparative oxidation, during the samples contact with air.

HRTEM. High resolution TEM image of VS_2 is presented in Fig. S17. Interatomic distance was determined to be 0.33 nm. Sulfurization at higher temperature leads to creation of VS_{2-x} and the interatomic distance increases to 0.39 nm (Fig. S18).

Niobium sulfide

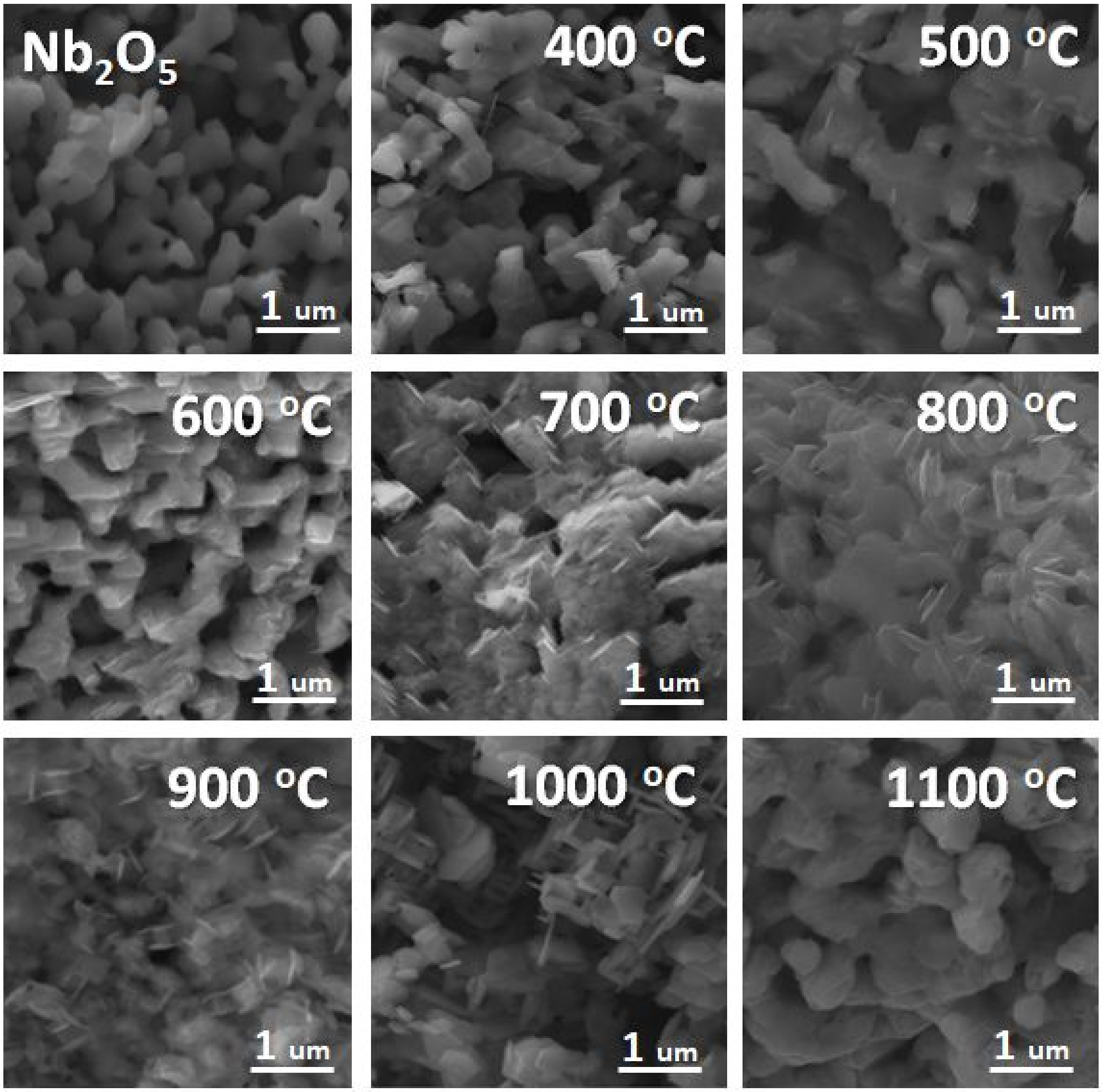

Figure S19. SEM images of Nb sulfide(s) prepared at different temperatures.

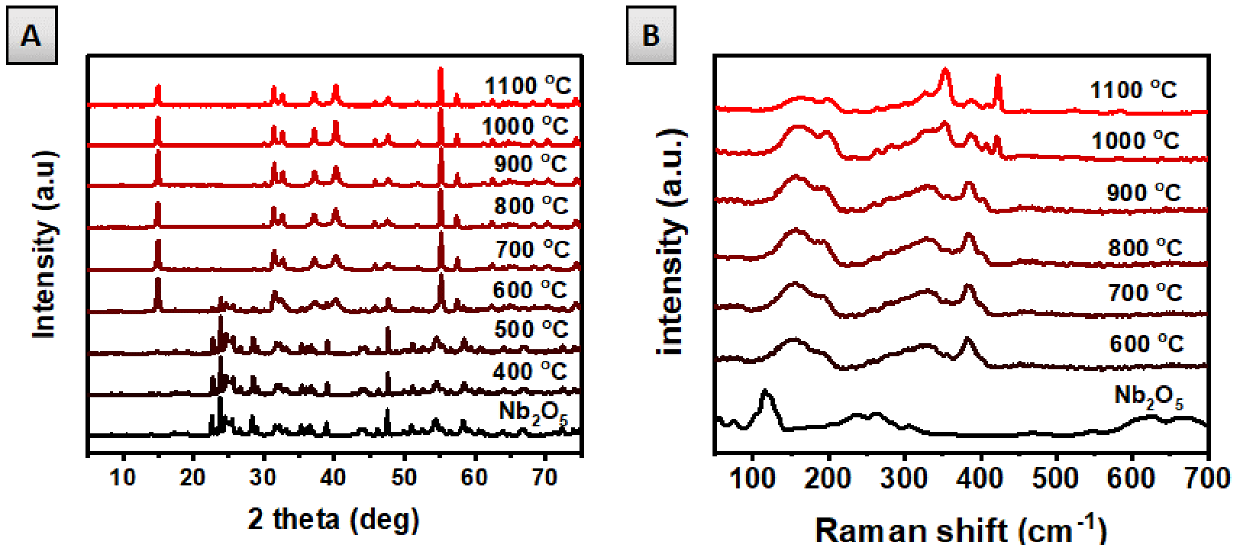

Figure S20. XRD patterns (A) and Raman spectra (B) spectra of Nb sulfide(s) prepared at different temperatures.

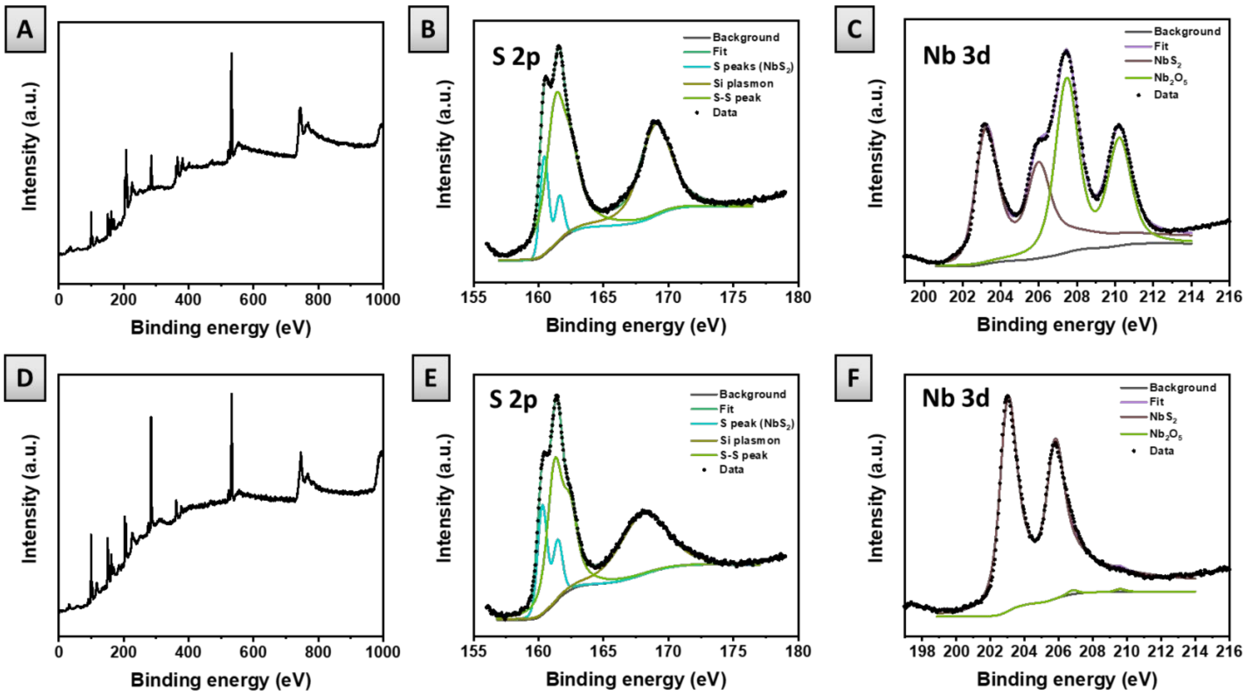

Figure S21. Survey XPS spectra of Nb sulfides (A, D) and deconvolution of characteristic S (B, E) and Nb (C, F) peaks details for materials synthesized at 700 °C (top) and 1000 °C (bottom).

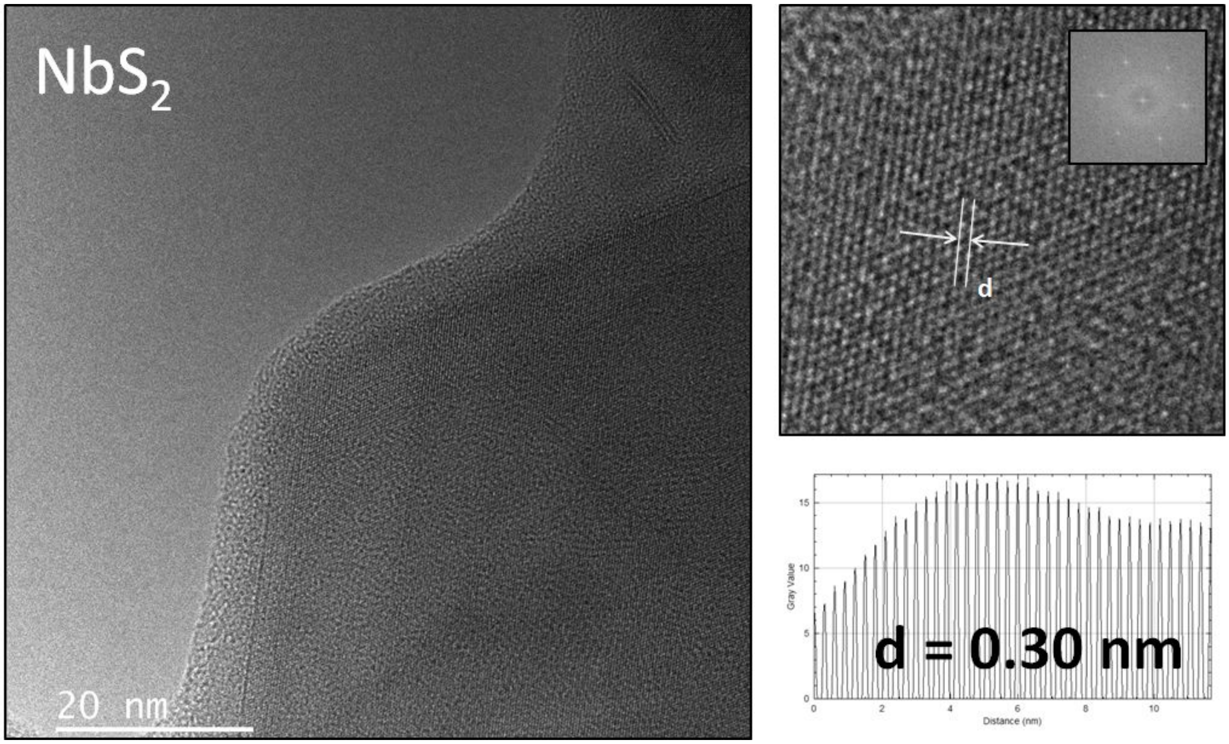

Figure S22. High resolution TEM image of Nb sulfide (after exfoliation, prepared at 700 °C).

Figures S19-S22 – related discussion

XRD. For comparison – if synthesis is performed from metal and with sulfur as the sulfurizing agent, the peaks of NbS_2 appear only at around 850 °C together with oxide peaks, which do not disappear entirely even at 1050 °C[42]. Absence of the peak around 35 degrees suggests that the phase is 3R[43]. Broader peak can be related to 2H phase, while sharp peaks are characteristic for the 3R phase[44,45]. Also, larger lattice parameter "c" is estimated for 2H phase than for 3R one, which can be also observed in the resulting XRD pattern. With temperature increase some small shift of the peak from 14.92 to 14.95 can be observed, also pointing on the transition to 3R phase. Above mentioned results correspond well with those reported for 3R polytype which is thermodynamically stable phase at room temperature, with good agreement with ref. # 04-004-7343 [46,47].

Raman. Phase recognition between 3R and 2H was found to be possible for NbS_2 [44]. Thus, appearance of the peak at 402 cm^{-1} suggests transformation to 3R phase, however it can also be related to the thickness of the 3R phase[48]. Presence of the so called "defect" mode between 100-200 cm^{-1} is reported[49] to be unique for 3R-NbS_2 and can be observed on the obtained Raman spectra for all 3 samples. Obtained spectra also correlate well with the reported data in literature[48–51]. Thickness increase was reported to affect E_1 and E_2 (between 300 and 350 cm^{-1}) modes by broadening[52], confirming that the

resulting data corresponds to thick and bulk NbS_2. Synthesis of 2H-NbS_2 was early reported to require extra sulfur ($NbS_{2.3}$) and quenching at high temperatures (~800 ^oC) [43]. 2H-NbS_2 was reported to have extra peaks at 227, 296 and 363 cm^{-1} which appear at temperatures above 900 ^oC. In combination with a decrease of the broad peak in 100-200 cm^{-1} region it suggests increase of the sulfur vacancies which was reported in literature and supports our finings that at higher temperatures stoichiometry of MeS2-x shifts towards higher x[49,53].

XPS. High resolution XPS spectra of Nb 3d and S 2p are shown in Fig. S21. XPS spectra reveals presence of both Nb and S in the obtained material. Peaks position correspond well to those for NbS_2 reported in the literature[54–56]. Peak pair at 203.2 eV and 206.0 eV are assigned to Nb $3d_{5/2}$ and Nb $3d_{3/2}$, respectively, representing Nb^{+4} from NbS_2. Peaks of sulfur at 160.4 and 161.6 eV as well as at 161.3 and 162.3 are assigned to S $2p_{3/2}$ and S $2p_{1/2}$, respectively and match sulfur in the oxidation state S^{-2}. Presence of two pairs of S 2p support mixture of 2 phases (2H and 3R), observed for both, 700 and 1000 ^oC reaction temperatures[54]. At lower sulfurization temperatures the presence of the Nb in higher oxidation state (+5) is observed with assigned peaks at 207.4 eV (Nb $3d_{5/2}$) and 210.2 eV (Nb $3d_{3/2}$)[55,56]. For material synthesized at higher temperature the peaks corresponding to the Nb_2O_5 disappear, suggesting that a more stable form of NbS_2 is formed which did not oxidize when exposed to ambient conditions as in case with many other sulfides reported in present work.

HRTEM. High resolution TEM image of NbS_2 is presented in Fig. S23. Analyzed interatomic distance was determined to be 0.30 nm.

Tantalum sulfide

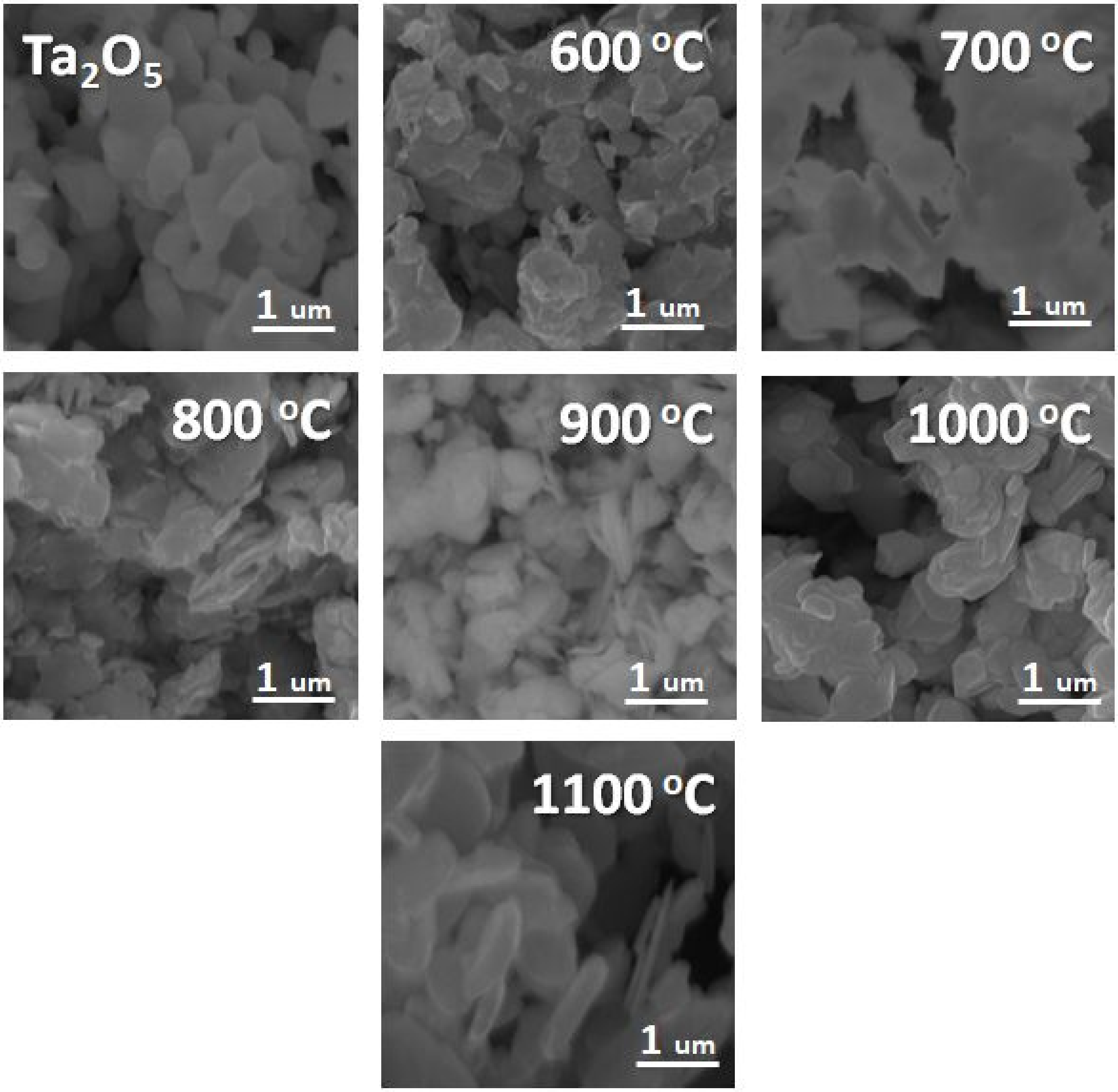

Figure S23. SEM images of Ta sulfide(s) prepared at different temperatures.

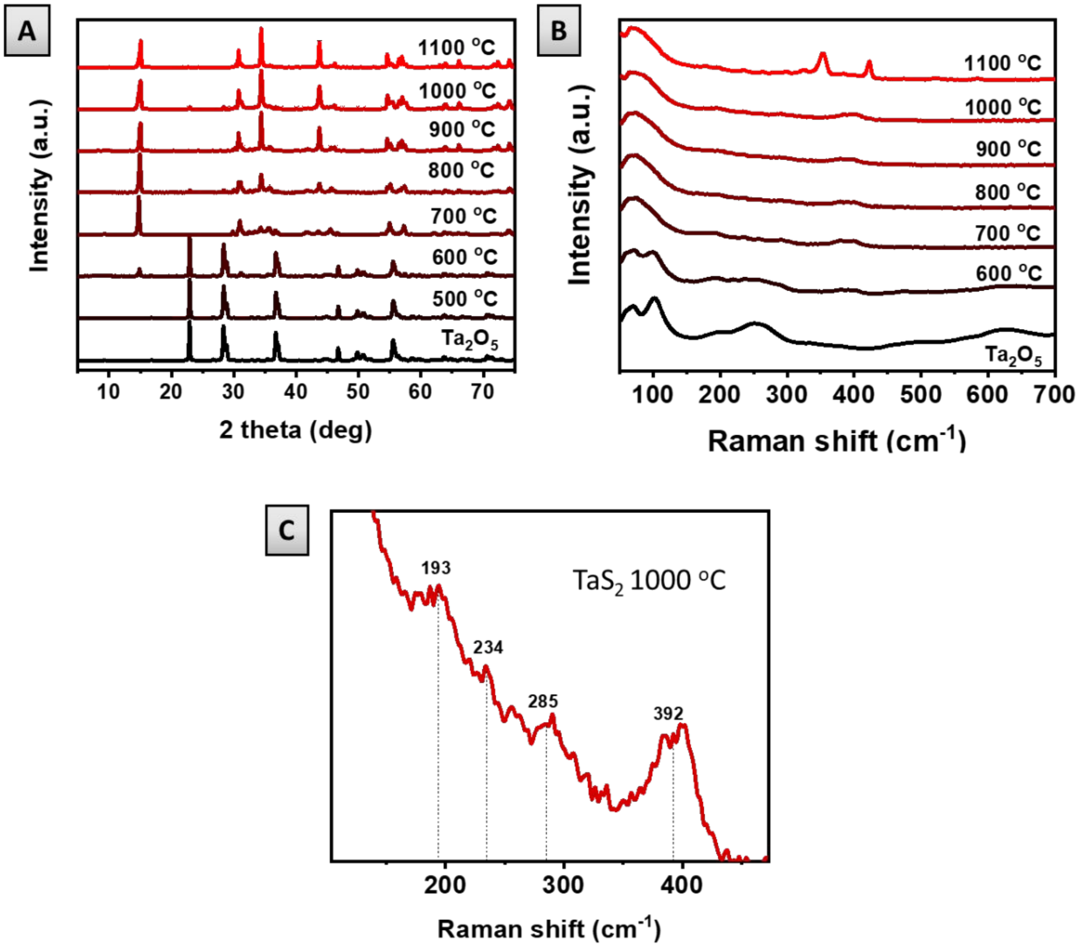

Figure S24. XRD patterns (A) and Raman spectra (B) of Ta sulfide(s) prepared at different temperatures. Low wavenumber spectrum of TaS_2 (prepared at 1000 °C) is presented separately for better visibility (C).

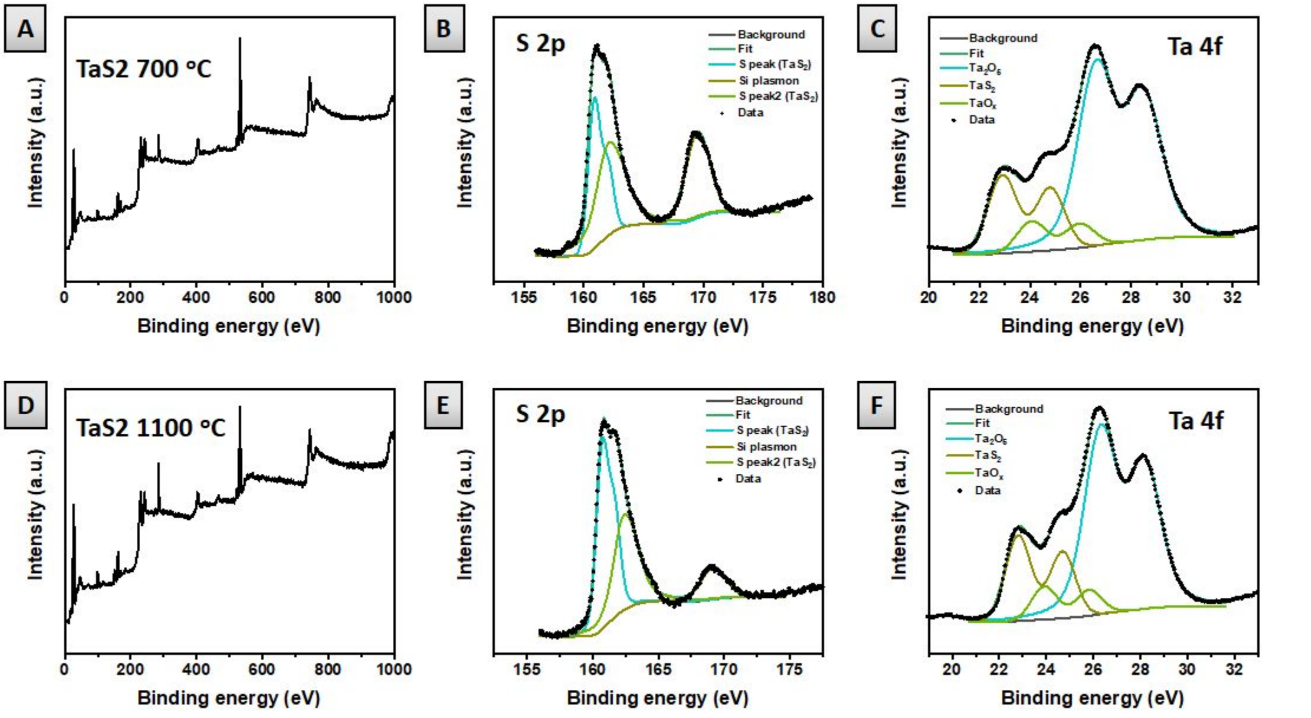

Figure S25. Survey XPS spectra of Ta sulfides (A, D) and deconvolution of characteristic S (B, E) and Ta (C, F) peaks details for materials synthesized at 700 ^{o}C (top) and 1100 ^{o}C (bottom).

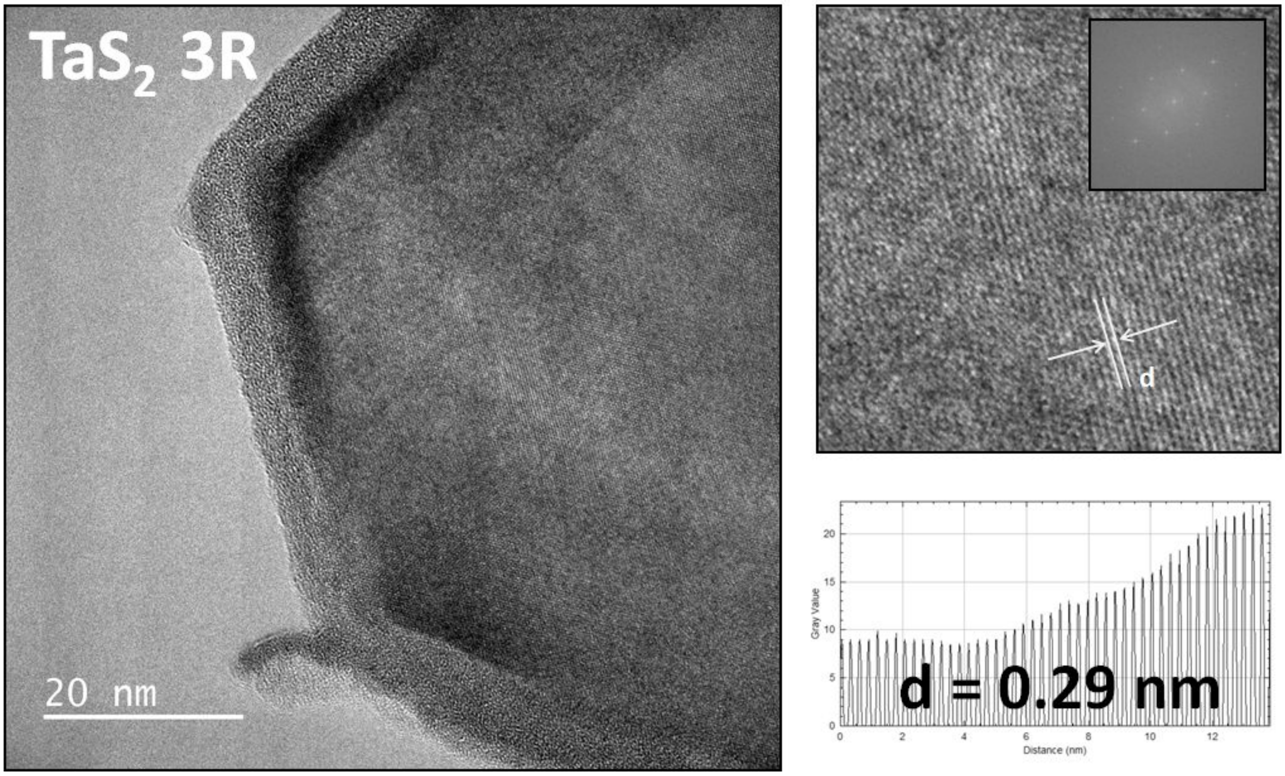

Figure S26. High resolution TEM image of TaS_2 sulfide (after exfoliation, 3R phase).

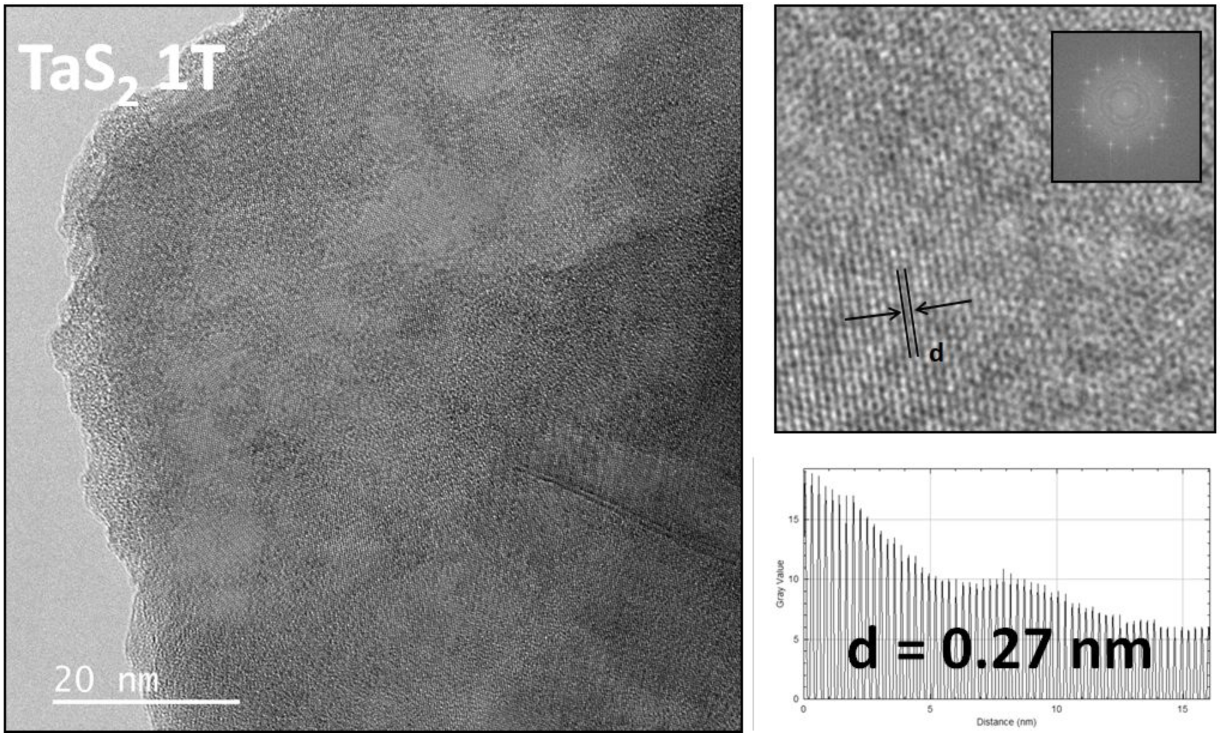

Figure S27. High resolution TEM image of TaS_2 sulfide (after exfoliation, 1T phase).

Figures S24-S27 – related discussion

XRD. Phase and quality of the as grown TaS_2 examined by the XRD analysis are shown in Fig. S24. It can be seen that at the temperature of 600 ^oC, the peaks of TaS_2 appear, but those of oxide remain still dominant. It was already shown in our previous work[1] that entire sulfurization is achieved at 650 ^oC. At 700 ^oC entire sulfurization is achieved, corresponding to 3R phase. Further temperature increase results into peaks shift toward 1T phase, this finding being in good agreement with previously reported results. Raising synthesis temperature to 1100 ^oC results in better phase transition towards 1T-TaS_2.

Raman. Raman analysis was carried out to investigate the structural properties of the obtained tantalum sulfide (Fig S24). When the synthesis temperature is increased the oxide peaks disappear. The magnified part of Raman spectra is shown separately in Fig. S24, C. Characteristic peaks were observed at 193, 234, 285 and 392 cm^{-1}, in good agreement with literature[57–59]. The relative. intensity of the peak at 285 cm^{-1} increases with increasing temperature, which can be related to an increase of the flake thickness[57]. The observation supports the general trend of flake thickening with the temperature increase as observed by SEM (Fig. S23). Origin of two additional peaks at 353 and 422 cm^{-1} observed from the material synthesized at 1100 ^oC is ambiguous and will be a subject of further investigation.

XPS. High resolution XPS spectra of Ta 4f and S 2p are shown in Fig. S25. XPS spectra reveals presence of both Ta and S in the obtained material. Peak pairs of Ta 4f at 22.8 and 24.7 eV which arose from Ta^{4+} $4f_{7/2}$ and Ta^{4+} $4f_{5/2}$ orbitals, respectively can be attributed to tantalum sulfide. Additional pair of peaks corresponding to the same orbitals but at slightly higher energies – 23.9 and 25.8 eV are also attributed to TaS_2 and are in good agreement with literature[60,61]. Another pair of peaks at 26.4 and 28.1 eV are related to Ta^{5+} $4f_{5/2}$ and Ta^{5+} $4f_{5/2}$, respectively. Observed peaks for Ta^{5+} might represent Ta_2O_5 and were normally observed for the material stored in ambient conditions[62]. The S 2p spectra shows two well resolved peaks at 160.9 and 162.3 eV, representing S $2p_{3/2}$ and S $p_{1/2}$. Increase of the temperature and phase transition does not affect the peaks position and intensity significantly, which is in good agreement with the literature[63]. Obtained XPS spectra correspond well with those of TaS_2 reported in the literature[60–63].

Chromium sulfide

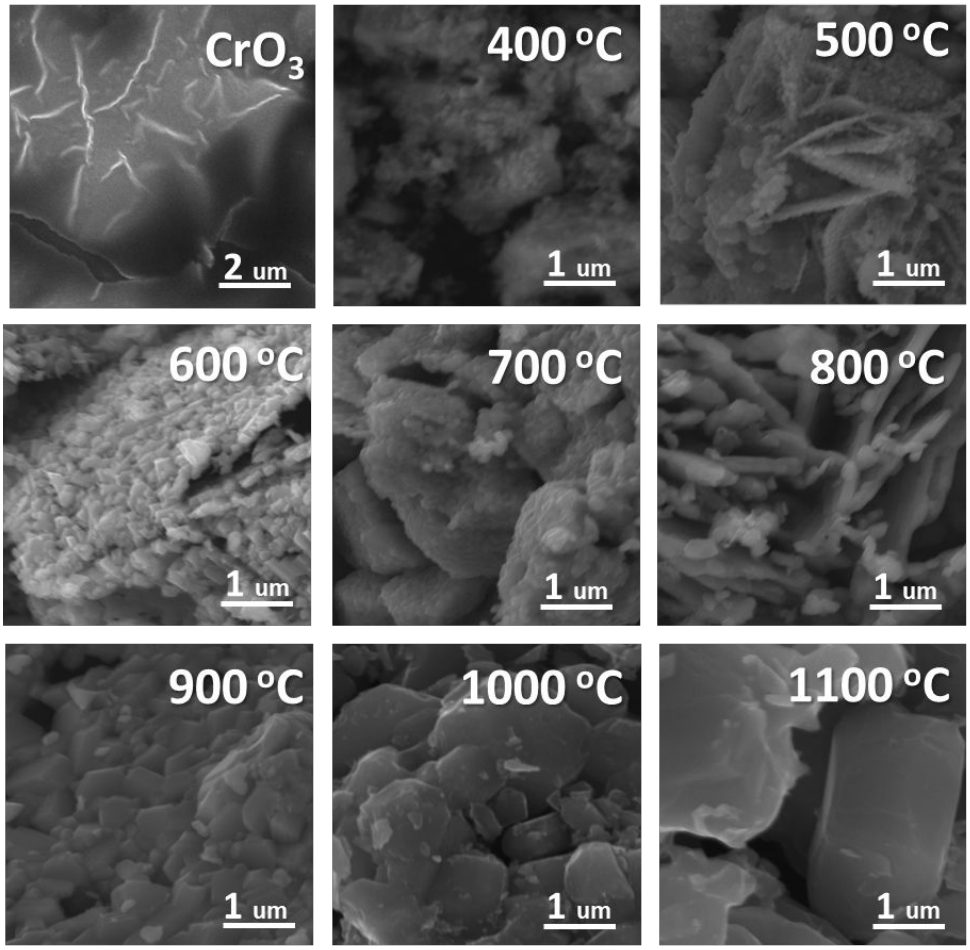

Figure S28. SEM images of Cr sulfide(s) prepared at different temperatures.

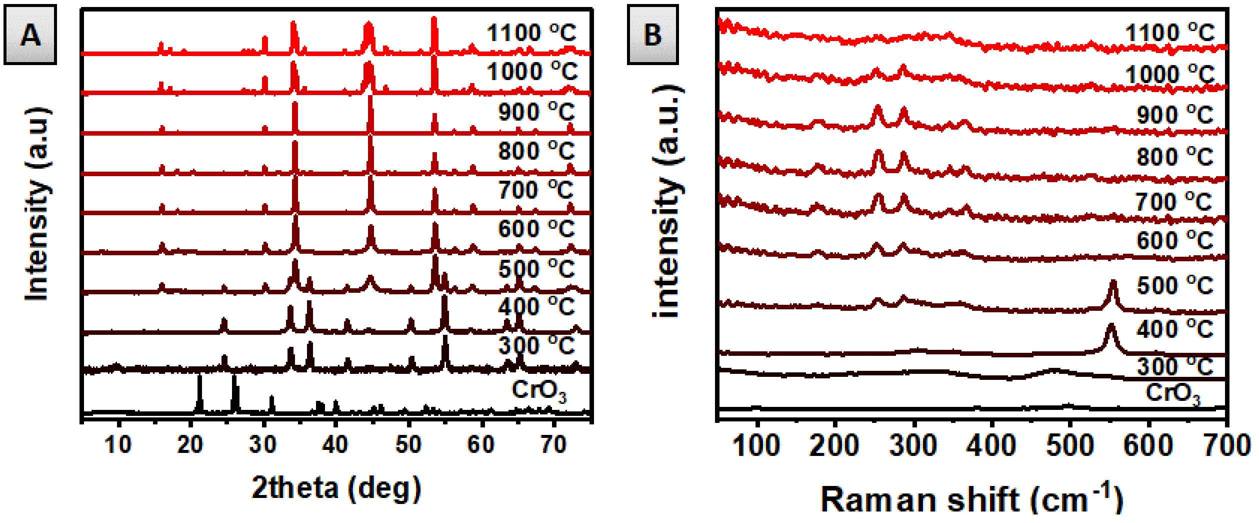

Figure S29. XRD patterns (A) and Raman spectra (B) of Cr sulfide(s) prepared at different temperatures.

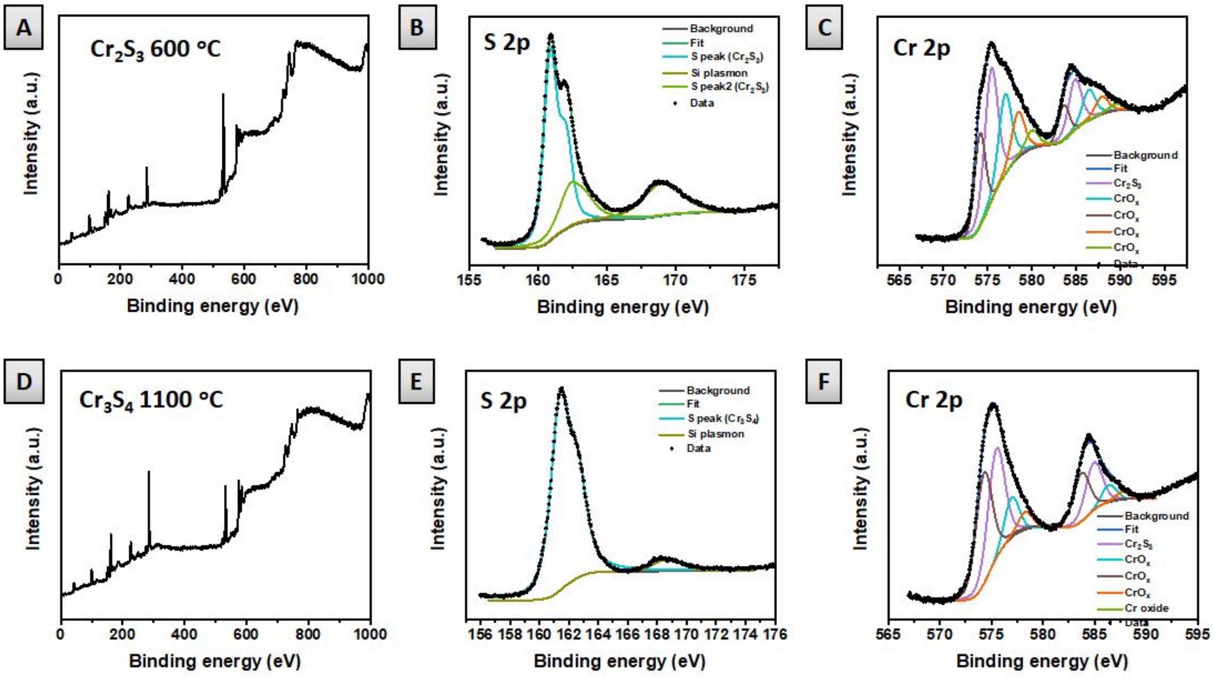

Figure S30. Survey XPS spectra of Cr sulfides (A, D) and deconvolution of characteristic S (B, E) and Cr (C, F) peaks details for materials synthesized at 600 °C (top) and 1100 °C (bottom).

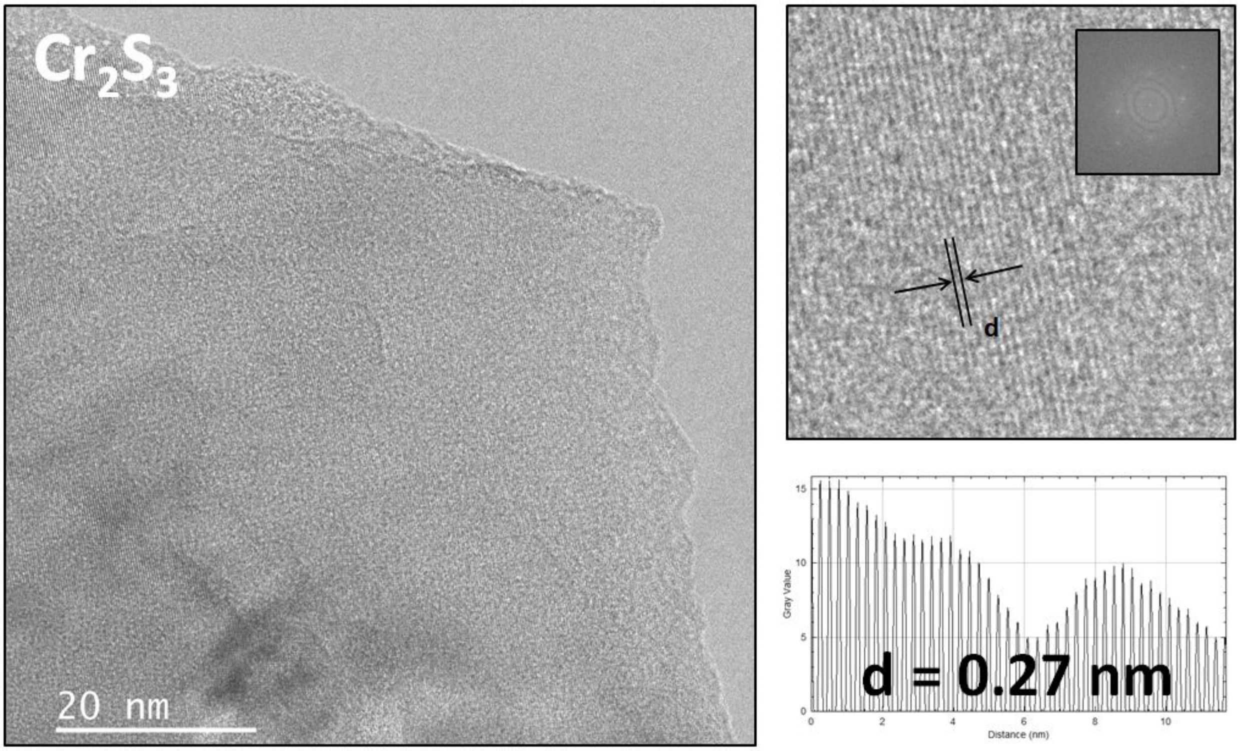

Figure S31. High resolution TEM image of Cr sulfide (after exfoliation, Cr_2S_3, sulfurization was performed at 600 ^{o}C).

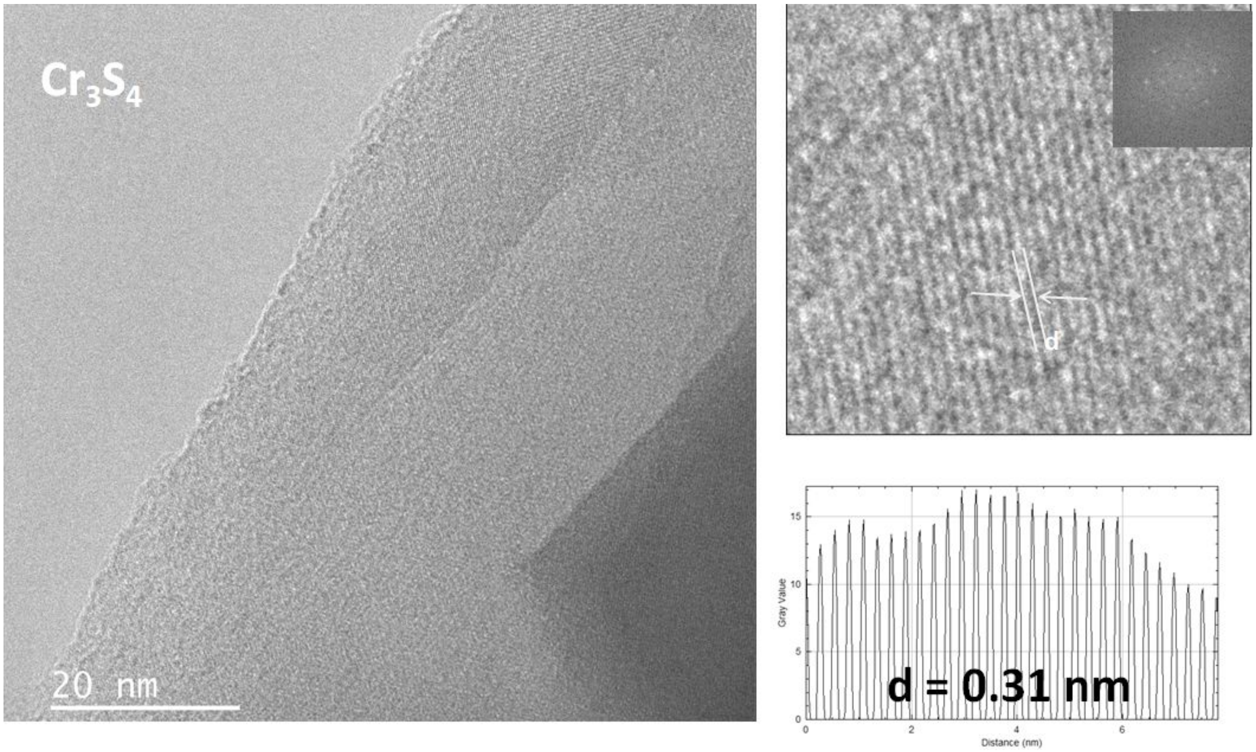

Figure S32. High resolution TEM image of Cr sulfide (after exfoliation, CrS_{2-x} was created at 1100 ^{o}C).

Figures S28-S31 – related discussion

XRD. At low temperatures (<400C) chromium is reduced from CrO_3 to Cr_2O_3 (ref. # 96-901-5001 or # 96-901-6214). Temperature increase results in the sulfurization, but 600 ^{o}C is required to obtain XRD pattern without oxide peaks indicating formation of Cr_2S_3 (ref. # 03-065-6339). Sulfurization at higher temperatures resulted in the Cr_3S_4 formation (ref. # 01-089-0404).

Raman. Obtained Raman spectra of the chromium sulfide synthesized in the range 500 – 1000°C show peaks at 178, 253, 286 and 366 cm^{-1} which correspond to the resonant frequencies observed in Cr_2S_3 as well as Cr_3S_4[6465,66]. However, these peaks are most pronounced in the range 700-900 ^{o}C, and are fading at lower and higher temperatures. The peak appearing around 555 cm^{-1} at 400 ^{o}C and further disappearing with temperature increase (1100 ^{o}C) corresponds well with the presence of chromium in lower oxidation state[67,68].

XPS. High resolution XPS spectra of Cr 2p and S 2p are shown in Fig. S30. XPS spectra reveals presence of both Cr and S in the obtained material. Cr 2p spectra is represented by two peaks at 575.2 and 584.5 eV attributed to Cr $2p_{3/2}$ and Cr $2p_{1/2}$ orbitals respectively. S $2p_{3/2}$ and S $2p_{1/2}$ corresponding peaks are positioned at 160.9 and 162.0, respectively, which is in good agreement with reported values for chromium sulfide[65,66,69,70]. Presence of oxide-related peaks can be attributed to a post-preparative oxidation, during the samples contact with air.

HRTEM. High resolution TEM image of Cr_2S_3 is presented in Fig. S31. Analysis of the HRTEM images of obtained chromium sulfide shows the interatomic distance of 0.27 nm, which is in agreement with literature[71]. Sulfurization at higher temperature and “loss” of sulfur atom results in interatomic distance increase up to 0.31 nm.

Molybdenum sulfide

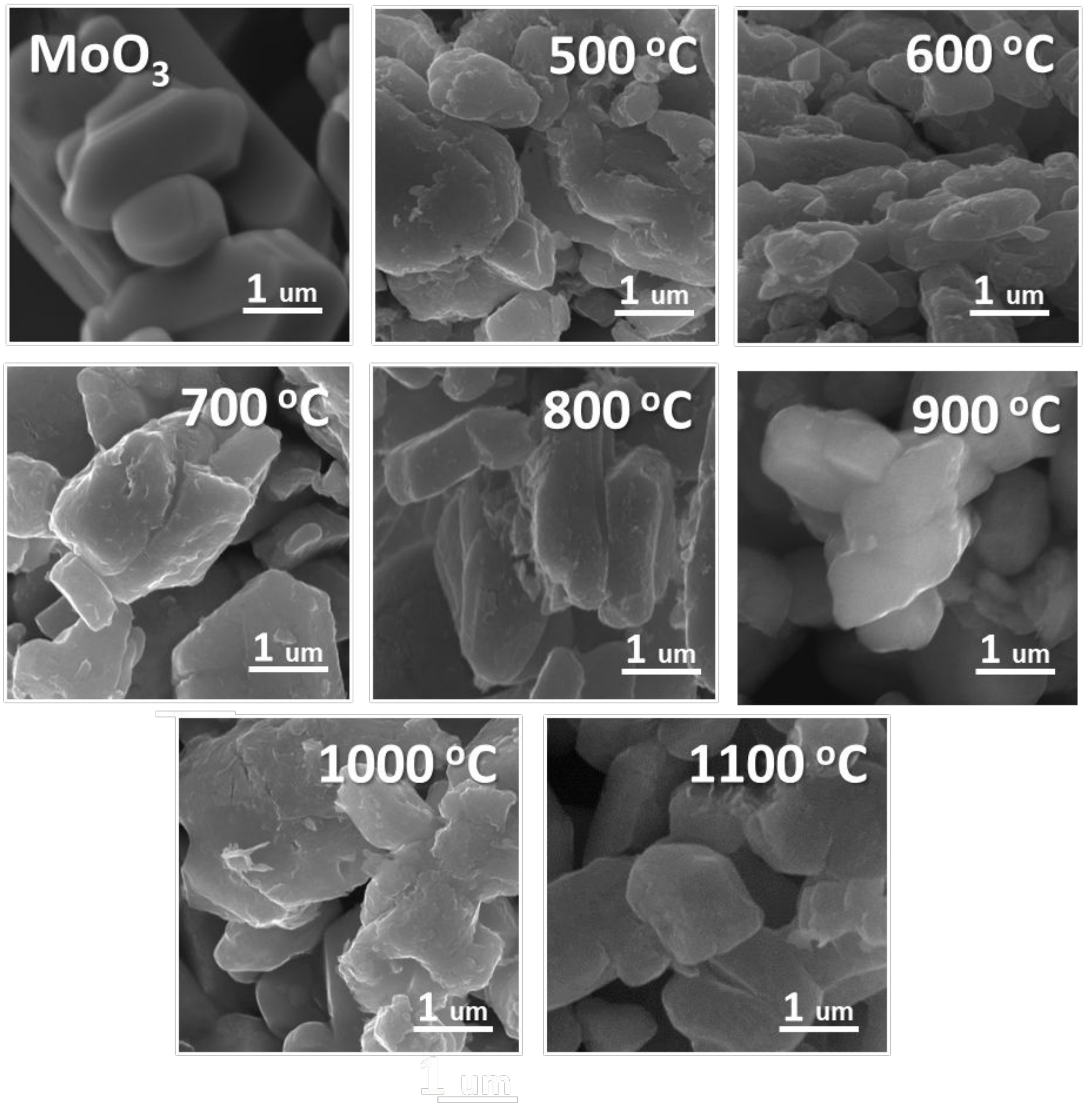

Figure S33. SEM images of Mo sulfide(s) prepared at different temperatures.

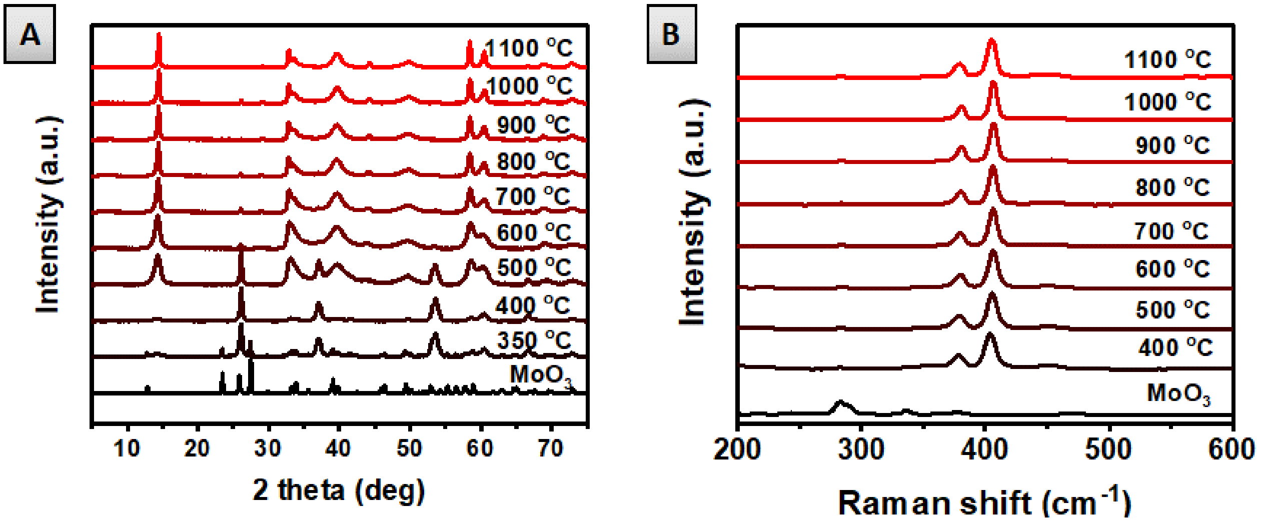

Figure S34. XRD patterns (A) and Raman spectra (B) of Mo sulfide(s) prepared at different temperatures.

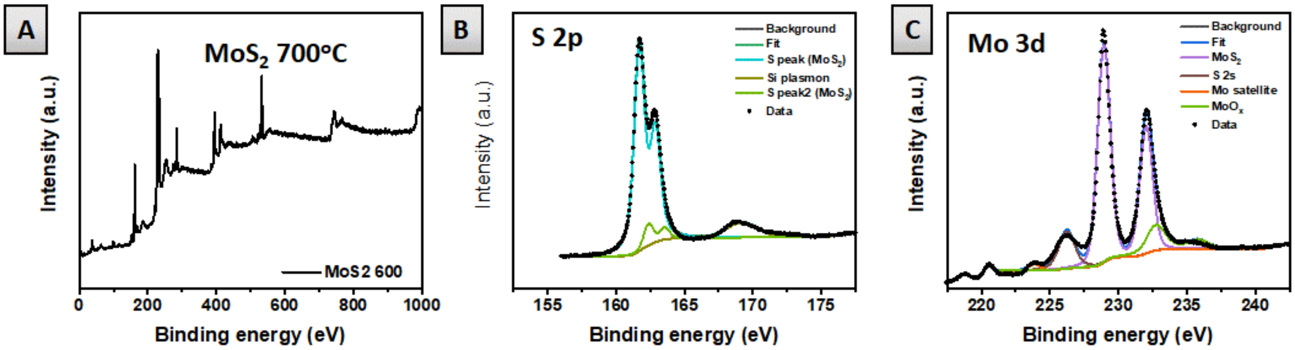

Figure S35. Survey XPS spectra of Mo sulfide (A) and deconvolution of characteristic S (B) and Mo (C) peaks details.

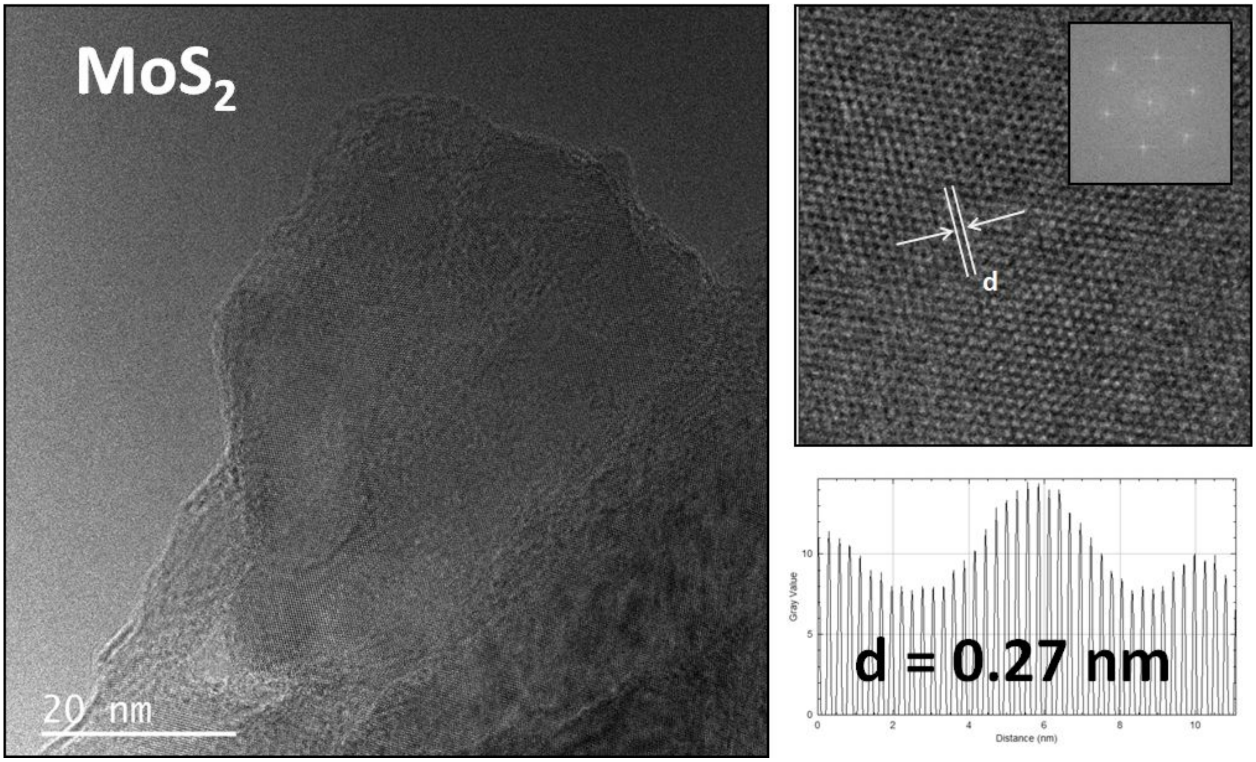

Figure S36. High resolution TEM image of Mo sulfide (after exfoliation).

Figures S33-S36 – related discussion

XRD. Reduction of MoO_3 starts around 350 ^oC, leading to MoO_2 at 400C (ref # 96-900-9091) when sulfide peaks appear too. At 700 ^oC entire sulfurization to MoS_2 takes place (ref. # 01-073-1508), and the oxide peaks disappear. Further increase of the temperature to 1100 ^oC does not cause phase changes, but peak(s) sharpness is increased, which effect can be explained by higher crystallinity caused by the increase of the crystal size.

Raman. Characteristic peaks for MoS_2 were observed at 379 and 406 cm^{-1}, attributed to E^1_{2g} and A_{1g} which is in good agreement with literature[72].

XPS. High resolution XPS spectra of Mo 3d and S 2p are shown in Fig. S35 XPS spectra confirm presence of both Mo and S in the obtained material. Mo 3d spectra is represented by two peaks at 228.9 and 230.1 eV attributed to Mo $3d_{5/2}$ and Mo $3d_{3/2}$ orbitals respectively. S 2s peak can be observed at 226.2 eV. The results are in good agreement with those observed in literature[73].

HRTEM. High resolution TEM image of MoS_2 is presented in Fig.S36. Analyzed interatomic distance was determined to be 0.27 nm and being in well agreement with literature[74,75].

Tungsten sulfide

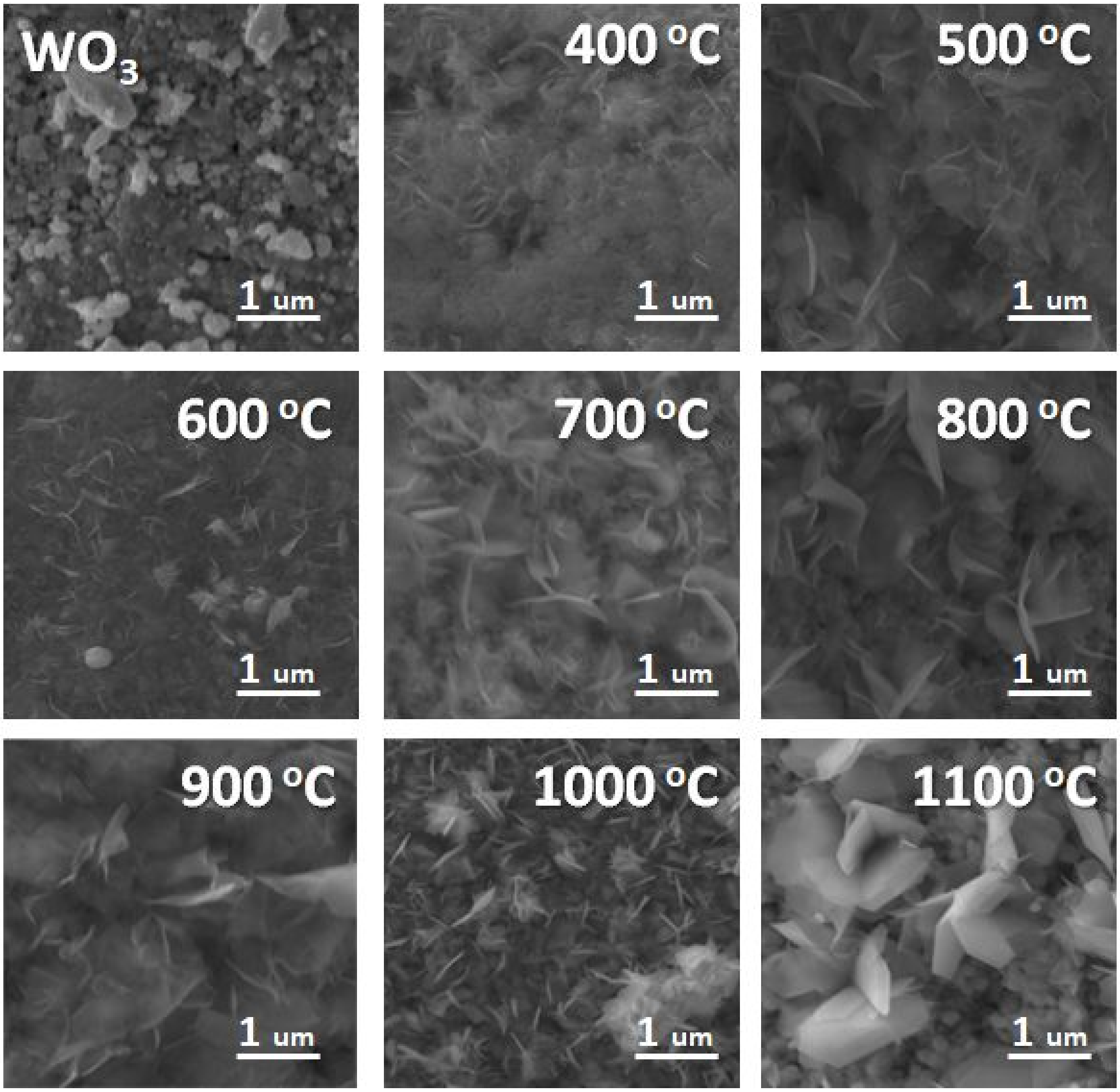

Figure S37. SEM images of W sulfide(s) prepared at different temperatures.

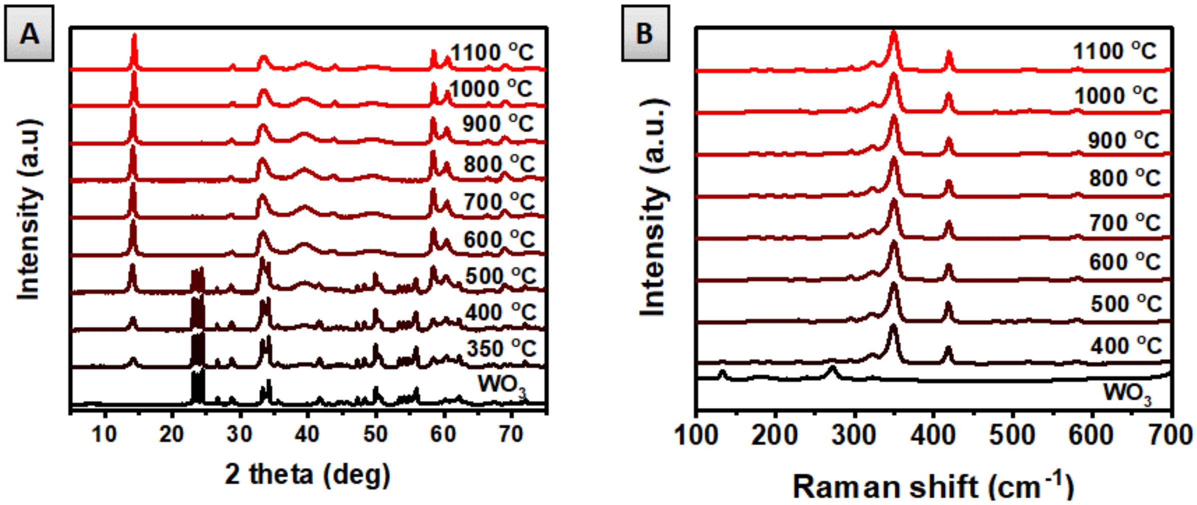

Figure S38. XRD patterns (A) and Raman spectra (B) of W sulfide(s) prepared at different temperatures.

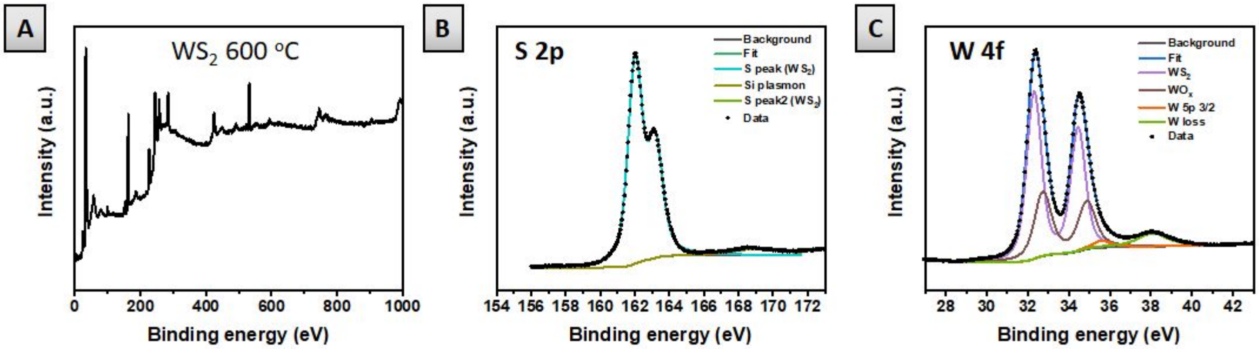

Figure S39. Survey XPS spectra of W sulfide (A) and deconvolution of characteristic S (B) and W (C) peaks details.

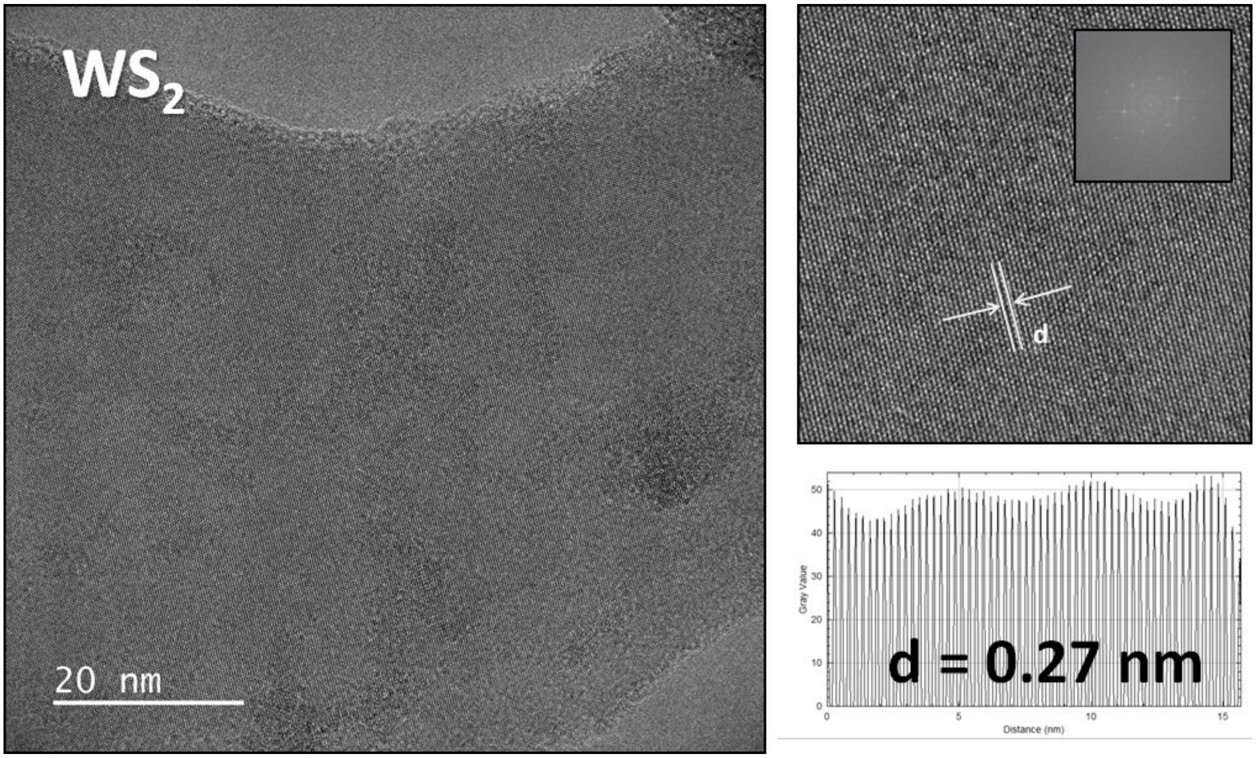

Figure S40. High resolution TEM image of W sulfide (after exfoliation)

Figures S37-40 – related discussion

XRD. Sulfide peaks in the XRD spectra assigned to WS_2 appear already at 350 ^oC, similarly to molybdenum sulfurization. However, the oxide peaks disappear completely only above 600 ^oC. As the temperature is increased further, the peak at ~14.2 degrees shifts towards 14.4, with the most significant shift occurring for 1000 and 1100 ^oC. The spectra taken at higher temperatures show better WS_2 crystallinity in comparison to that obtained at temperatures about 600 ^oC (ref. # 00-008-0237). All spectra from materials synthesized at temperatures from 600-1100 ^oC correspond to WS_2 crystal structure reported in the literature[76].

Raman. Characteristic peaks for WS_2 observed at 349 and 416 cm^{-1} attributed to E_{2g} and A_{1g} respectively are in well agreement with literature[77–79]. Stronger intensity of E_{2g} peak than of A_{1g} suggests that flakes are formed and WS_2 is not embedded in the massive bulk structure[77,80]. Additionally, decrease of E_{2g}/A_{1g} intensity ratio with increasing synthesis temperature suggests that larger thickness of the flakes is obtained at higher temperature, which is also supported by SEM images.

XPS. High resolution XPS spectra of W 4f and S 2p are shown in Fig. S39. XPS spectra reveal presence of both W and S in the obtained material. Peak pairs of W 4f at 32.4 and 34.6 eV are attributed to W $4f_{7/2}$ and W $4f_{5/2}$ orbitals, respectively, and can be attributed to tungsten sulfide. S 2s peak pair can

be observed at 162.0 and 163.1 eV. Obtained results are in good agreement with literature[81,82]. Presence of oxide-related peaks can be attributed to a post-preparative oxidation, during the samples contact with air.

HRTEM. High resolution TEM image of WS_2 is presented in Fig.S40. Interatomic distance was determined to be 0.27 nm, in well agreement with literature[77].

BET and SEM results – general description

To analyze additionally the quality of the synthesized materials we have estimated the surface area of the as synthesized bulk TMDCs powders by the Brunauer–Emmett–Teller (BET) method, (see Table S1). Adsorption and desorption nitrogen isotherms were recorded on the Nova3200 instrument (Quantachrome Instruments, USA) with the nitrogen (Linde, 99,999 % purity) at liquid nitrogen temperature and evaluated by using NovaWin software. Total specific surface area was calculated using a 5-point Brunauer-Emmet-Teller (BET) analysis and the Micropore BET Asistant. It can be seen, that as synthesized sulfides possessed low surface area which further decreases with increasing synthesis temperature as confirmed by SEM and Raman analyses, as well. Determined surface area correlates well with the results reported in the literature for bulk TiS_2 [83], MoS2 [84], WS_2 [85] and TaS2 [60], which were used for the further processing to obtain 2D materials.

Table. S1 Estimated by BET surface area of created sulfides.

MSx	BET study	
	m^2/g	(+/-)
**1T-TiS_2 (600 ^oC)**	13	0.7
**1T-TiS_2 (1000 ^oC)**	3.4	0.4
**1T-ZrS_2 (1000 ^oC)**	5.4	0.4
**1T-HfS_2 (1100 ^oC)**	6.5	0.3
**2H-V_3S_5 (500 ^oC)**	3.5	0.4
**2H-V_3S_4 (1000 ^oC)**	2.5	0.8
**3R-NbS_2 (700 ^oC)**	8.2	1.9
**3R-TaS_2 (700 ^oC)**	5.7	0.6
**1T-TaS_2 (1000 ^oC)**	11.1	1.2
**2H-Cr_2S_3 (800 ^oC)**	3.5	0.6
**2H-MoS_2 (600 ^oC)**	5.5	0.3
**2H-WS_2 (600 ^oC)**	2.7	0.3
**WS_2 (1000 ^oC)**	2.8	0.5

SEM. In general, we have observed flake thickening and lateral size growth with increase of the temperature for all the materials, which is in good agreement with literature[66].

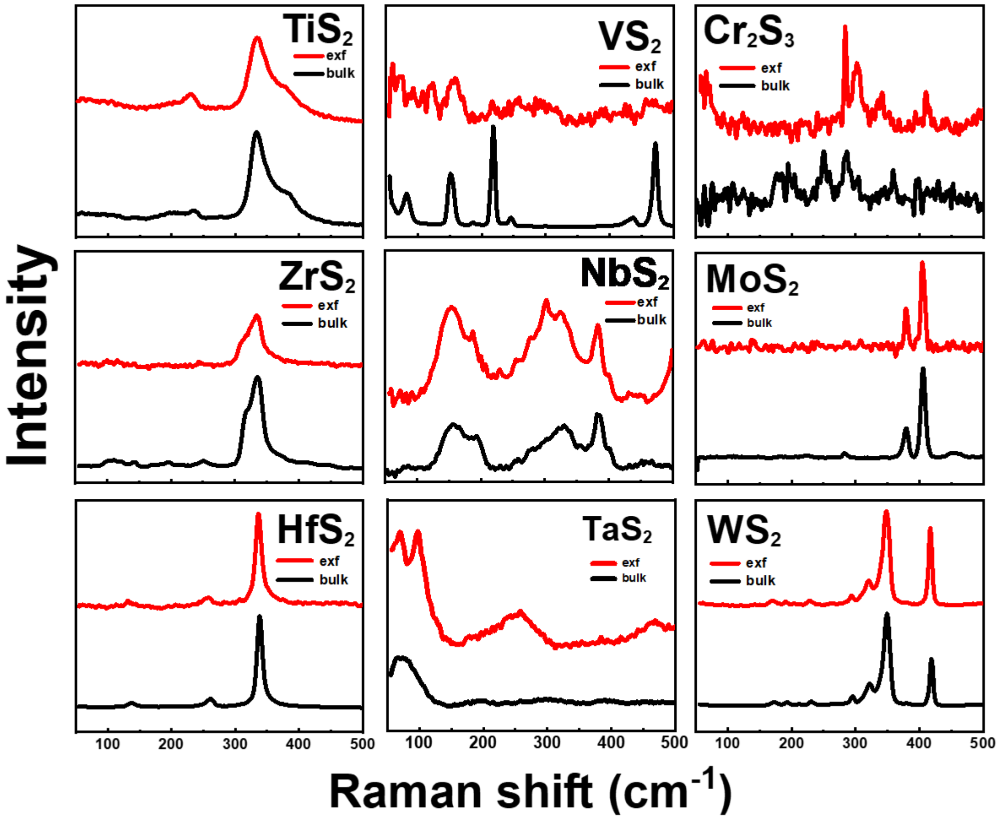

Figure S41 Raman spectra of created TMDC measure before and after exfoliation

Raman spectroscopy of bulk and exfoliated materials.

Thickness of some of the sulfides can be probed by the Raman spectroscopy. Thus, we probed the materials after the exfoliation by Raman spectroscopy and compare it with the bulk, Figure S41. In general, position of the peaks A_{1g} and E_{2g} is estimated to be shifted, nevertheless, the most significant shift is observed within the range 1-5 monolayers. Red shift from 235 to 231 was observed for TiS_2 after the exfoliation, along with the intensity decrease of the shoulder peak at 377 cm^{-1} which is consistent with the observed thickening of TiS_2 flakes[86]. For hafnium and zirconium sulfides broadening and small red shift of the A_{1g} peak (~330 cm^{-1}) after the exfoliation can be observed, which is consistent with the literature[87]. For exfoliated vanadium sulfide peak intensity is changing in comparison to bulk, with the highest intensty of the peak at 160 cm^{-1} which is consistent with the literature[88]. For the NbS2 small red shift can be observed for the A_1 peak (at ~ 390 cm^{-1}), while more significant shift can be observed for E_2 peak (at ~325 cm^{-1}), which is in good agreement with literature[89]. Interpretation of changes in spectra of TaS_2 is rather complicated due to low intensity of the signal. Chromium sulfide peaks at 180 and 360 cm^{-1} are known to vanish with the thickness decrease[90] which is consistent with obtained results. Similar

results were observed for MoS2 with the red shift of the A_{1g} peak and relative increase of E_{2g} peak was observed after the exfoliation which is in agreement with literature[91]. Fow WS_2 an increase of the peak intensity of and E_{2g} relative to A_{1g} was observed with exfoliation, which is estimated according to the literature [92].

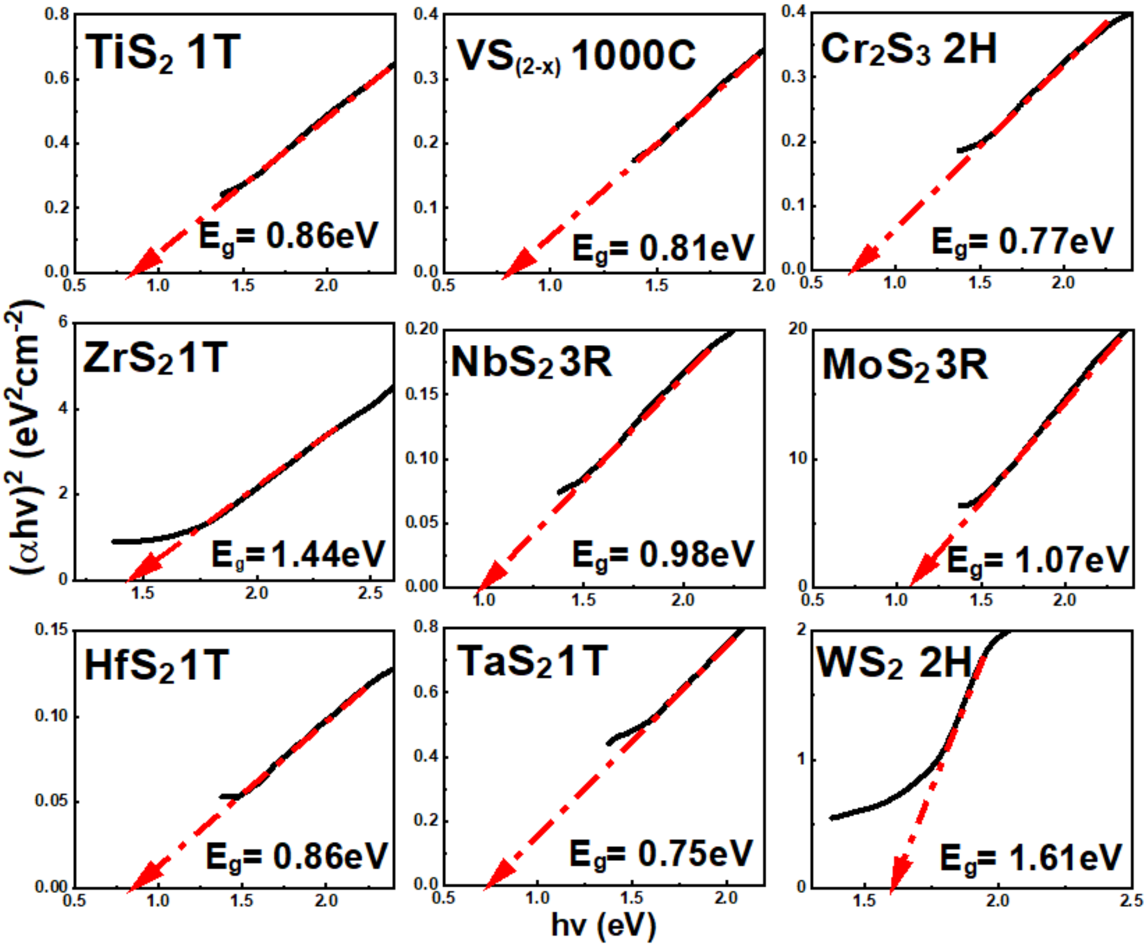

Fig. S42 Estimation of materials band gap (using Tauc plot)

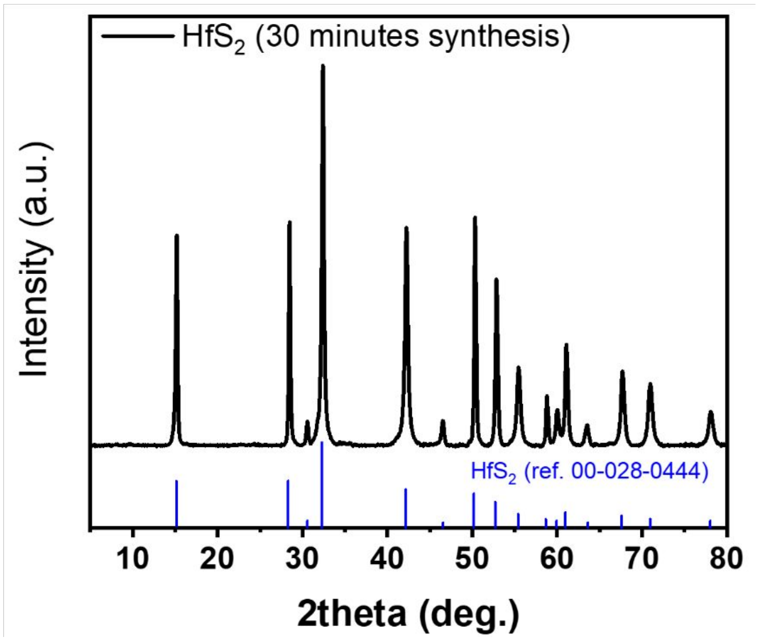

Figure S43. XRD of HfS_2 synthesized at 1100 ^{o}C for only 30 (load 100 mg).

References

(1) Buravets, V.; Hosek, F.; Lapcak, L.; Miliutina, E.; Sajdl, P.; Elashnikov, R.; Švorčík, V.; Lyutakov, O. Beyond the Platinum Era–Scalable Preparation and Electrochemical Activation of TaS_2 Flakes. *ACS Appl. Mater. Interfaces* **2023**, *15* (4), 5679–5686. https://doi.org/10.1021/acsami.2c20261.

(2) Wang, H.; Lv, W.; Shi, J.; Wang, H.; Wang, D.; Jin, L.; Chao, J.; van Aken, P. A.; Chen, R.; Huang, W. Efficient Liquid Nitrogen Exfoliation of MoS_2 Ultrathin Nanosheets in the Pure 2H Phase. *ACS Sustain. Chem. Eng.* **2020**, *8* (1), 84–90. https://doi.org/10.1021/acssuschemeng.9b04057.

(3) Wang, Y.; Liu, Y.; Zhang, J.; Wu, J.; Xu, H.; Wen, X.; Zhang, X.; Tiwary, C. S.; Yang, W.; Vajtai, R.; Zhang, Y.; Chopra, N.; Odeh, I. N.; Wu, Y.; Ajayan, P. M. Cryo-Mediated Exfoliation and Fracturing of Layered Materials into 2D Quantum Dots. *Sci. Adv.* **2017**, *3* (12), e1701500. https://doi.org/10.1126/sciadv.1701500.

(4) Zhang, J.; Wang, Y.; Cui, J.; Wu, J.; Li, Y.; Zhu, T.; Kang, H.; Yang, J.; Sun, J.; Qin, Y.; Zhang, Y.; Ajayan, P. M.; Wu, Y. Water-Soluble Defect-Rich MoS_2 Ultrathin Nanosheets for Enhanced Hydrogen Evolution. *J. Phys. Chem. Lett.* **2019**, *10* (12), 3282–3289. https://doi.org/10.1021/acs.jpclett.9b01121.

(5) Ohta, M.; Satoh, S.; Kuzuya, T.; Hirai, S.; Kunii, M.; Yamamoto, A. Thermoelectric Properties of $Ti_{1+x}S_2$ Prepared by CS2 Sulfurization. *Acta Mater.* **2012**, *60* (20), 7232–7240. https://doi.org/10.1016/j.actamat.2012.09.035.

(6) Ahmadi, E.; Yashima, Y.; Suzuki, R. O.; Rezan, S. A. Formation of Titanium Sulfide from Titanium Oxycarbonitride by CS_2 Gas. *Metall Mater. Trans. B* **2018**, *49* (4), 1808–1821. https://doi.org/10.1007/s11663-018-1278-8.

(7) Sherrell, P. C.; Sharda, K.; Grotta, C.; Ranalli, J.; Sokolikova, M. S.; Pesci, F. M.; Palczynski, P.; Bemmer, V. L.; Mattevi, C. Thickness-Dependent Characterization of Chemically Exfoliated TiS_2 Nanosheets. *ACS Omega* **2018**, *3* (8), 8655–8662. https://doi.org/10.1021/acsomega.8b00766.

(8) Chen, X.; Song, M.; Zhang, L.; Zhang, R.; Zhang, L.; Tong, W.; Han, Y.; Gao, X.; Xiong, Y.; Xu, H.; Cao, L. Defect-Dependent Surface Phase Transformation on 1*T*-TiS_2 Assisted by Water. *J. Phys. Chem. C* **2023**, *127* (7), 3462–3469. https://doi.org/10.1021/acs.jpcc.2c08140.

(9) Dużyńska, A.; Judek, J.; Wilczyński, K.; Zberecki, K.; Łapińska, A.; Wróblewska, A.; Zdrojek, M. Temperature-Induced Phonon Behavior in Titanium Disulfide (TiS_2) Nanosheets. *J. Raman Spectrosc.* **2019**, *50* (8), 1114–1119. https://doi.org/10.1002/jrs.5637.

(10) Liu, Y.; Liang, C.; Wu, J.; Sharifi, T.; Xu, H.; Nakanishi, Y.; Yang, Y.; Woellne, C. F.; Aliyan, A.; Martí, A. A.; Xie, B.; Vajtai, R.; Yang, W.; Ajayan, P. M. Atomic Layered Titanium Sulfide Quantum Dots as Electrocatalysts for Enhanced Hydrogen Evolution Reaction. *Adv. Mater. Interfaces* **2018**, *5* (1), 1700895. https://doi.org/10.1002/admi.201700895.

(11) Sun, K.; Zhang, Q.; Bock, D. C.; Tong, X.; Su, D.; Marschilok, A. C.; Takeuchi, K. J.; Takeuchi, E. S.; Gan, H. Interaction of TiS_2 and Sulfur in Li-S Battery System. *J. Electrochem. Soc.* **2017**, *164* (6), A1291–A1297. https://doi.org/10.1149/2.1631706jes.

(12) Martinez, H.; Auriel, C.; Gonbeau, D.; Loudet, M.; Pfister-Guillouzo, G. Studies of 1T TiS2 by STM, AFM and XPS: The Mechanism of Hydrolysis in Air. *Appl. Surf. Sci.* **1996**, *93* (3), 231–235. https://doi.org/10.1016/0169-4332(95)00339-8.

(13) Gao, Z.; Ji, Q.; Shen, P.-C.; Han, Y.; Leong, W. S.; Mao, N.; Zhou, L.; Su, C.; Niu, J.; Ji, X.; Goulamaly, M. M.; Muller, D. A.; Li, Y.; Kong, J. In Situ-Generated Volatile Precursor for CVD Growth of a Semimetallic 2D Dichalcogenide. *ACS Appl. Mater. Interfaces* **2018**, *10* (40), 34401–34408. https://doi.org/10.1021/acsami.8b13428.

(14) Toh, R. J.; Sofer, Z.; Pumera, M. Catalytic Properties of Group 4 Transition Metal Dichalcogenides (MX_2 ; M = Ti, Zr, Hf; X = S, Se, Te). *J. Mater. Chem. A* **2016**, *4* (47), 18322–18334. https://doi.org/10.1039/C6TA08089H.

(15) Huckaba, A. J.; Gharibzadeh, S.; Ralaiarisoa, M.; Roldán-Carmona, C.; Mohammadian, N.; Grancini, G.; Lee, Y.; Amsalem, P.; Plichta, E. J.; Koch, N.; Moshaii, A.; Nazeeruddin, M. K. Low-Cost TiS2 as Hole-Transport Material for Perovskite Solar Cells. *Small Methods* **2017**, *1* (10), 1700250. https://doi.org/10.1002/smtd.201700250.

(16) Luo, Q.; Tian, R.; Wu, A.; Dong, X.; Jin, X.; Zhou, S.; Huang, H. In-Built Durable Li–S Counterparts from Li–TiS_2 Batteries. *Mater. Today Energy* **2020**, *17*, 100439. https://doi.org/10.1016/j.mtener.2020.100439.

(17) Dang, V. Q.; Al-Ali, K. The Synthesis and Investigation of the Reversible Conversion of Layered ZrS $_2$ and ZrS_3. *New J. Chem.* **2020**, *44* (18), 7583–7590. https://doi.org/10.1039/C9NJ05631A.

(18) Mañas-Valero, S.; García-López, V.; Cantarero, A.; Galbiati, M. Raman Spectra of ZrS_2 and $ZrSe_2$ from Bulk to Atomically Thin Layers. *Appl. Sci.* **2016**, *6* (9), 264. https://doi.org/10.3390/app6090264.

(19) Oliver, S. M.; Fox, J. J.; Hashemi, A.; Singh, A.; Cavalero, R. L.; Yee, S.; Snyder, D. W.; Jaramillo, R.; Komsa, H.-P.; Vora, P. M. Phonons and Excitons in $ZrSe_2$ –ZrS_2 Alloys. *J. Mater. Chem. C* **2020**, *8* (17), 5732–5743. https://doi.org/10.1039/D0TC00731E.

(20) Martino, E.; Santos-Cottin, D.; Le Mardelé, F.; Semeniuk, K.; Pizzochero, M.; Čerņevičs, K.; Baptiste, B.; Delbes, L.; Klotz, S.; Capitani, F.; Berger, H.; Yazyev, O. V.; Akrap, A. Structural Phase Transition and Bandgap Control through Mechanical Deformation in Layered Semiconductors 1T–ZrX_2 (X = S, Se). *ACS Mater. Lett.* **2020**, *2* (9), 1115–1120. https://doi.org/10.1021/acsmaterialslett.0c00252.

(21) Wang, X.; Huang, L.; Jiang, X.-W.; Li, Y.; Wei, Z.; Li, J. Large Scale ZrS_2 Atomically Thin Layers. *J. Mater. Chem. C* **2016**, *4* (15), 3143–3148. https://doi.org/10.1039/C6TC00254D.

(22) Mattinen, M.; Popov, G.; Vehkamäki, M.; King, P. J.; Mizohata, K.; Jalkanen, P.; Räisänen, J.; Leskelä, M.; Ritala, M. Atomic Layer Deposition of Emerging 2D Semiconductors, HfS_2 and ZrS_2, for Optoelectronics. *Chem. Mater.* **2019**, *31* (15), 5713–5724. https://doi.org/10.1021/acs.chemmater.9b01688.

(23) Shimazu, Y.; Fujisawa, Y.; Arai, K.; Iwabuchi, T.; Suzuki, K. Synthesis and Characterization of Zirconium Disulfide Single Crystals and Thin-Film Transistors Based on Multilayer Zirconium Disulfide Flakes. *ChemNanoMat* **2018**, *4* (10), 1078–1082. https://doi.org/10.1002/cnma.201800304.

(24) Jin, Y.; Sun, J.; Zhang, L.; Yang, J.; Wu, Y.; You, B.; Liu, X.; Leng, K.; Liu, S. Controllable Oxidation of ZrS_2 to Prepare High-κ, Single-Crystal m-ZrO_2 for 2D Electronics. *Adv. Mater.* **2023**, *35* (18), 2212079. https://doi.org/10.1002/adma.202212079.

(25) Hamada, M.; Matsuura, K.; Sakamoto, T.; Muneta, I.; Hoshii, T.; Kakushima, K.; Tsutsui, K.; Wakabayashi, H. High Hall-Effect Mobility of Large-Area Atomic-Layered Polycrystalline ZrS_2 Film Using UHV RF Magnetron Sputtering and Sulfurization. *IEEE J. Electron Devices Soc.* **2019**, *7*, 1258–1263. https://doi.org/10.1109/JEDS.2019.2943609.
(26) Cao, Y.; Wähler, T.; Park, H.; Will, J.; Prihoda, A.; Moses Badlyan, N.; Fromm, L.; Yokosawa, T.; Wang, B.; Guldi, D. M.; Görling, A.; Maultzsch, J.; Unruh, T.; Spiecker, E.; Halik, M.; Libuda, J.; Bachmann, J. Area-Selective Growth of HfS_2 Thin Films via Atomic Layer Deposition at Low Temperature. *Adv. Mater. Interfaces* **2020**, *7* (23), 2001493. https://doi.org/10.1002/admi.202001493.
(27) Hong, M.; Dai, L.; Hu, H.; Zhang, X.; Li, C.; He, Y. High-Pressure Structural Phase Transitions and Metallization in Layered HfS_2 under Different Hydrostatic Environments up to 42.1 GPa. *J. Mater. Chem. C* **2022**, *10* (29), 10541–10550. https://doi.org/10.1039/D2TC01669A.
(28) Antoniazzi, I.; Zawadzka, N.; Grzeszczyk, M.; Woźniak, T.; Ibáñez, J.; Muhammad, Z.; Zhao, W.; Molas, M. R.; Babiński, A. The Effect of Temperature and Excitation Energy on Raman Scattering in Bulk. *J. Phys.: Condens. Matter* **2023**, *35* (30), 305401. https://doi.org/10.1088/1361-648X/acce18.
(29) Mirabelli, G.; McGeough, C.; Schmidt, M.; McCarthy, E. K.; Monaghan, S.; Povey, I. M.; McCarthy, M.; Gity, F.; Nagle, R.; Hughes, G.; Cafolla, A.; Hurley, P. K.; Duffy, R. Air Sensitivity of MoS_2, $MoSe_2$, $MoTe_2$, HfS_2, and $HfSe_2$. *J. Appl. Phys.* **2016**, *120* (12), 125102. https://doi.org/10.1063/1.4963290.
(30) Wang, D.; Meng, J.; Zhang, X.; Guo, G.; Yin, Z.; Liu, H.; Cheng, L.; Gao, M.; You, J.; Wang, R. Selective Direct Growth of Atomic Layered HfS_2 on Hexagonal Boron Nitride for High Performance Photodetectors. *Chem. Mater.* **2018**, *30* (11), 3819–3826. https://doi.org/10.1021/acs.chemmater.8b01091.
(31) Kawada, I.; Nakano-Onoda, M.; Ishii, M.; Saeki, M.; Nakahira, M. Crystal Structures of V_3S_4 and V_5S_8. *J. Solid State Chem.* **1975**, *15* (3), 246–252. https://doi.org/10.1016/0022-4596(75)90209-1.
(32) Ji, Q.; Li, C.; Wang, J.; Niu, J.; Gong, Y.; Zhang, Z.; Fang, Q.; Zhang, Y.; Shi, J.; Liao, L.; Wu, X.; Gu, L.; Liu, Z.; Zhang, Y. Metallic Vanadium Disulfide Nanosheets as a Platform Material for Multifunctional Electrode Applications. *Nano Lett.* **2017**, *17* (8), 4908–4916. https://doi.org/10.1021/acs.nanolett.7b01914.
(33) Yuan, J.; Wu, J.; Hardy, W. J.; Loya, P.; Lou, M.; Yang, Y.; Najmaei, S.; Jiang, M.; Qin, F.; Keyshar, K.; Ji, H.; Gao, W.; Bao, J.; Kono, J.; Natelson, D.; Ajayan, P. M.; Lou, J. Facile Synthesis of Single Crystal Vanadium Disulfide Nanosheets by Chemical Vapor Deposition for Efficient Hydrogen Evolution Reaction. *Adv. Mater.* **2015**, *27* (37), 5605–5609. https://doi.org/10.1002/adma.201502075.
(34) Avilés, M. O.; Jelken, J.; Lagugné-Labarthet, F. Periodic Spiral Ripples on VS_2 Flakes: A Tip-Enhanced Raman Investigation. *J. Phys. Chem. Lett.* **2022**, *13* (41), 9771–9776. https://doi.org/10.1021/acs.jpclett.2c02555.
(35) Su, J.; Wang, M.; Li, Y.; Wang, F.; Chen, Q.; Luo, P.; Han, J.; Wang, S.; Li, H.; Zhai, T. Sub-Millimeter-Scale Monolayer p-Type H-Phase VS_2. *Adv. Funct. Mater.* **2020**, *30* (17), 2000240. https://doi.org/10.1002/adfm.202000240.
(36) Wang, X.; Ma, L.; Wang, C.; Wang, J.; Guo, J.; Tang, R.; Zhu, J.; Zou, G. Seed Engineering toward Layer-Regulated Growth of Magnetic Semiconductor VS2. *Adv. Funct. Mater.* **2023**, *33* (19), 2213295. https://doi.org/10.1002/adfm.202213295.
(37) Zhang, S.; Chang, P.; Zhang, Y.; Xu, X.; Guan, L.; Tao, J. Effect of Morphology and Stacking on Atomic Interaction and Magnetic Characteristics in Two-Dimensional H-Phase VS_2 Few Layers. *J. Mater. Sci.* **2022**, *57* (10), 5873–5884. https://doi.org/10.1007/s10853-022-06904-7.
(38) Liu, B.; Wang, L.; Zhu, Y.; Peng, H.; Du, C.; Yang, X.; Zhao, Q.; Hou, J.; Cao, C. Ammonium-Modified Synthesis of Vanadium Sulfide Nanosheet Assemblies toward High Sodium Storage. *ACS Nano* **2022**, *16* (8), 12900–12909. https://doi.org/10.1021/acsnano.2c05232.
(39) Liu, Q.; Yao, W.; Zhan, L.; Wang, Y.; Zhu, Y.-A. V3S4 Nanoparticles Anchored on Three-Dimensional Porous Graphene Gel for Superior Lithium Storage. *Electrochim. Acta* **2018**, *261*, 35–41. https://doi.org/10.1016/j.electacta.2017.10.137.

(40) Xu, Z.; Zhang, Y.; Wang, Y.; Zhan, L. Flower-like Nanostructured V3S4 Grown on Three-Dimensional Porous Graphene Aerogel for Efficient Oxygen Reduction Reaction. *App. Surf. Sci.* **2018**, *450*, 348–355. https://doi.org/10.1016/j.apsusc.2018.04.163.

(41) Yao, G.; Niu, P.; Li, Z.; Xu, Y.; Wei, L.; Niu, H.; Yang, Y.; Zheng, F.; Chen, Q. Construction of Flexible V_3S_4@CNF Films as Long-Term Stable Anodes for Sodium-Ion Batteries. *Chem. Eng. J.* **2021**, *423*, 130229. https://doi.org/10.1016/j.cej.2021.130229.

(42) Gopalakrishnan, D.; Lee, A.; Kumar Thangavel, N.; Arava, L. M. R. Facile Synthesis of Electrocatalytically Active NbS_2 Nanoflakes for an Enhanced Hydrogen Evolution Reaction (HER). *Sustain. Energy Fuels* **2018**, *2* (1), 96–102. https://doi.org/10.1039/C7SE00376E.

(43) Witteveen, C.; Górnicka, K.; Chang, J.; Månsson, M.; Klimczuk, T.; Rohr, F. O. von. Polytypism and Superconductivity in the NbS_2 System. *Dalton Trans.* **2021**, *50* (9), 3216–3223. https://doi.org/10.1039/D0DT03636F.

(44) McMullan, W. G.; Irwin, J. C. Raman Scattering from 2H and 3R–NbS_2. *Solid State Commun.* **1983**, *45* (7), 557–560. https://doi.org/10.1016/0038-1098(83)90426-X.

(45) Fisher, W. G.; Sienko, M. J. Stoichiometry, Structure, and Physical Properties of Niobium Disulfide. *Inorg. Chem.* **1980**, *19* (1), 39–43. https://doi.org/10.1021/ic50203a009.

(46) Leroux, M.; Cario, L.; Bosak, A.; Rodière, P. Traces of Charge Density Waves in NbS_2. *Phys. Rev. B* **2018**, *97* (19), 195140. https://doi.org/10.1103/PhysRevB.97.195140.

(47) Liao, Y.; Park, K.-S.; Singh, P.; Li, W.; Goodenough, J. B. Reinvestigation of the Electrochemical Lithium Intercalation in 2H- and 3R-NbS_2. *J. Power Sources* **2014**, *245*, 27–32. https://doi.org/10.1016/j.jpowsour.2013.06.048.

(48) Wang, X.; Lin, J.; Zhu, Y.; Luo, C.; Suenaga, K.; Cai, C.; Xie, L. Chemical Vapor Deposition of Trigonal Prismatic NbS_2 Monolayers and 3R-Polytype Few-Layers. *Nanoscale* **2017**, *9* (43), 16607–16611. https://doi.org/10.1039/C7NR05572B.

(49) Kozhakhmetov, A.; Choudhury, T. H.; Al Balushi, Z. Y.; Chubarov, M.; Redwing, J. M. Effect of Substrate on the Growth and Properties of Thin 3R NbS_2 Films Grown by Chemical Vapor Deposition. *J. Cryst. Growth* **2018**, *486*, 137–141. https://doi.org/10.1016/j.jcrysgro.2018.01.031.

(50) Nagaoka, D. A.; Grasseschi, D.; Cadore, A. R.; Fonsaca, J. E. S.; Jawaid, A. M.; Vaia, R. A.; De Matos, C. J. S. Redox Exfoliated NbS_2 : Characterization, Stability, and Oxidation. *Phys. Chem. Chem. Phys.* **2023**, *25* (13), 9559–9568. https://doi.org/10.1039/D2CP05197D.

(51) Ge, W.; Kawahara, K.; Tsuji, M.; Ago, H. Large-Scale Synthesis of NbS 2 Nanosheets with Controlled Orientation on Graphene by Ambient Pressure CVD. *Nanoscale* **2013**, *5* (13), 5773–5778. https://doi.org/10.1039/C3NR00723E.

(52) Dash, J. K.; Chen, L.; Dinolfo, P. H.; Lu, T.-M.; Wang, G.-C. A Method Toward Fabricating Semiconducting 3R-NbS_2 Ultrathin Films. *J. Phys. Chem. C* **2015**, *119* (34), 19763–19771. https://doi.org/10.1021/acs.jpcc.5b04057.

(53) Niazi, A.; Rastogi, A. K. Low-Temperature Resistance Minimum in Non-Superconducting 3R-$Nb_{1+x}S_2$ and 3R-Ga_xNbS_2. *Journal of Physics Condensed Matter* **2001**, *13* (31), 6787–6796. https://doi.org/10.1088/0953-8984/13/31/315.

(54) Najafi, L.; Bellani, S.; Oropesa-Nuñez, R.; Martín-García, B.; Prato, M.; Mazánek, V.; Debellis, D.; Lauciello, S.; Brescia, R.; Sofer, Z.; Bonaccorso, F. Niobium Disulphide (NbS_2)-Based (Heterogeneous) Electrocatalysts for an Efficient Hydrogen Evolution Reaction. *J. Mater. Chem. A* **2019**, *7* (44), 25593–25608. https://doi.org/10.1039/C9TA07210A.

(55) Li, Z.; Yang, W.; Losovyj, Y.; Chen, J.; Xu, E.; Liu, H.; Werbianskyj, M.; Fertig, H. A.; Ye, X.; Zhang, S. Large-Size Niobium Disulfide Nanoflakes down to Bilayers Grown by Sulfurization. *Nano Res.* **2018**, *11* (11), 5978–5988. https://doi.org/10.1007/s12274-018-2111-z.

(56) Zhou, X.; Lin, S.-H.; Yang, X.; Li, H.; Nejib Hedhili, M.; Li, L.-J.; Zhang, W.; Shi, Y. MoS_x -Coated NbS_2 Nanoflakes Grown on Glass Carbon: An Advanced Electrocatalyst for the Hydrogen Evolution Reaction. *Nanoscale* **2018**, *10* (7), 3444–3450. https://doi.org/10.1039/C7NR09172A.

(57) Zeng, Q.; Wang, X.; Xie, X.; Lu, G.; Wang, Y.; Cheng Lee, S.; Sun, J. TiO_2/TaS_2 with Superior Charge Separation and Adsorptive Capacity to the Photodegradation of Gaseous Acetaldehyde. *Chem. Eng. J.* **2020**, *379*, 122395. https://doi.org/10.1016/j.cej.2019.122395.
(58) Huan, Y.; Shi, J.; Zou, X.; Gong, Y.; Zhang, Z.; Li, M.; Zhao, L.; Xu, R.; Jiang, S.; Zhou, X.; Hong, M.; Xie, C.; Li, H.; Lang, X.; Zhang, Q.; Gu, L.; Yan, X.; Zhang, Y. Vertical 1T-TaS_2 Synthesis on Nanoporous Gold for High-Performance Electrocatalytic Applications. *Adv. Mater.* **2018**, *30* (15), 1705916. https://doi.org/10.1002/adma.201705916.
(59) Najafi, L.; Bellani, S.; Oropesa-Nuñez, R.; Martín-García, B.; Prato, M.; Pasquale, L.; Panda, J.-K.; Marvan, P.; Sofer, Z.; Bonaccorso, F. TaS_2 , $TaSe_2$, and Their Heterogeneous Films as Catalysts for the Hydrogen Evolution Reaction. *ACS Catal.* **2020**, *10* (5), 3313–3325. https://doi.org/10.1021/acscatal.9b03184.
(60) Najafi, L.; Bellani, S.; Oropesa-Nuñez, R.; Brescia, R.; Prato, M.; Pasquale, L.; Demirci, C.; Drago, F.; Martín-García, B.; Luxa, J.; Manna, L.; Sofer, Z.; Bonaccorso, F. Microwave-Induced Structural Engineering and Pt Trapping in 6R-TaS_2 for the Hydrogen Evolution Reaction. *Small* **2020**, *16* (50), 2003372. https://doi.org/10.1002/smll.202003372.
(61) Yu, Q.; Luo, Y.; Qiu, S.; Li, Q.; Cai, Z.; Zhang, Z.; Liu, J.; Sun, C.; Liu, B. Tuning the Hydrogen Evolution Performance of Metallic 2D Tantalum Disulfide by Interfacial Engineering. *ACS Nano* **2019**, *13* (10), 11874–11881. https://doi.org/10.1021/acsnano.9b05933.
(62) Kovalska, E.; Roy, P. K.; Antonatos, N.; Mazanek, V.; Vesely, M.; Wu, B.; Sofer, Z. Photocatalytic Activity of Twist-Angle Stacked 2D TaS_2. *npj 2D Mater. Appl.* **2021**, *5* (1), 68. https://doi.org/10.1038/s41699-021-00247-8.
(63) Feng, Y.; Gong, S.; Du, E.; Chen, X.; Qi, R.; Yu, K.; Zhu, Z. 3R TaS_2 Surpasses the Corresponding 1T and 2H Phases for the Hydrogen Evolution Reaction. *J. Phys. Chem. C* **2018**, *122* (4), 2382–2390. https://doi.org/10.1021/acs.jpcc.7b10833.
(64) Habib, M. R.; Wang, S.; Wang, W.; Xiao, H.; Obaidulla, S. M.; Gayen, A.; Khan, Y.; Chen, H.; Xu, M. Electronic Properties of Polymorphic Two-Dimensional Layered Chromium Disulphide. *Nanoscale* **2019**, *11* (42), 20123–20132. https://doi.org/10.1039/C9NR04449C.
(65) Zhou, S.; Wang, R.; Han, J.; Wang, D.; Li, H.; Gan, L.; Zhai, T. Ultrathin Non-van Der Waals Magnetic Rhombohedral Cr_2S_3: Space-Confined Chemical Vapor Deposition Synthesis and Raman Scattering Investigation. *Adv. Funct. Mater.* **2019**, *29* (3), 1805880. https://doi.org/10.1002/adfm.201805880.
(66) Cui, F.; Zhao, X.; Xu, J.; Tang, B.; Shang, Q.; Shi, J.; Huan, Y.; Liao, J.; Chen, Q.; Hou, Y.; Zhang, Q.; Pennycook, S. J.; Zhang, Y. Controlled Growth and Thickness-Dependent Conduction-Type Transition of 2D Ferrimagnetic Cr_2S_3 Semiconductors. *Adv. Mater.* **2020**, *32* (4), 1905896. https://doi.org/10.1002/adma.201905896.
(67) Zhang, Y.; Li, W.; France, L. J.; Chen, Z.; Zeng, Q.; Guo, D.; Li, X. Annealing Strategies for the Improvement of Low-Temperature NH_3-Selective Catalytic Reduction Activity of $CrMnO_x$ Catalysts. *ACS Omega* **2019**, *4* (5), 8681–8692. https://doi.org/10.1021/acsomega.9b00445.
(68) Gomes, A. S. O.; Yaghini, N.; Martinelli, A.; Ahlberg, E. A Micro-Raman Spectroscopic Study of Cr(OH)3 and Cr2O3 Nanoparticles Obtained by the Hydrothermal Method. *J. Raman Spectrosc.* **2017**, *48* (10), 1256–1263. https://doi.org/10.1002/jrs.5198.
(69) Shadike, Z.; Zhou, Y.-N.; Chen, L.-L.; Wu, Q.; Yue, J.-L.; Zhang, N.; Yang, X.-Q.; Gu, L.; Liu, X.-S.; Shi, S.-Q.; Fu, Z.-W. Antisite Occupation Induced Single Anionic Redox Chemistry and Structural Stabilization of Layered Sodium Chromium Sulfide. *Nat. Commun.* **2017**, *8* (1), 566. https://doi.org/10.1038/s41467-017-00677-3.
(70) Moinuddin, M. G.; Srinivasan, S.; Sharma, S. K. Probing Ferrimagnetic Semiconductor with Enhanced Negative Magnetoresistance: 2D Chromium Sulfide. *Adv. Electron. Mater.* **2021**, *7* (9), 2001116. https://doi.org/10.1002/aelm.202001116.
(71) Fu, H. Q.; Zhou, M.; Liu, P. F.; Liu, P.; Yin, H.; Sun, K. Z.; Yang, H. G.; Al-Mamun, M.; Hu, P.; Wang, H.-F.; Zhao, H. Hydrogen Spillover-Bridged Volmer/Tafel Processes Enabling Ampere-Level Current Density

Alkaline Hydrogen Evolution Reaction under Low Overpotential. *J. Am. Chem. Soc.* **2022**, *144* (13), 6028–6039. https://doi.org/10.1021/jacs.2c01094.
(72) Chakraborty, B.; Matte, H. S. S. R.; Sood, A. K.; Rao, C. N. R. Layer-Dependent Resonant Raman Scattering of a Few Layer MoS_2. *J. Raman Spectrosc.* **2013**, *44* (1), 92–96. https://doi.org/10.1002/jrs.4147.
(73) Kondekar, N. P.; Boebinger, M. G.; Woods, E. V.; McDowell, M. T. In Situ XPS Investigation of Transformations at Crystallographically Oriented MoS_2 Interfaces. *ACS Appl. Mater. Interfaces* **2017**, *9* (37), 32394–32404. https://doi.org/10.1021/acsami.7b10230.
(74) Gao, Z.-W.; Liu, M.; Zheng, W.; Zhang, X.; Lee, L. Y. S. Surface Engineering of MoS_2 via Laser-Induced Exfoliation in Protic Solvents. *Small* **2019**, *15* (44), 1903791. https://doi.org/10.1002/smll.201903791.
(75) Su, Q.; Wang, S.; Feng, M.; Du, G.; Xu, B. Direct Studies on the Lithium-Storage Mechanism of Molybdenum Disulfide. *Sci. Rep.* **2017**, *7* (1), 7275. https://doi.org/10.1038/s41598-017-07648-0.
(76) Shah, M. S.; Farrukh, S.; Douna, I.; Salahuddin, Z.; Hussain, A.; Sudais, A.; Pervaiz, E.; Pontie, M.; Ahmed, M. WS_2 Nanosheets Rooted in Polyethylene Terephthalate Membrane for Gas Barrier Properties Improvement. *J. Mater. Sci.* **2023**, *58* (11), 4753–4765. https://doi.org/10.1007/s10853-023-08165-4.
(77) Cheng, L.; Huang, W.; Gong, Q.; Liu, C.; Liu, Z.; Li, Y.; Dai, H. Ultrathin WS_2 Nanoflakes as a High-Performance Electrocatalyst for the Hydrogen Evolution Reaction. *Angew. Chem.* **2014**, *126* (30), 7994–7997. https://doi.org/10.1002/ange.201402315.
(78) del Corro, E.; Botello-Méndez, A.; Gillet, Y.; Elias, A. L.; Terrones, H.; Feng, S.; Fantini, C.; Rhodes, D.; Pradhan, N.; Balicas, L.; Gonze, X.; Charlier, J.-C.; Terrones, M.; Pimenta, M. A. Atypical Exciton–Phonon Interactions in WS_2 and WSe_2 Monolayers Revealed by Resonance Raman Spectroscopy. *Nano Lett.* **2016**, *16* (4), 2363–2368. https://doi.org/10.1021/acs.nanolett.5b05096.
(79) Rout, C. S.; Joshi, P. D.; Kashid, R. V.; Joag, D. S.; More, M. A.; Simbeck, A. J.; Washington, M.; Nayak, S. K.; Late, D. J. Superior Field Emission Properties of Layered WS_2-RGO Nanocomposites. *Sci. Rep.* **2013**, *3* (1), 3282. https://doi.org/10.1038/srep03282.
(80) Berkdemir, A.; Gutiérrez, H. R.; Botello-Méndez, A. R.; Perea-López, N.; Elías, A. L.; Chia, C.-I.; Wang, B.; Crespi, V. H.; López-Urías, F.; Charlier, J.-C.; Terrones, H.; Terrones, M. Identification of Individual and Few Layers of WS_2 Using Raman Spectroscopy. *Sci. Rep.* **2013**, *3* (1), 1755. https://doi.org/10.1038/srep01755.
(81) O'Brien, M.; Lee, K.; Morrish, R.; Berner, N. C.; McEvoy, N.; Wolden, C. A.; Duesberg, G. S. Plasma Assisted Synthesis of WS_2 for Gas Sensing Applications. *Chem. Phys. Lett.* **2014**, *615*, 6–10. https://doi.org/10.1016/j.cplett.2014.09.051.
(82) Voiry, D.; Yamaguchi, H.; Li, J.; Silva, R.; Alves, D. C. B.; Fujita, T.; Chen, M.; Asefa, T.; Shenoy, V. B.; Eda, G.; Chhowalla, M. Enhanced Catalytic Activity in Strained Chemically Exfoliated WS_2 Nanosheets for Hydrogen Evolution. *Nat. Mater.* **2013**, *12* (9), 850–855. https://doi.org/10.1038/nmat3700.
(83) Sun, K.; Fu, M.; Xie, Z.; Su, D.; Zhong, H.; Bai, J.; Dooryhee, E.; Gan, H. Improvement of Li-S Battery Electrochemical Performance with 2D TiS_2 Additive. *Electrochim. Acta* **2018**, *292*, 779–788. https://doi.org/10.1016/j.electacta.2018.09.191.
(84) Kadam, S. R.; Kawade, U. V.; Bar-Ziv, R.; Gosavi, S. W.; Bar-Sadan, M.; Kale, B. B. Porous MoS_2 Framework and Its Functionality for Electrochemical Hydrogen Evolution Reaction and Lithium Ion Batteries. *ACS Appl. Energy Mater.* **2019**, *2* (8), 5900–5908. https://doi.org/10.1021/acsaem.9b01045.
(85) Zou, J.-P.; Ma, J.; Luo, J.-M.; Yu, J.; He, J.; Meng, Y.; Luo, Z.; Bao, S.-K.; Liu, H.-L.; Luo, S.-L.; Luo, X.-B.; Chen, T.-C.; Suib, S. L. Fabrication of Novel Heterostructured Few Layered WS_2-Bi_2WO_6/$Bi_{3.84}W_{0.16}O_{6.24}$ Composites with Enhanced Photocatalytic Performance. *Appl. Catal. B* **2015**, *179*, 220–228. https://doi.org/10.1016/j.apcatb.2015.05.031.
(86) Sherrell, P. C.; Sharda, K.; Grotta, C.; Ranalli, J.; Sokolikova, M. S.; Pesci, F. M.; Palczynski, P.; Bemmer, V. L.; Mattevi, C. Thickness-Dependent Characterization of Chemically Exfoliated TiS2 Nanosheets. *ACS Omega* **2018**, *3* (8), 8655–8662.
(87) Mattinen, M.; Popov, G.; Vehkamäki, M.; King, P. J.; Mizohata, K.; Jalkanen, P.; Räisänen, J.; Leskelä, M.; Ritala, M. Atomic Layer Deposition of Emerging 2D Semiconductors, HfS2 and ZrS2, for Optoelectronics. *Chem. Mater.* **2019**, *31* (15), 5713–5724.

(88) Ng, S.; Ghosh, K.; Vyskocil, J.; Pumera, M. Two-Dimensional Vanadium Sulfide Flexible Graphite/Polymer Films for near-Infrared Photoelectrocatalysis and Electrochemical Energy Storage. *Chem. Eng. J.* **2022**, *435*, 135131.
(89) Wang, X.; Lin, J.; Zhu, Y.; Luo, C.; Suenaga, K.; Cai, C.; Xie, L. Chemical Vapor Deposition of Trigonal Prismatic NbS2 Monolayers and 3R-Polytype Few-Layers. *Nanoscale* **2017**, *9* (43), 16607–16611.
(90) Yao, B.; Liu, W.; Zhou, X.; Yang, J.; Huang, X.; Fu, Z.; Yuan, G.; Nie, Y.; Dai, Y.; Xu, J.; Gao, L. Growth of Wafer-Scale Chromium Sulphide and Selenide Semiconductor Films. *J. Phys.: Condens. Matter* **2023**, *35* (33), 335302.
(91) Pierucci, D.; Henck, H.; Naylor, C. H.; Sediri, H.; Lhuillier, E.; Balan, A.; Rault, J. E.; Dappe, Y. J.; Bertran, F.; Fèvre, P. L.; Johnson, A. T. C.; Ouerghi, A. Large Area Molybdenum Disulphide- Epitaxial Graphene Vertical Van Der Waals Heterostructures. *Sci Rep* **2016**, *6* (1), 26656.
(92) Zeng, H.; Liu, G.-B.; Dai, J.; Yan, Y.; Zhu, B.; He, R.; Xie, L.; Xu, S.; Chen, X.; Yao, W.; Cui, X. Optical Signature of Symmetry Variations and Spin-Valley Coupling in Atomically Thin Tungsten Dichalcogenides. *Sci Rep* **2013**, *3* (1), 1608.
